# Materials Containing Single‐, Di‐, Tri‐, and Multi‐Metal Atoms Bonded to C, N, S, P, B, and O Species as Advanced Catalysts for Energy, Sensor, and Biomedical Applications

**DOI:** 10.1002/advs.202403197

**Published:** 2024-07-01

**Authors:** Jitendra N. Tiwari, Krishan Kumar, Moein Safarkhani, Muhammad Umer, A. T. Ezhil Vilian, Ana Beloqui, Gokul Bhaskaran, Yun Suk Huh, Young‐Kyu Han

**Affiliations:** ^1^ Department of Energy and Materials Engineering Dongguk University‐Seoul Seoul 100715 Republic of Korea; ^2^ POLYMAT Applied Chemistry Department Faculty of Chemistry University of the Basque Country UPV/EHU Paseo Manuel de Lardizabal 3 Danostia‐San Sebastian 20018 Spain; ^3^ Department of Biological Sciences and Bioengineering Nano Bio High‐Tech Materials Research Center Inha University Incheon 22212 Republic of Korea; ^4^ Bernal Institute Department of Chemical Sciences University of Limerick Limerick V94 T9PX Republic of Ireland; ^5^ IKERBASQUE Basque Foundation for Science Plaza Euskadi 5 Bilbao 48009 Spain; ^6^ School of Chemistry Damghan University Damghan 36716‐45667 Iran

**Keywords:** artificial enzymes, energy storage and conversion devices, H_2_O_2_ production, hydrogen and oxygen evolution reactions, N_2_ and CO_2_ reduction reactions, sensors, transition‐metal atoms

## Abstract

Modifying the coordination or local environments of single‐, di‐, tri‐, and multi‐metal atom (SMA/DMA/TMA/MMA)‐based materials is one of the best strategies for increasing the catalytic activities, selectivity, and long‐term durability of these materials. Advanced sheet materials supported by metal atom‐based materials have become a critical topic in the fields of renewable energy conversion systems, storage devices, sensors, and biomedicine owing to the maximum atom utilization efficiency, precisely located metal centers, specific electron configurations, unique reactivity, and precise chemical tunability. Several sheet materials offer excellent support for metal atom‐based materials and are attractive for applications in energy, sensors, and medical research, such as in oxygen reduction, oxygen production, hydrogen generation, fuel production, selective chemical detection, and enzymatic reactions. The strong metal–metal and metal–carbon with metal–heteroatom (i.e., N, S, P, B, and O) bonds stabilize and optimize the electronic structures of the metal atoms due to strong interfacial interactions, yielding excellent catalytic activities. These materials provide excellent models for understanding the fundamental problems with multistep chemical reactions. This review summarizes the substrate structure‐activity relationship of metal atom‐based materials with different active sites based on experimental and theoretical data. Additionally, the new synthesis procedures, physicochemical characterizations, and energy and biomedical applications are discussed. Finally, the remaining challenges in developing efficient SMA/DMA/TMA/MMA‐based materials are presented.

## Graphical abstract

Herein, the recent progress of the synthesis procedures, physicochemical characterizations, and computational models of single‐, di‐, tri‐, and multi‐metal atom (SMA, DMA, TMA, and MMA, respectively) sites coordinated with different species are examined, summarized, and discussed. Additionally, the energy, sensing, and biomedical applications of these materials are comprehensively delineated. Finally, the challenges and prospects of these materials are presented.

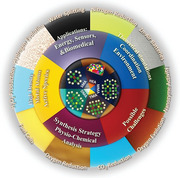

## 1 Introduction

**Figure 1:**
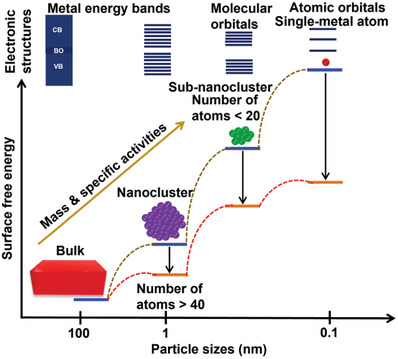
Diagram showing the relationships among surface free energy, mass, and specific activities of SMAs. Surface free energy is directly related to the metal sites in the metallic components. High surface free energies of the metallic components produce several active metal sites for chemical interactions with the support materials. Discrete energy levels appear at the band‐edges. VB, valence band; BO, band overlap; and CB, conduction band.

Various metal‐based nanocatalysts for energy, sensor, and biomedical applications have been reported.^[^ ^1^, ^2^, ^3^, ^4^, ^5^, ^6^, ^7^, ^8^, ^9^, ^10^ ^]^ Among these nanocatalysts, single‐ to multi‐metallic coordinated C, N, S, P, B, and O atoms are crucial for developing low‐cost, high‐performance energy conversion technologies, efficient energy storage devices, and devices with enzymatic activities, e.g., water electrolyzers, fuel cells, Zn‐air batteries (ZABs), chemicals sensors, gas sensors, biosensors, and devices used for biotherapy.^[^ ^1^, ^2^, ^3^, ^4^, ^5^, ^6^, ^7^, ^8^, ^9^, ^10^, ^11^, ^12^, ^13^, ^14^, ^15^, ^16^, ^17^, ^18^, ^19^, ^20^, ^21^, ^22^, ^23^ ^]^ Currently, single‐, di‐, tri‐, and multi‐metal atoms (SMAs, DMAs, TMAs, and MMAs, respectively) coordinated to carbon and other electron‐donor atoms (e.g., N, S, P, B, and O) yield high catalytic activity and selectivity and can be easily prepared. Additionally, stability is achieved in terms of four characteristics, including a low coordination environment, the quantum size effect, electronic modulation, and metal‐strut interactions.^[^ ^11^, ^12^, ^13^, ^14^, ^15^, ^16^, ^17^, ^18^, ^19^, ^20^, ^21^, ^22^, ^23^, ^24^, ^25^, ^26^, ^27^, ^28^, ^29^, ^30^, ^31^, ^32^, ^33^, ^34^ ^]^ Furthermore, the surface free energies increase substantially when the sizes of the particles and/or clusters reduce to those of single atoms, resulting in the aggregation of the SMAs, DMAs, TMAs, and MMAs into large particles during the fabrication process (**Figure** **1**).^[^ ^33^, ^35^ ^]^ Therefore, suitable supports on which these metal atoms can be well distributed, anchored, and stabilized must be identified. Studies on SMA/DMA/TMA/MMA‐based materials have revealed that the development of these materials is unpredictable and rapid, facilitating homogeneous and heterogeneous reduction catalysis.^[^ ^11^, ^12^, ^13^, ^14^, ^15^, ^16^, ^17^, ^18^, ^19^, ^20^, ^21^, ^22^, ^23^, ^24^, ^25^, ^26^, ^27^, ^28^, ^29^, ^30^, ^31^, ^32^, ^33^, ^36^, ^37^ ^]^

**Figure 2:**
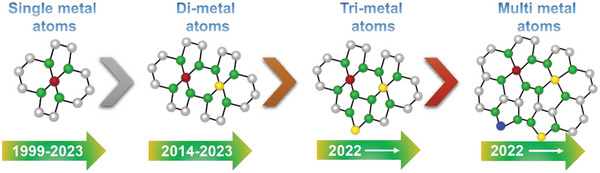
Historical developments of SMA/DMA/TMA/MMA‐based materials.

**Figure** **2** presents the historical development of the SMA/DMA/TMA/MMA‐based materials.^[^ ^38^, ^39^ ^]^ Owing to their single‐, double‐, triple‐, and multiple active sites and ultra‐stable structures, these materials have been identified as the intermediate between homogeneous and heterogeneous catalysts.^[^ ^11^, ^12^, ^13^, ^14^, ^15^, ^16^, ^17^, ^18^, ^19^, ^20^, ^21^, ^22^, ^23^, ^24^, ^25^, ^26^, ^27^, ^28^, ^29^, ^30^, ^31^, ^32^, ^33^, ^36^, ^37^ ^]^

SMA/DMA/TMA/MMA‐based materials are fabricated for various applications, including in the hydrogen evolution reaction (HER), oxygen evolution reaction (OER), water electrolyzers, oxygen reduction reaction (ORR), fuel cells, ZABs, producing H_2_O_2_, N_2_ reduction reaction, CO_2_ reduction reaction (CO_2_RR), chemical sensors, gas sensors, biosensors, and enhancing enzymatic activities. Molecular H_2_ and O_2_ are produced by water splitting in a process comprising two semi‐reactions, namely HER and OER. These semi‐reactions are kinetically slow, and an overpotential is required on the nanocatalysts to evolve H_2_ and O_2_ at the cathode and anode, respectively (further details are present in the portion on HER and OER applications). The efficiencies of ZABs mainly depend on the two half‐reactions: the ORR on one side and the OER on the other side.^[^ ^40^, ^41^, ^42^, ^43^ ^]^ In ZABs, the OER and ORR are half‐reactions of decomposed H_2_O and the fuel battery, respectively. Therefore, the reversibility of the ORR and OER reactions is crucial for obtaining high‐performance ZABs. All electrocatalytic oxidation and/or reduction reactions depend on the development of highly efficient electrocatalysts. **Table** **1** lists the general conversion reactions in various applications.^[^ ^1^, ^2^, ^3^, ^4^, ^5^, ^6^, ^7^, ^8^, ^9^, ^10^, ^11^, ^12^, ^13^, ^14^, ^15^, ^16^, ^17^, ^18^, ^19^, ^20^, ^21^, ^22^, ^23^, ^25^, ^26^, ^44^, ^45^, ^46^, ^47^ ^]^

**Table 1:** Various chemical reactions involved in water splitting, fuel cells, ZABs, valuable chemicals, and fuel generation.

Electrochemical reactions	Mechanisms	Reaction media
HER	H^+^ + e^−^ → H_ads_; Volmer step H_ads_ + H_ads_→ H_2_(*g*); Volmer‐Tafel H_ads_ + H^+^ + e^−^ → H_2_(*g*); Volmer‐Heyrovsky H_2_O (*l*) +e^−^ →H_ads_ + OH^−^; Volmer step H_ads_ + H_ads_→ H_2_(*g*); Volmer‐Tafel H_ads_ + H^+^ + e^−^ → H_2_(*g*); Volmer‐Heyrovsky	Acidic Acidic
OER	2H_2_O(*l*) → O_2_(*g*) + 4H^+^+ 4e^−^ 4OH^−^ → O_2_(*g*) + 2H_2_O(*l*) + 4e^−^	Alkaline Acidic
ORR	O_2_ + H_2_O (*l*) + 2e^−^ → HO_2_^−^ + OH^−^ O_2_ + 2H_2_O(*l*) + 4e^−^ → 4OH^−^ O_2_ + 2H^+^ + 2e^−^ → H_2_O_2_ O_2_ + 4H^+^ + 4e^−^ → 2H_2_O	Alkaline Alkaline Acidic Acidic
H_2_O_2_	2H_2_O(*l*) ⇌ H_2_O_2_ + 2H^+^ + 2e^−^ O_2_ + 2H^+^ + 2e^−^ ⇌ H_2_O_2_	‐ Acidic
CO_2_RR	CO_2_(*g*) + 2H^+^ + 2e^−^ = HCOOH CO_2_(*g*) + 2H^+^ + 2e^−^ = CO(*g*) + H_2_O(*l*) CO_2_(*g*) + 4H^+^ + 4e^−^ = CH_2_O(*l*) + H_2_O(*l*) CO_2_(*g*) + 6H^+^ + 6e^−^ = CH_3_OH(*l*) + H_2_O(*l*) CO_2_(*g*) + 8H^+^ + 8e^−^ = CH_4_(*g*) + 2H_2_O (*l*) 2CO_2_(*g*) + 12H^+^ + 12e^−^ = CH_3_CH_2_OH(*l*) + 3H_2_O(*l*)	– – – – – –
NRR	N_2_ + 6H^+^ + 6e^−^ → 2NH_3_ N_2_ + 6H_2_O + 6e^−^ → 2NH_3_ + 6OH^−^	Acidic Alkaline

SMA/DMA/TMA/MMA‐based materials possess large surface areas, abundant catalytic sites, high electrical and ionic conductivity, long‐term configuration durability, and enhanced charge transfer efficiencies with respect to nanoparticles and bulk materials.^[^ ^11^, ^12^, ^13^, ^14^, ^15^, ^16^, ^17^, ^18^, ^19^, ^20^, ^21^, ^22^, ^23^, ^24^, ^25^, ^26^, ^27^, ^28^, ^29^, ^30^, ^31^, ^32^, ^33^ ^]^ The development of SMA/DMA/TMA/MMA‐based materials has presented new and promising opportunities to the energy, sensor, and biomedical fields.^[^ ^11^, ^12^, ^13^, ^14^, ^15^, ^16^, ^17^, ^18^, ^19^, ^20^, ^21^, ^22^, ^23^, ^24^, ^25^, ^26^, ^27^, ^28^, ^29^, ^30^, ^31^, ^32^, ^33^, ^36^, ^37^ ^]^ In particular, DMA, TMA, and MMA‐coordinated C, N, S, P, B, and O species are garnering considerable attention for application in different catalytic reactions.^[^ ^11^, ^12^, ^13^, ^14^, ^15^, ^16^, ^17^, ^18^, ^19^, ^20^, ^21^, ^22^, ^23^, ^24^, ^25^, ^26^, ^27^, ^28^, ^29^, ^30^, ^31^, ^32^, ^33^, ^36^, ^37^ ^]^ Therefore, the most recent progress of C, N, S, P, B, and O species bonded to DMAs, TMAs, and MMAs must be investigated to facilitate the advancement in the design and synthesis of advanced catalysts. Herein, we discuss the progress in the development of SMA/DMA/TMA/MMA‐based catalysts used for energy, sensing, and biomedical applications. Additionally, we discuss the basic principles related to half‐cell reactions, which involve different types of value‐added chemicals. The synthesis and applications of these catalysts are subsequently explored in detail. Next, the industrial applications of these catalysts are highlighted. Finally, the challenges and perspectives on these catalysts used for energy, sensor, and biomedical applications are discussed.

## 2 Scope of this Review

**Scheme 1:**
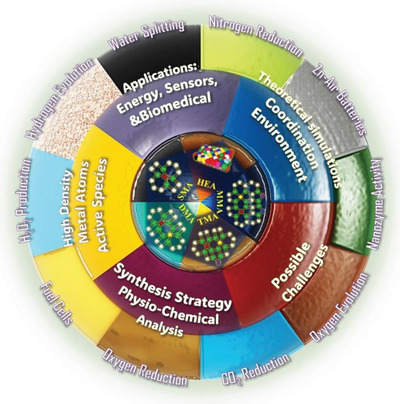
Development of SMA/DMA/TMA/MMA‐based materials for energy, sensor, and biomedical applications.

This review discusses the future scope of SMA/DMA/TMA/MMA‐based materials used for energy, sensor, and biomedical applications and assesses the potential real‐life uses of these materials (**Scheme** **1**). Furthermore, advanced approaches for preparing SMAs, DMAs, TMAs, and MMAs coordinated with C, N, S, P, B, and O species are briefly discussed (Sections 3 and 4). Subsequently, this review discusses the challenges encountered during the preparation of SMAs, DMAs, TMAs, and MMAs coordinated with C, N, S, P, B, and O species. The characterization techniques and analyses used for these materials are then reviewed (Section 5). Section 5.1 presents the coordination environments related to SMAs, DMAs, TMAs, and MMAs anchored in two‐dimensional (2D) materials (e.g., carbon sheets, graphene, and non‐carbon materials) and describes the corresponding electronic properties. Section 5.2 discusses the problems associated with interpreting catalytic sites. The recent advances in the structures, configurations, and performance relationships of these catalysts are reviewed, and further details regarding the uses of these catalysts in energy, sensor, and biomedical applications are presented (Section 6). The effects of the coordination environments on the catalytic properties are revealed, and the change in the energy of the reaction pathway is discussed based on theoretical (density functional theory (DFT)) and experimental studies. The final section presents the conclusions with new insights into various challenges related to these catalysts. Different research perspectives on these materials are discussed, offering new directions regarding the mass production and commercial applications of these materials (Section 7).

## 3 Techniques for Fabrication of Single‐, Di‐, Tri‐, and Multi‐Metal Atom (SMAs/DMAs/TMAs/MMAs) Sites Coordinated with Different Species

Several techniques have been used for the synthesis of SMAs, DMAs, TMAs, and MMAs, including wet synthesis, hydrothermal, solvothermal, mechanochemical ball‐milling, photoreduction, chemical vapor deposition (CVD), atomic layer deposition (ALD), trapping, laser ablation, arc‐discharge, electrochemical deposition, pyrolysis, and thermal emitting processes. This section discusses the details of these techniques.

### 3.1 Wet Synthetic Strategy

Wet synthesis is a simple, surfactant‐free, and large‐scale preparation route that includes a variety of processing techniques, such as impregnation, adsorption, coprecipitation, and strong electrostatic adsorption.^[^ ^48^, ^49^, ^50^, ^51^, ^52^, ^53^ ^]^ These techniques are commonly preferred for the commercial production of supported SMA/DMA/TMA/MMA‐based materials. These methods are discussed in detail based on their recent reviews. Successful atomic‐level doping is governed by several crucial factors, such as selecting suitable metal precursors that ensure atomic‐level dispersion and chemical reactivity and the compatibility between the metal ions and the support. Successful atomic‐level doping should prevent the migration of atoms through the support, thus forming clusters. This can be achieved by trapping metal ions in the atomic defects present on the support. Physical constraints can limit the movement of metal atoms by using specific coordination sites that selectively anchor the metal atoms and by reducing the thermal mobility of ions by synthesizing the materials under low‐temperature conditions.

#### 3.1.1 Impregnation

An aqueous or organic metal‐salt solution is mixed with a carbon or non‐carbon support to anchor organometallic complexes or inorganic salts to the support. The synthesis strategy includes two steps: 1) absorbing metal ions onto the surface of the support and 2) drying or roasting to activate and enhance metal‐substrate interactions. The atoms are anchored to the support either by adsorption or an ion‐exchange mechanism. Thus, the properties of the SMAs, DMAs, TMAs, and MMAs synthesized via impregnation rely on the dispersion and interaction of the metal precursor adsorbed on the support surface. This method is economically efficient and simple; however, the loading capacity of the support relies on the functional groups or defects on the substrate surface.

#### 3.1.2 Adsorption

Similar to the impregnation process, the singly to multiply dispersed metal‐active sites are embedded in the 2D or 3D material supports using a simple adsorption method. The substrate is stirred with well‐dispersed metal precursors, and the filtrate is dried to obtain the catalyst. Metal atoms are generally adsorbed onto the surface functionalities, defect sites, or pore channels and remain attached after drying. The metal‐support attachment strongly depends on the surface properties of the support material. Chorkendorff et al.^[^ ^51^ ^]^ used the adsorption method to fabricate Pd single atoms embedded in N‐doped carbon spheres (denoted as Pd_1_/N‐C). These Pd sites were stabilized by six coordinating pyridinic N atoms.

#### 3.1.3 Coprecipitation

Coprecipitation is a conventional method that is the most frequently used to synthesize SMA/DMA/TMA/MMA‐based materials, and the resulting products are precipitated from a solution containing other ions. Coprecipitation is conducted by adding other external agents or altering environmental conditions such as temperature, pH, and light. The formation of clusters is highly probable when the reactions are conducted under high pH, temperature, and concentration conditions. Coprecipitation is a suitable method for the synthesis of single‐atomic catalysts; however, the simultaneous precipitation of multiple ions yields poor reproducibility because the synthesis conditions cannot be maintained. The principal mechanisms of coprecipitation are the same as those of the adsorption method.

#### 3.1.4 Strong Electrostatic Adsorption

**Figure 3:**
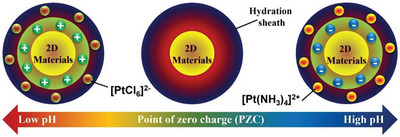
Illustration of the strong electrostatic adsorption method implemented over a wide pH range of 0–14.^[^ ^53^ ^]^

The surfaces of carbon or non‐carbon materials generally possess hydroxyl functional groups, making these surfaces positively or negatively charged in aqueous solutions in which the pH is higher or lower than the isoelectric point (the total charge on the surfaces of both carbon and non‐carbon materials is zero) (**Figure** **3**). Therefore, O^−^ and ^•^OH functional groups, controlled by pH, are formed on the surfaces of carbon and non‐carbon materials. These functional groups can attach to different metal ion precursors through strong electrostatic interactions. Strong electrostatic adsorption strongly anchors atoms, unlike the adsorption method in which the metal atoms attach to the defect sites and pores. During the reaction, the pH values of the aqueous solution may change, further affecting the formation of single‐atom catalysts (SACs). Examples of ions produced from metal salts are [Ru^3+^] or [Cl^−^], [(NH_3_)_4_Pt]^2+^, [H^+^] or [PtCl_6_]^2−^, [H^+^] or [IrCl_6_]^2−^, and [H^+^] or [PdCl_4_]^2−^. SMA/DMA/TMA/MMA‐based materials are obtained after eliminating the ligands of the metal salts during the second‐step treatment.

### 3.2 Hydrothermal and Solvothermal Processes

**Figure 4:**
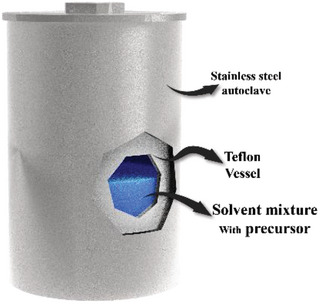
Setups of the hydrothermal and solvothermal processes.

Hydrothermal and solvothermal processes (**Figure** **4**) have attracted considerable attention because of their low cost, efficiency, versatility, cleanliness, and simplicity associated with synthesizing various materials. The experimental setups required for both these processes are similar. The synthesis reaction proceeds in an autoclave under subcritical and supercritical conditions.^[^ ^54^, ^55^, ^56^ ^]^ The reactant solution is filled in a Teflon liner, which is further enclosed in a stainless‐steel autoclave (Figure 4). In the hydrothermal process, the solution is heated to a high temperature, generally over 100 °C (above the boiling point of water), in a muffle furnace for a specified period to obtain the product. The high temperature and pressure conditions facilitate the synthesis of nanosized active materials. This process allows the ions on a template to be substituted with the ions in the reactant solution, thus depositing single atoms. Notably, hydrothermal and solvothermal processes are unsuitable for synthesizing all metals. The probability of atomic deposition strictly depends on both the reactivity of the template and the corresponding metal atom. The only difference between the two processes is the solvent used for material synthesis. Water and organic solvents are used in the hydrothermal and solvothermal processes, respectively.

### 3.3 Mechanochemical Ball‐Milling

**Figure 5:**
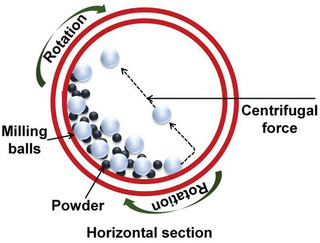
Schematic of the ball‐milling process for material preparation.

In the ball‐milling process (**Figure** **5**), a powdered mixture is placed in a ball mill and subjected to high‐energy collisions from the grinding balls (made of Fe or Zr).^[^ ^57^, ^58^, ^59^ ^]^ This is a mechanical process in which bulk materials are broken down into small structures. This method can induce the breaking and rejoining of chemical bonds, which can be specifically used for depositing metal atoms. Ball milling can be conducted in the presence or absence of a solvent. In this process, the defect sites formed on the support during high‐intensity mechanical grinding can be used as active sites to trap metal ions. Therefore, structural and chemical changes occur owing to mechanical energy.^[^ ^57^, ^58^, ^59^, ^60^, ^61^ ^]^ The ratio between the ball and the synthesized materials should be optimized because the ball‐to‐powder ratio affects the chemical and physical properties of the products.

### 3.4 Photoreduction

**Figure 6:**
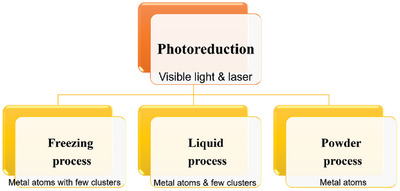
Various types of photoreduction processes.

The photoreduction process (**Figure** **6**) uses light energy as the main driving force to reduce metal precursors to their metallic states.^[^ ^62^, ^63^, ^64^, ^65^, ^66^, ^67^, ^68^ ^]^ This process does not require any specific equipment and is straightforward to implement, allowing industrial‐scale production. The space between the light source and the hybrid materials must be optimized to obtain metal atoms. The main drawback of this process is that the uniformly distributed metal atoms must be controlled due to the presence of a limited number of defects and/or vacancies on the surfaces of 2D materials.

### 3.5 Atomic Layer Deposition

**Figure 7:**
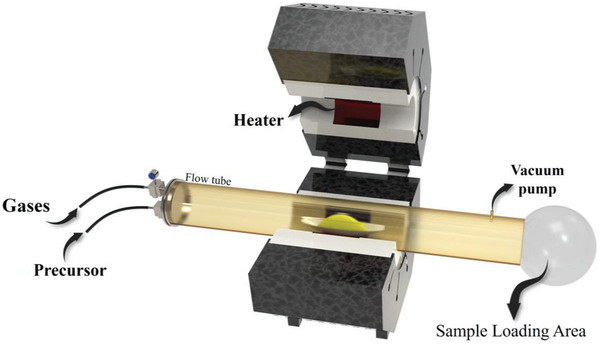
Schematic of an ALD reactor.

ALD (**Figure** **7**) is an efficient method for synthesizing metal atoms. In this method, the precursor vapor (gaseous form) is diffused into the reaction chamber, and the desired material is formed through surface chemical reactions.^[^ ^69^, ^70^ ^]^ The chamber consists of a static and rotary bed of substrates. The main attribute of this method is that the metal salts are pulsed alternately. Therefore, the deposition of the metal atoms can be controlled by adjusting the number of applied cycles. The main advantage of this process includes the effective prevention of the single metal atoms from aggregating during synthesis. Suitable ligands or functional groups on the surfaces of 2D materials are required to prepare metal atoms. The interaction between the support and the metal ions is important to ensure successful synthesis, thus synthesizing all metals may not be possible using the same support. The low deposition rate and high cost of production reduce the commercialization potential of this method.

### 3.6 Chemical Vapor Deposition

**Figure 8:**
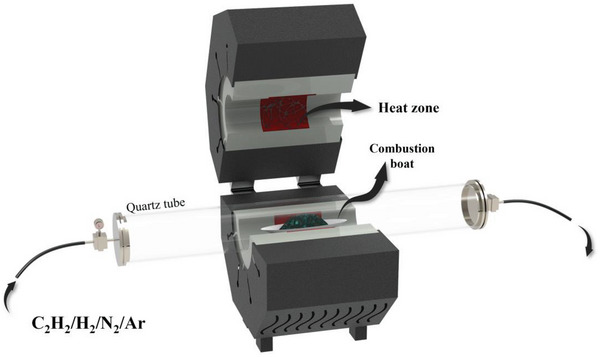
Schematic of the CVD setup.

Thermal CVD involves the reaction of an evaporated precursor that is introduced into a quartz tube furnace in the presence of C_2_H_2_, H_2_, N_2_, and Ar gases.^[^ ^71^, ^72^, ^73^ ^]^ The volatile reactants are deposited onto the defective surfaces of 2D materials. The reactant materials are heated to temperatures higher than their boiling points, and the gaseous metal atoms are carried by a carrier gas and deposited on the defective sites in the support. Both the support and the metal are placed in the quartz tube at a specified distance. The deposition can be controlled by controlling reaction time and temperature. **Figure** **8** displays a schematic of the CVD setup. This technique can be used in the temperature range of 400–650 °C under various heating rates.

### 3.7 Laser Ablation

**Figure 9:**
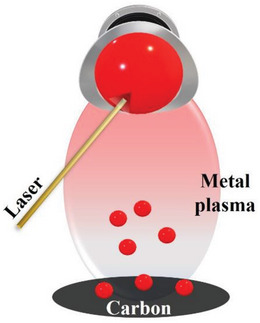
Diagram showing the laser ablation process.

Laser ablation synthesis (**Figure** **9**) is attracting increasing attention because of the availability of different wavelengths, high energy density, fast scanning speed, and high spatial resolution for fabricating metal atoms.^[^ ^74^, ^75^, ^76^, ^77^ ^]^ Laser irradiation removes metal atoms from metal‐foil targets and dopes 2D‐material surfaces.^[^ ^77^ ^]^ This process can be conducted in different atmospheres, such as air, vacuum, and liquid atmospheres, each with associated advantages and disadvantages.^[^ ^74^, ^75^, ^76^, ^77^ ^]^ Metal atoms produced in ultra‐high vacuum have superb purity and reactivity; however, the vacuum system required is expensive. Although air is an inexpensive environment, toxic compounds are produced when laser ablation is conducted in air. Furthermore, metal atoms obtained in air are impure owing to the impurities present in the air. Liquid environments are the best suited for fabricating metal atoms because of the availability of uncapped and capped products, which facilitate chemical interactions between the target materials and liquids.

### 3.8 Trapping

**Figure 10:**
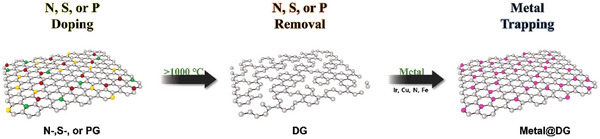
Trapping strategy for the formation of metal atoms. PG: P‐doped graphene and DG: defective graphene.

Trapping is a top‐down approach in which isolated metal atoms are trapped onto defective carbon or non‐carbon supports.^[^ ^78^, ^79^, ^80^ ^]^ During this process, metal salts are mixed with defective carbon or non‐carbon materials and heated at a certain temperature to facilitate bonding between the metal and C, N, S, and P species on an appropriate support (**Figure** **10**).^[^ ^78^ ^]^ The support, containing volatile atoms, is heated to a high temperature to generate defects, in which the metal atoms are trapped. This approach prevents the aggregation of metal atoms as they are trapped in specified confinements. Trapping can only be successful if the sizes of the defect sites match the radii of the active metal ions.

### 3.9 Thermal Emitting

**Figure 11:**
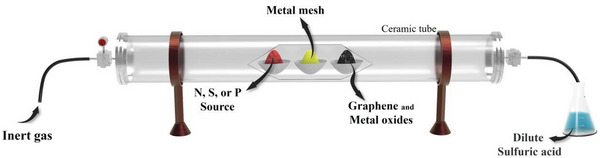
Setup of the thermal emitting process.

The thermal emitting process (**Figure** **11**) is known as atomic migration at high temperatures. This is a cost‐effective strategy for preparing single‐atom nanocatalysts from bulk materials. N, S, or P source materials, metallic meshes, graphene, and metal oxides are placed successively in a ceramic boat,^[^ ^81^ ^]^ which is heated to over 1000 °C in an inert‐gas atmosphere. The source materials are pyrolyzed to produce heteroatoms in this atmosphere under high temperatures. Volatile metal atoms from the metallic mesh that are bound to the gaseous species are released, and these metallic species are finally trapped by the defective graphene and metal oxides.

### 3.10 Arc‐Discharge Process

**Figure 12:**
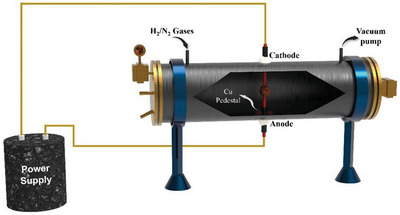
Schematic of the arc‐discharge setup.

Arc‐discharge deposition (**Figure** **12**) is one of the oldest coatings methods in which highly pure two‐graphite electrodes are used as the cathode and anode.^[^ ^82^ ^]^ The space between the two electrodes is a few millimeters. The anode is loaded with carbon and metal precursors that are deposited on the cathode substrate using a high‐power source (alternating or direct current).^[^ ^82^ ^]^ Typically, this process is completed within a few minutes, which makes it faster than other synthesis processes. The catalysts formed show excellent temperature stability. However, this process is in the early stages of development and needs further improvement for commercialization.

### 3.11 Electrochemical Deposition

**Figure 13:**
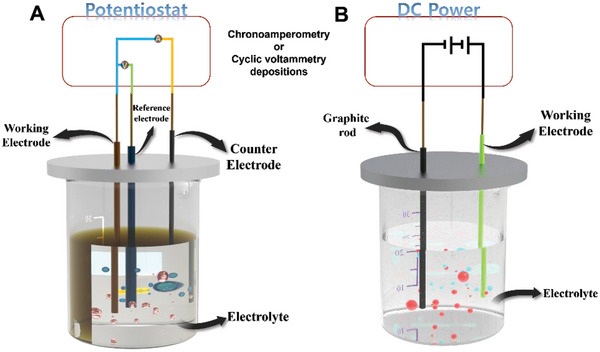
A) Three‐ and B) two‐electrode configurations for depositing SMAs, DMAs, TMAs, and MMAs on substrate materials.

Electrochemical deposition is an easy and useful technique for depositing materials and modifying the surface properties of working electrode materials. This process has several advantages, e.g., simple preparation, low energy utilization, cost‐effectiveness, and eco‐friendliness. Electrodeposition is divided into three‐ and two‐electrode configurations (**Figure** **13**). In the three‐electrode configuration, two main electrochemical processes, i.e., direct‐current electrodeposition and the potential cycling process, occur when synthesizing SMAs, DMAs, TMAs, and MMAs (Figure 13A).^[^ ^83^ ^]^ The two‐electrode configuration is achieved by applying a direct current between the working and auxiliary electrodes (Figure 13B).^[^ ^84^ ^]^ Metal ions can be selectively deposited on the substrate by setting the corresponding reduction potentials of the metals. Electrodeposition is a fast process that is completed within minutes. The reproducibility of this process is strictly controlled by the repeatability of the chemical and physical conditions during the reaction.

### 3.12 Pyrolysis

**Figure 14:**
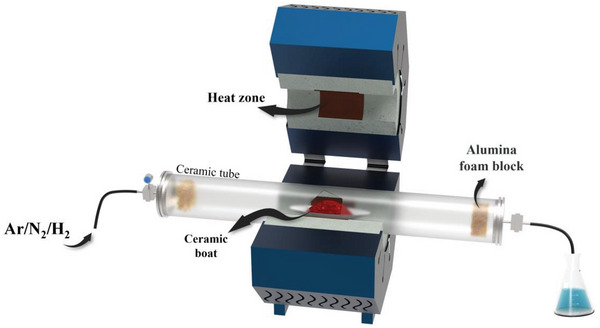
Schematic of the pyrolysis process.

Pyrolysis is a crucial process for fabricating SMAs, DMAs, TMAs, and MMAs via the thermochemical decomposition of organic‐inorganic hybrid precursors at high temperatures in Ar, N_2_, or H_2_ atmospheres. The gas used in the system depends on the reaction, e.g., unreactive atmospheres are required in some cases and the gas needs to participate in the reduction or oxidation of the precursors in other cases. **Figure** **14** presents a schematic of the large‐scale setup required for the pyrolysis process. Several SMAs, DMAs, TMAs, and MMAs have been successfully synthesized in the last decade using this process.^[^ ^17^, ^18^, ^85^, ^86^, ^87^, ^88^, ^89^ ^]^ This process generally requires a temperature over 700 °C, making it energy‐intensive and possibly curtailing its industrial utility. Similar to most other methods compatibility between the metal ion and the support is of utmost importance.

## 4 Preparation of SMA/DMA/TMA/MMA Sites Coordinated with Different Species

The high surface energies of transition metals pose a considerable challenge when controlling the configurations of SMAs, DMAs, TMAs, and MMAs coordinated with C, N, P, B, S, and O species. Highly defective 2D materials are ideal platforms for the rational design and preparation of high‐performance SMA/DMA/TMA/MMA‐based electrocatalysts with different coordination environments. Substantial progress has been made in developing easy techniques to construct SMAs, DMAs, TMAs, and MMAs with remarkable properties. This section discusses the methods used and challenges encountered when synthesizing these materials.

### 4.1 Synthesis of SMAs Sites Coordinated with Different Species

**Figure 15:**
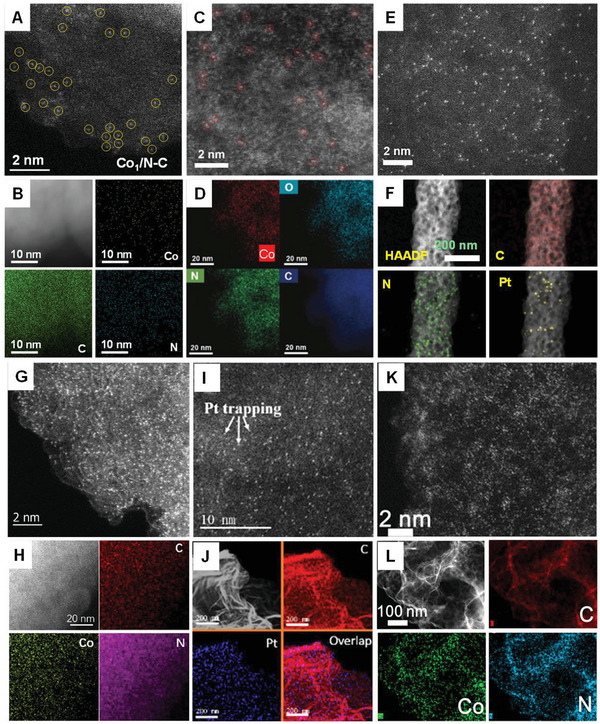
Synthesized SMAs using different techniques. A) Aberration‐corrected high‐angle annular dark‐field scanning transmission electron microscope (AC HAADF‐STEM) image and B) corresponding element mapping of Co_1_/N‐C fabricated via ball milling and pyrolysis. Reproduced with permission.^[^ ^57^ ^]^ Copyright 2023 Elsevier B.V. C) AC HAADF‐STEM image and D) corresponding element mapping of Co–N_5_–O–C fabricated via pyrolysis. Reproduced with permission.^[^ ^114^ ^]^ Copyright 2023 Elsevier B.V. E) AC HAADF‐STEM image and F) corresponding elemental mapping of Pt‐SA/pCNFs fabricated via electrospinning, carbonization, impregnation, and pyrolysis. Reproduced with permission.^[^ ^48^ ^]^ Copyright 2023 Elsevier B.V. G) AC HAADF‐STEM image and H) corresponding element mappings of Co‐SA@NCA fabricated through pre‐crosslinking and pyrolysis. Reproduced with permission.^[^ ^115^ ^]^ Copyright 2020 Elsevier B.V. I) AC HAADF‐STEM image and J) corresponding element mapping of Pt‐single‐atom‐embedded defective graphene (Pt SAs/DG) fabricated using the thermal emitting method. Reproduced with permission.^[^ ^81^ ^]^ Copyright 2019 American Chemical Society. K) AC HAADF‐STEM image and L) corresponding element mapping of Co‐N‐Gr fabricated via pyrolysis and template removal. Reproduced with permission.^[^ ^116^ ^]^ Copyright 2020 American Chemical Society.

The use of SMAs for heterogeneous and electrochemical catalysis has made substantial progress owing to the dispersion of isolated metal atoms, maximized use of these metal atoms, and asymmetrical coordination environment. Recently, significant efforts have been dedicated toward the development of SMAs, and several techniques have been introduced for preparing different types of SMA‐coordinated C, N, P, B, S, and O species (**Figure** **15**). The procedures used for the synthesis of SMAs are discussed below.

The wet synthesis strategy efficiently produces 2D‐material‐supported SMAs on a large scale. This strategy includes impregnation, adsorption, coprecipitation, and strong electrostatic adsorption. Lee et al.^[^ ^90^ ^]^ used an impregnation method to synthesize 0.35 wt.% isolated Pt atoms on TiN. Ma et al.^[^ ^91^ ^]^ developed an impregnation protocol for synthesizing Pt_1_/α‐MoC, in which the amount of single Pt atoms was ≈0.2%. The evenly dispersed Pt_1_ maximized active interfaces in Pt_1_/α‐MoC and drastically enhanced the density of active sites for chemical reactions. Wu et al.^[^ ^92^ ^]^ developed atomically dispersed Ni atoms on N‐doped graphene using a facile ion‐adsorption route, followed by low‐temperature (≈300 °C) annealing in an inert environment to enhance the durability of the product. Ni^2+^ was bonded to four pyridinic N to form single metal atoms (Ni–N_4_) as active sites in the N‐doped graphene. Hutchings et al.^[^ ^93^ ^]^ synthesized a series of 1 wt% Au‐doped carbon catalysts using impregnation and adsorption strategies. Sun et al.^[^ ^94^ ^]^ used the coprecipitation method to fabricate a single Ru embedding on the surface of CoFe‐LDHs (Ru/CoFe‐LDHs). A strong electronic coupling reaction occurred between the Ru and LDH support during the synthesis of the Ru/CoFe‐LDHs at an optimized pH of 12. This method is straightforward and produces large quantities of the product.

A Pd/TiO_2_‐anatase SAC (Pd_1_/TiO_2_) was recently synthesized via the strong electrostatic adsorption process.^[^ ^95^ ^]^ Here, 0.0125 wt% of a single Pd atom was loaded on TiO_2_, as confirmed by inductively coupled plasma analysis. The amount of isolated Pd_1_ (≤ 0.05 wt.% loading) was confirmed via X‐ray absorption spectroscopy.^[^ ^95^ ^]^ Extensive efforts have resulted in significant progress in synthesizing SMAs using various wet synthesis strategies. However, very low metal loading with uncontrollable dispersibility yields unsatisfactory catalytic activity.

Hydrothermal and solvothermal processes can be useful for synthesizing SMAs. Yoon et al.^[^ ^96^ ^]^ used the organic‐template (TEMPO‐oxidized cellulose nanocrystal (TCNC)) hydrothermal technique to synthesize single‐Co‐atom‐doped V_2_O_5_∙nH_2_O nanobelts (CoVO NBs). During the hydrothermal reaction, the negative charges owing to the presence of COOH^−^ and OH^−^ groups in the TCNC attracted the metal ions and aligned them in a one‐dimensional structure. The TCNCs were removed via a thermal treatment, and highly crystalline CoVO NBs were obtained. Lu et al.^[^ ^97^ ^]^ provided another example of using the hydrothermal process for synthesizing SMAs. The author used an acidic environment to fabricate different types of extensively modified single‐atom spin materials in which magnetic atoms (M_1_) were substituted into an MoS_2_ substrate. The main restriction of this method is that isolated metal atoms were not catalytically active for chemical reactions. Zhang et al.^[^ ^98^ ^]^ prepared SMAs (Mo, W, and Nb bonded with O functional groups) on graphene oxide via a one‐pot solvothermal method in the presence of dimethylsulfoxide (DMSO). During the reaction, the dissociative metal ligands (DMSO/MCl_5_ solute; M: Mo, W, or Nb) were implanted into the O functional groups of graphene oxide to form –O*_x_* M species via Lewis acid‐base interactions, forming SMA‐implanted graphene oxide.^[^ ^98^ ^]^ Unfortunately, this method is only applicable for preparing a limited number of metal precursors. Ball milling (mechanochemical synthesis) allows a facile and rapid kilogram‐level synthesis of SMAs.^[^ ^99^, ^100^ ^]^ Yao et al.^[^ ^61^ ^]^ reported high‐energy mechanochemical ball milling for the large‐scale production of isolated individual Ru atoms in a defective MoS_2_ substrate (denoted as Ru_1_/D‐MoS_2_). They found that single Ru atoms induced several S vacancies in MoS_2_, which can destroy charge neutrality around the Ru atoms. This process yielded an asymmetrical electron distribution. Furthermore, the authors’ group reported the large‐scale synthesis of SMAs via ball milling.^[^ ^60^ ^]^ SMA synthesis involves the following steps. Step 1) Vulcan XC‐72R is heated in a tube furnace in an NH_3_ environment to achieve N‐doped carbon (NC). Step 2) A metal precursor and NC are mixed via ball milling, followed by NaBH_4_ reduction.^[^ ^60^ ^]^ The main drawback of this technique is that SMAs are prone to aggregation during ball milling, yielding nanoparticles.

The photoreduction method has been implemented for the preparation of SMAs. Wang et al.^[^ ^62^ ^]^ developed a photochemical strategy with auxiliary H_2_ for anchoring atomically dispersed Cu or Co on a 2D black P (BP) support. This procedure produces stable and high‐loading SMAs (11.3 wt%) with weakly bonded Cu‐P_3_ or Co‐P_3_ species on BP. During the synthesis of Cu and Co single atoms, visible light increases the number of H radicals on the BP layers, which is critical for synthesizing high‐loading metal atoms.^[^ ^62^ ^]^ This procedure does not require special instruments and can, therefore, be easily implemented in laboratories. The catalytically active sites of the materials synthesized via this process are non‐homogeneous owing to the presence of continuously packed sites.

ALD, also known as atomic layer epitaxy, is a versatile approach for preparing SMAs by optimizing the precursor dose time on the supported surfaces. Jang et al.^[^ ^101^ ^]^ reported a NiO/Ni support with atomically dispersed Ir doped using a traveling‐wave‐type ALD reactor. Only one ALD cycle was used for the deposition of isolated Ir atoms using C_18_H_27_IrO_3_ and O_2_ as precursors because ligands or functional groups are needed during ALD; this is the largest obstacle to the use of this process in industries.^[^ ^101^ ^]^

CVD is a powerful technique for producing high‐quality SMAs. Wu et al.^[^ ^71^ ^]^ used a cyclopentadiene‐shielded Fe atom, ferrocene, as a salt precursor to prepare single‐atom Fe sites supported on N/S‐doped carbon sheets via CVD. In this process, S atoms inhibited the formation of nanoparticles, and pyridinic N enhanced the density of active Fe sites.^[^ ^71^ ^]^ The S and N atoms on the carbon surface promoted mass and charge transfers. This method was used to prepare dense active sites and an optimal local environment; however, the production levels were low.

Another approach to synthesizing SMAs is to utilize laser power, which is programmable and controllable in various energy densities. Liu et al.^[^ ^63^ ^]^ used a simple laser‐induced solid‐phase process to synthesize isolated Pt atoms onto a graphene oxide substrate. Picosecond ultraviolet source (UV; 355 nm) and nanosecond infrared source (IR; 1064 nm) Nd:YAG lasers are used for synthesis.^[^ ^63^ ^]^ These lasers are used for a short heating time with a rapid cooling that prevents the migration of metal atoms at the graphene oxide surface. The Pt loading was 0.41wt. % achieved by laser irradiation.^[^ ^63^ ^]^ Li et al.^[^ ^75^ ^]^ used laser ablation for the controllable synthesis of individual Pt atoms in CeO_2_. Laser ablation can be adjusted with high precision to achieve SMAs owing to the super adjustability. The preparation of 100% isolated SMAs is still a challenge because the nucleation and the growth of various metals in the presence of a laser are difficult to prevent.^[^ ^75^ ^]^

The trapping process is a rapid and simple method to synthesize a series of SMAs. Du et al. synthesized Ni atoms stabilized on vanadium carbide (NiSA‐VC) using an atomic trapping strategy.^[^ ^79^ ^]^ As the temperature increased from 700 to 1000 °C, the atomization process was initiated, leading to the conversion of Ni NPs into SAs that are trapped in N‐doped carbon.^[^ ^79^ ^]^ Similarly, Li et al. reported the zeolite imidazolate framework‐8‐derived N‐doped carbon as a substrate to anchor migrating SMAs at 900 °C.^[^ ^102^ ^]^ Zhang et al.^[^ ^48^ ^]^ captured the Pt metal atoms in nitrogen‐doped porous carbon nanofibers by trapping approach. The hydrophilicity and strong microporous capillary forces of nitrogen‐doped carbon help capture platinum precursors.^[^ ^48^ ^]^ At high temperatures, N/C strongly coordinates with platinum atoms, forming platinum metal atoms at the surface of N‐doped carbon.^[^ ^48^ ^]^ The thermal emitting method is an interesting and low‐cost approach to synthesizing SMAs. Li et al. reported that the thermal emitting strategy to prepare single Pt atoms.^[^ ^81^ ^]^ In this process, bulk Pt is used as a precursor. Dicyandiamide (DCD), Pt mesh, and graphene oxidation are placed sequentially in a porcelain boat. This boat is annealed at 1100 °C under inert gas flow. During high‐temperature treatment, the Pt^δ+^ species are trapped by the defective graphene (DG), achieving SMAs on DG.^[^ ^81^ ^]^ The SMAs migrate freely over the defective carbon surface, making it difficult to control the metal–support interactions, resulting in a trapping process that must be improved to control the SMA movement.

Cheng et al.^[^ ^82^ ^]^ prepared a series of SMAs (M: Mn, Fe, Co, Ni, and Pt) into the carbon lattice by a flash bottom‐up arc discharge method. The crystalline carbon lattice is observed during carbon nanosheet formation (nanohorns; CNHs and N‐doped arc graphene flakes; NAG). The Cl atoms in the metal precursor and the nitrogen species in the buffer gases played key roles in stabilizing the SMAs in the carbon lattice to form partial M–N–C moieties.^[^ ^82^ ^]^

Zeng et al.^[^ ^83^ ^]^ reported single‐atom M (M  =  Ir, Ru, Rh, Pd, Ag, Pt, Au, Fe, Co, Ni, Zn, V, Cr, Mn, and Cu) on Co(OH)_2_ nanosheets using an electrochemical deposition method. The SMAs were doped by applying anodic (1.10 to 1.80 V) or cathodic (0.10 V to −0.40 V) deposition with a scan rate of 5 mV s^−1^.^[^ ^83^ ^]^ They also found that the electronic states of the same metal have changed according to the use of cathodically and anodically deposited processes.^[^ ^83^ ^]^

The pyrolysis method is the best approach to synthesize SMAs at an industrial level. Several types of precursors (e.g., polymers, ionic liquids, metal‐organic frameworks (MOFs), graphene, carbon nanotube, metal–macromolecular complexes, and inorganic compounds) are used to fabricate a hybrid before placing it for carbonization under a gas stream of inert/corrosive environment, such as Ar, N_2_, NH_3_, and PH_3_. Xue et al.^[^ ^103^ ^]^ produced the atomically dispersed V atoms embedded in N‐doped carbon nanofibers by selecting the proper combination of polymer, pyrolysis, and leaching processes. Duan et al.^[^ ^104^ ^]^ reported a heterojunction between single atom Co attached to N‐doped carbon sheets and graphitic carbon nitride (g‐C_3_N_4_), which was fabricated by calcinating the hybrid of PBA‐Co (Prussian blue analog‐Co_3_[Co(CN)_6_]_2_) and polymer precursor PEI‐MCA (melamine: M, cyanuric acid: CA, polyethyleneimine: PEI). The heterojunctions promoted electron networking between the SMAs and g‐C_3_N_4_, rapidly transferring g‐C_3_N_4_ to SMAs.^[^ ^104^ ^]^ Jing et al.^[^ ^105^ ^]^ prepared isolated Zn atoms on the g‐C_3_N_4_ surface by polymerization and thermal treatment. They inserted 1.6 wt. % SMAs in the cave of g‐C_3_N_4_ and constructed the atomic Zn(II)‐N5 species through the ionic cation–π interactions (Zn^δ+^‐C_2_^δ−^ type).^[^ ^105^ ^]^ Song et al.^[^ ^106^ ^]^ reported single Cu atoms with unique Cu_1_−N_3_O_1_ moieties using a polymerization and pyrolysis processes. A series of individual Cu atoms with different Cu densities was achieved by controlling the concentration of the Cu precursor.^[^ ^106^ ^]^ The maximum loading of single Cu atoms is 21.3 wt. %, which is equivalent to a Cu density (2.4 atoms/nm^2^).^[^ ^106^ ^]^ Yao et al. reported the synthesis of single Pd atoms via impregnation and subsequent pyrolysis.^[^ ^86^ ^]^ Transform Pd nanoclusters were transformed into single Pd atoms in three steps: Step 1) an organic‐ligand‐shelled [Pd_3_Cl(PPh_2_)_2_(PPh_3_)_3_]^+^[SbF_6_]^−^ (denoted ad Pd_3_Cl) nanoparticle is synthesized as the metal precursor; Step 2) Zeolite imidazolate framework 8 (ZIF‐8) derived ultra‐porous carbon is mixed with Pd_3_Cl clusters; Step 3) The mixture was placed in a tube furnace, carbonized, and pyrolyzed at different temperatures to obtain single Pd atoms. The Pd amount for single Pd atoms is 1.0 wt.%.^[^ ^86^ ^]^

The construction of single Ni atoms from 1‐butyl‐3‐methylimidazolium tetrafluoroborate ([BMIM][BF_4_]) as a liquid nitrogen source is another example of a pyrolysis approach.^[^ ^85^ ^]^ In the fabrication procedure, a carbon support was first obtained by the carbonization of sodium citrate. The carbon support is mixed in [BMIM][BF_4_]‐NiCl_2_, which is then pyrolyzed under the Ar flow to embed the single Ni/N atoms into the carbon sheets to form a Ni–N–C configuration.^[^ ^85^ ^]^

Zhang et al. reported the specific structure of Cu‐S_1_N_3_,^[^ ^107^ ^]^ which was synthesized through two heating steps at different temperatures. First, they prepared the Cu‐N_4_ configuration by forming a powder (mixing NaCl, CuCl_2_·2H_2_O, and glucose) nitrided in NH_3_ gas flow to produce Cu‐N_4_ powder.^[^ ^107^ ^]^ Second, N was replaced by S using a sulfur powder mixed with Cu‐N_4_, pyrolyzed under Ar gas flow, and maintained at 450 and 950 °C.^[^ ^107^ ^]^ Similarly, Mu et al.^[^ ^108^ ^]^ synthesized CoB_1_N_3_ moieties using chitosan, boric acid, and cobalt dichloride as precursors and subsequent pyrolysis under vacuum and H_2_/Ar mixture gases.^[^ ^108^ ^]^

Metal–organic frameworks (MOFs) are porous materials formed by organic ligands and metal centers. These MOFs act as rigid templates to produce C, N, B, P, S, and O atom‐coordinated SMAs.

Chen et al.^[^ ^109^ ^]^ reported the atomically dispersed single Ni atoms within hierarchically porous carbon nanoflowers (Ni‐SA/HPCF), synthesized from aluminum‐based MOF (MIL–101–NH_2_), followed by segmental pyrolysis and acid leaching.^[^ ^109^ ^]^ On the other hand, Liu et al. used a pyrolysis/acid etching method to synthesize Co−S−N active sites implanted on porous carbon.^[^ ^110^ ^]^ The pyrolysis method shows some benefits in terms of high SMAs loading, but it is very difficult to control the loading because of the complex and unknown high temperatures during the chemical reactions.

Most reported strategies have various issues, e.g., loading, controlling reaction, and high temperature. Therefore, other methods have also been introduced to synthesize SMAs over the last two years. For example, SMAs can be constructed by microfluidic,^[^ ^111^ ^]^ ammonium iodide,^[^ ^112^ ^]^ and plasma bombing.^[^ ^113^ ^]^ A synthetic technique based on droplet microfluidics is reported. deMello et al.^[^ ^111^ ^]^ synthesized single metal atoms (Pd and Pt) supported on gC_3_N_4_. This process takes a very short time and combines with the wet impregnation. The synthesis of SMAs can also be realized using ammonium iodide (NH_4_I) etchant method.^[^ ^112^ ^]^ During a high‐temperature treatment in NH_4_I, the metal nanoparticles are converted into volatile MI_x_ and discharged into the gas stream, leading to the formation of the M–N–C motif.^[^ ^112^ ^]^ This method can synthesize a series of SMAs containing precious and non‐precious transition metals.^[^ ^112^ ^]^ Tian et al.^[^ ^113^ ^]^ reported the large‐scale synthesis of single Fe atoms by the plasma bombing. In this technique, plasma bombing excites the metal precursors into SMAs. The SMAs are trapped and embedded via the defective regions of the carbon supports.^[^ ^113^ ^]^

### 4.2 Synthesis of DMA Sites Coordinated with Different Species

**Figure 16:**
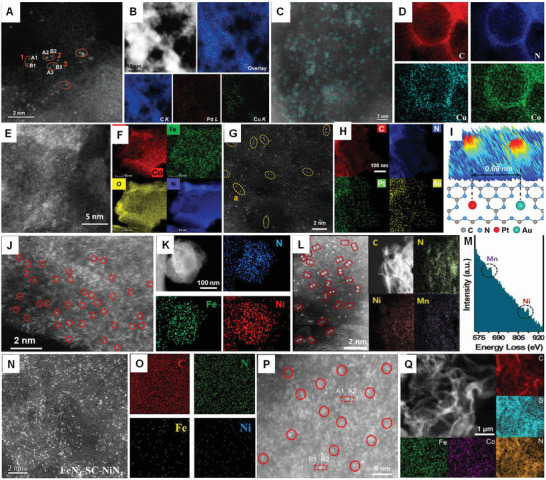
DMAs synthesized using different procedures. AC HAADF‐STEM image (A) and their corresponding element mapping (B) of Pd_1_Cu_1_/ND@G, which was fabricated using strong electrostatic adsorption and deposition−precipitation processes. Pd−Cu atomic pairs are marked with orange ovals; reproduced with permission.^[^ ^121^ ^]^ Copyright 2022 American Chemical Society. AC HAADF‐STEM image (C) and their corresponding element mapping (D) of CuC_4_/CoN_4_@HC, which was prepared by misplaced deposition process. Pd−Cu atomic pairs are marked with orange ovals. Reproduced with permission.^[^ ^129^ ^]^ Copyright 2021 American Chemical Society. AC HAADF‐STEM image (E) and their corresponding element mapping (F) of Co−Fe−N−C, which was fabricated by the pyrolysis process. Reproduced with permission.^[^ ^117^ ^]^ Copyright 2019 American Chemical Society. AC HAADF‐STEM image (G) and their corresponding elemental mapping (H) of Pt‐Au DMAs, which was prepared using an impregnation adsorption strategy. I) Intensity surface plot of the Pt–Au pair. Reproduced with permission.^[^ ^130^ ^]^ Copyright 2022 the Author(s) Published by PNAS. AC HAADF‐STEM image (J) and their corresponding element maps (K) of Ni−Fe−NC, which was fabricated by a calcination process. Reproduced with permission.^[^ ^124^ ^]^ Copyright 2023 American Chemical Society. L) AC HAADF‐STEM image and their corresponding element maps of NiMn DMAs, which were constructed using ion adsorption and pyrolysis processes. M) EELS spectrum of NiMn DMAs. Reproduced with permission.^[^ ^118^ ^]^ Copyright 2022 Elsevier B.V. AC HAADF‐STEM image (N) and their corresponding element mapping (O) of FeN_4_‐SC‐NiN_4_, which was fabricated via adsorption and pyrolysis processes. Reproduced with permission.^[^ ^119^ ^]^ Copyright 2023 Elsevier B.V. AC HAADF‐STEM image (P) and their corresponding element mapping (Q) of FeCo‐NSC, which was fabricated using a template‐directed method. Reproduced with permission.^[^ ^131^ ^]^ Copyright 2021 Elsevier B.V.

Catalysts with DMAs coordinated C, N, P, S, B, and O species incorporated on 2D materials (i.e., metal or metal oxide nanosheets, graphene or graphene‐like sheets) are needed to improve the utilization efficiency of metal electrocatalysts, especially noble metal catalysts. Therefore, the synthetic procedures should be controlled to tune the efficiency of the homo‐ and bi‐nuclear DSAs catalysts and achieve high catalytic stability and activity. Similar to SMAs preparation, DMAs are also synthesized by electrochemical deposition, ALD, ball milling, wet chemical technique, and pyrolysis process (**Figure** **16**). Each technique is reported in Section 3. Each method is discussed based on recent reports. Hu et al.^[^ ^117^ ^]^ reported the synthesis of DMAs (Co–Fe–N–C) using calcination and in situ electrochemical deposition. The single Co atoms bonded to C and N moieties are prepared under the calcination process, while isolated Fe atoms are synthesized using an electrochemical method. During the deposition process, Fe is anchored, and a dimeric Co–Fe species is constructed through one or two linking Ohs.^[^ ^117^ ^]^ Su et al.^[^ ^69^ ^]^ fabricated Pt‐Ru dual‐metal dimers supported on nitrogen‐doped carbon nanotubes (NCNTs) using a two‐step ALD route. In Step 1, single Pt atoms are achieved by ALD, in which the Pt salt precursor is captured on electron‐deficient sp^2^‐nitrogen sites on NCNTs, resulting in strong interactions between the Pt atom and support (N‐doping sites) through chemical bonding.^[^ ^69^ ^]^ In Step 2, excellent‐quality Pt–Ru dimers are obtained by the ALD of Ru on Pt single atoms using Ru salt precursors.^[^ ^69^ ^]^

Ball milling is an efficient and eco‐friendly process for fabricating DMAs in large quantities. Qiang et al.^[^ ^58^ ^]^ used this mechanochemical method to prepare a DMA (Zn–Cr)‐based catalyst for the terpolymerization of CO_2_, propylene oxide, and phthalic anhydride. A certain amount of ZnCl_2_, K_3_Cr(CN)_6_ with a ^t^BuOH is placed into a vessel and ground with steel balls of different sizes at 50 Hz.^[^ ^58^ ^]^ Finally, the powder was washed with a DI water and ^t^BuOH solution to eliminate the unwanted reactants and dried under a vacuum oven.^[^ ^58^ ^]^

A literature survey found that many techniques reported for synthesizing SMAs cannot be applied to preparing DMAs. Therefore, a few techniques are available for fabricating DMAs. In this issue, most reported techniques are pyrolysis/annealing processes with or without a combination of wet chemical approaches. For example, Han et al.^[^ ^118^ ^]^ combined the ion adsorption process and pyrolysis to synthesize the DMAs (N‐coordinated Ni and Mn) anchored in N‐doped carbon. In situ polymerization (polypyrrole) and carbonization (sodium citrate) were used to fabricate the N‐doped carbon.^[^ ^118^ ^]^ Nickel and manganese nitrate were then adsorbed in N‐doped carbon via an adsorption process, followed by pyrolysis under an inert environment.^[^ ^118^ ^]^ Qiu et al.^[^ ^119^ ^]^ reported the synthesis of FeN_4_ and NiN_4_ atomic sites on the S‐doped carbon hollow spheres (denoted as FeN_4_–SC–NiN_4_), which involved an adsorption process (negatively charged SiO^2−^ and positively charged Fe/Ni precursors). The FeN_4_‐SC‐NiN_4_ product was obtained using a heating and leaching process.^[^ ^119^ ^]^ The presence of −NH_2_ functional groups at the L‐Met (L‐Methionine) surface helps in metal ion adsorption through coordination interactions, resulting in the formation of DMAs during pyrolysis.^[^ ^119^ ^]^ Li et al.^[^ ^120^ ^]^ fabricated Co–Fe dual‐metal atoms supported on N/S species hosted in a carbon sphere using the co‐precipitation reaction and pyrolysis/leaching processes, constructing atomically dispersed Co/Fe dual‐atoms. Ma et al.^[^ ^121^ ^]^ reported the preparation of DMAs (Pd_1_Cu_1_/ND@G) using a strong electrostatic adsorption method combined with a deposition and precipitation method. The amount of Cu and Pd atoms were 0.49 and 0.09 wt%, respectively.^[^ ^121^ ^]^ The Cu single atoms (Cu_1_) were first deposited on defective nanodiamond graphene. The Pd single atoms (Pd_1_) were further anchored during the second deposition step. The final sample was obtained by the in‐situ reduction of Pd_1_Cu_1_/ND@G precursor.^[^ ^121^ ^]^

The pyrolysis of MOFs^[^ ^122^, ^123^ ^]^ (controlled structures with high surface areas) has been used extensively to produce DMAs owing to their easy operation and high production rate. Li et al.^[^ ^122^ ^]^ reported the synthesis of N‐coordinated DMAs (Cu–Ni) using porous MOFs. The Cu and Ni precursors, copper acetylacetonate and nickel acetylacetonate, are trapped during MOF crystallization.^[^ ^122^ ^]^ The in situ‐encapsulated Cu^2+^ and Ni^2+^ were then calcined at 950 °C to decompose Cu and Ni precursors and carbonize the organic linkers of the MOF, resulting in Zn evaporation and the subsequent formation of Cu–Ni DMAs into N‐doped carbon.^[^ ^122^ ^]^ Han et al.^[^ ^123^ ^]^ also fabricated Cu‐Ni DMAs using a zeolitic imidazolate framework‐8 (ZIF‐8). The ZIF‐8 was synthesized via the coordination effects of Zn^2+^ and 2‐methylimidazole. Copper nitrate and nickel nitrate were grown on the ZIF‐8 surface using the benzimidazole ligand in ethanol.^[^ ^123^ ^]^ Finally, the resulting products were placed in a tube furnace and pyrolyzed at 900 °C under an inert environment to obtain the Cu‐Ni DMAs.^[^ ^123^ ^]^ Atomically dispersed Ni and Fe DMA sites embedded in N‐doped porous hollow carbon cages were synthesized using an MOF‐carbonized procedure.^[^ ^124^ ^]^

The preparation of DMAs through direct‐pyrolysis methods^[^ ^87^, ^125^, ^126^, ^127^ ^]^ is a favorable process for synthesizing materials on a large scale. In this regard, Liu et al.^[^ ^85^ ^]^ reported the Co–Ru–N_6_ coordination system implanted on N‐doped carbon. This DMA was prepared via a direct pyrolysis process.^[^ ^87^ ^]^ Liu et al. reported the Fe and Co (DMAs) on N‐doped graphene.^[^ ^125^ ^]^ This DMA was achieved using a one‐step pyrolysis method followed by complete mixing.^[^ ^125^ ^]^ Interestingly, Gao et al.^[^ ^126^ ^]^ synthesized Fe‐based DMAs embedded in N‐doped carbon (VFe/NC) using a pyrolysis carbonization procedure. In this synthesis, chitosan was used as the carbon and nitrogen sources, and vanadium (III) chloride and iron (III) chloride were employed as metal precursors.^[^ ^126^ ^]^ During the synthesis of VFe/NC, metal ions are bonded with the ─NH_2_ groups of the chitosan chain via a complexing process.^[^ ^126^ ^]^ Fan et al.^[^ ^127^ ^]^ proposed a polymerization‐pyrolysis strategy for fabricating Fe‐Pt DMAs into N‐doped graphene. In this preparation, iron(III) chloride hexahydrate, dihydrogen hexachloroplatinate (IV) hydrate, glucose, and dicyandiamide are mixed and freeze‐dried to obtain a powder.^[^ ^127^ ^]^ Finally, the pyrolysis process and acid leaching were used to obtain the Fe–Pt DMAs into N‐doped graphene.^[^ ^127^ ^]^ Choi et al.^[^ ^128^ ^]^ reported the synthesis of Sn–Cu (DMAs) anchored on C_3_N_4_ by the co‐pyrolysis of Sn‐ and Cu‐acetylacetonate (metal precursors) and urea under inert gas at 550 °C. The loadings of Sn and Cu atoms were ≈0.99 and ≈0.64 at. %, respectively. Although this process is simple, the main problem with this technique is the difficulty in understanding the synthesis mechanism, which leads to low reproducibility and uncertain active sites.

### 4.3 Synthesis of TMAs and MMAs Sites Coordinated with Different Species

**Figure 17:**
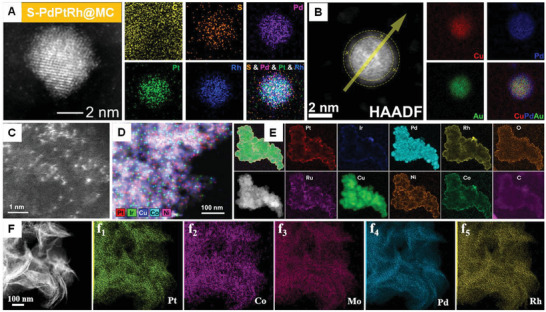
Synthesized TMAs/MMAs using various methods. A) AC HAADF‐STEM image and element mapping of S‐PdPtRh@MC (TMAs) were fabricated by hydrothermal and pyrolysis methods. Reproduced with permission.^[^ ^132^ ^]^ Copyright 2023 American Chemical Society. B) AC HAADF‐STEM image and element mapping of Cu_1_Au_1_@Cu_1_Pd_3_(TMAs) were prepared by annealing. Reproduced with permission.^[^ ^135^ ^]^ Copyright 2023 American Chemical Society. C) AC HAADF‐STEM image of HESAs, which was prepared using a laser‐planting strategy. D) STEM image and element mapping of high‐entropy SAs (HESAs; MMAs). Reproduced with permission.^[^ ^76^ ^]^ Copyright 2023 American Chemical Society. E) EDX‐overlay, AC HAADF‐STEM images, and element mapping of Pt−Ir−Pd−Rh−Ru−Cu−Ni−Co HEAs (MMAs), which were fabricated by hydrothermal and solvothermal processes. Reproduced with permission.^[^ ^143^ ^]^ Copyright 2022 American Chemical Society. F) AC HAADF‐STEM image and element mapping of Pt(Co/Ni)MoPdRh (MMAs) fabricated by wet chemical method. Reproduced with permission.^[^ ^140^ ^]^ Copyright 2023 Elsevier B.V.

The embedding of tri‐metal single atoms into 2D carbon or non‐carbon materials (**Figure** **17**) has attracted considerable attention because of the additional advantages derived from the modulation of the electronic configuration, including maximum metal utilization and unique size quantum effects. Qui et al. designed heterogeneous M^1^N_4_‐C‐M^2^N_4_‐C‐M^3^N_4_ ternary metal single atoms.^[^ ^18^ ^]^ Non‐precious‐metallic CoN_4_‐C‐NiN_4_‐C‐FeN_4_ were fabricated by pyrolysis.^[^ ^18^ ^]^ In this TMA, interbedded Ni liberated electrons for Fe and Co, drastically improving the catalytic activity via a “site‐selective master‐servant” mechanism.^[^ ^18^ ^]^ In 2023, Wang et al. developed a thiol‐assisted strategy to synthesize S‐bonded TMAs on the surface of mesoporous carbon through a simple hydrothermal and subsequent pyrolysis approach.^[^ ^132^ ^]^ In hydrothermal processes, the metal salts coordinated with the hydrophobic thiol ligands were located at the hydrophobic center of spherical micelles.^[^ ^132^ ^]^ Finally, S‐doped TMAs were formed by pyrolysis. Triblock copolymer (Pluronic P123) is the primary S source responsible for S‐bonded TMAs within mesoporous carbon.^[^ ^132^ ^]^ Huang et al. constructed ternary Ni, Co, and Ru atoms on the surface super P (SP) by low‐temperature heating for one hour.^[^ ^133^ ^]^ According to DFT calculation and experimental results, the Ru‐Ni/Co interface improves the water dissociation due to modification of the adsorption‐desorption energy towards the H intermediate and electron transfer from atomic Ru to Ni/Co.^[^ ^133^ ^]^

Jiang et al.^[^ ^134^ ^]^ built Ni_18_Fe_12_Al_70_ on nickel foam (NF) by laser‐direct‐writing, followed by etching, leading to a nanoporous surface formed in the NiFeAl electrode. This technology is used to increase production and synthesize materials at low cost.^[^ ^134^ ^]^ Lu et al.^[^ ^89^ ^]^ reported the fabrication of ultra‐high‐density NiPdPt metal atoms (TMAs) using a two‐step annealing process. They synthesized 15 different metals with a content of 23 wt%. They also reported that the two‐step annealing method in stabilizing the high surface densities of SMAs could be achieved by the selective bonding of metal salts to the carrier.^[^ ^89^ ^]^ Zhang et al.^[^ ^135^ ^]^ synthesized Cu_1_Au_1_@Cu_1_Pd_3_ nanodots on N‐doped graphene sheets in three steps. In the first step, N‐doped graphene sheets were synthesized by mixing sucrose and Zn(NO_3_)_2_ heated at high temperatures, in which ZnO produced from Zn(NO_3_)_2_ works as the porogen/template.^[^ ^135^ ^]^ In the second step, Cu_1_Au_1_@Cu_1_Pd_3_ nanodots are prepared using a solvothermal method. Finally, the Cu_1_Au_1_@Cu_1_Pd_3_ nanodots were easily trapped within the defective sites of graphene sheets because of pores (anchoring sites) in the N‐doped graphene sheets.^[^ ^135^ ^]^

MMAs (Figure 17) have attracted considerable attention in heterogeneous catalysis because of their atomic dispersion, maximized utilization of noble metals, and outstanding catalytic performance. Xin et al. synthesized MMA materials based on eight and 12 elements by dissolution and carbonization processes and displayed catalytic activity toward water oxidation.^[^ ^17^ ^]^ They reported that the coordination motifs changed from O to N by increasing the temperature, which was validated by in situ extended X‐ray absorption fine structure (EXAFS), X‐ray absorption near edge structure (XANES), and high‐resolution‐X‐ray photoelectron spectroscopy (HR‐XPS) analysis. Despite having high OER activity based on 12‐metal MMAs, it is not up to industrial standards because of its high overpotential (≈250 mV vs RHE) at 10 mA cm^−2^.^[^ ^17^ ^]^ Furthermore, MMAs^[^ ^136^, ^137^, ^138^ ^]^ in the form of high‐entropy alloys (HEAs) are a new type of alloy, which has more than three elements in disordered structures.

Various applications of HEAs have been reported.^[^ ^138^, ^139^, ^140^, ^141^, ^142^, ^143^, ^144^, ^145^, ^146^, ^147^, ^148^, ^149^, ^150^, ^151^, ^152^, ^153^, ^154^, ^155^, ^156^, ^157^, ^158^, ^159^, ^160^, ^161^, ^162^, ^163^, ^164^, ^165^, ^166^, ^167^, ^168^, ^169^, ^170^ ^]^ Therefore, this paper briefly discusses the preparation for HEAs. Wang et al.^[^ ^140^ ^]^ developed a wet chemical process to fabricate PtCoMoPdRh/PtNiMoPdRh HEAs nanoflowers (NFs), which was compiled via the ultra‐thin nanosheets (thickness of nanosheets of 1.68 nm). In this synthesis, the same molarity of metal acetylacetonate precursors (Pt, Co/Ni, Mo, Pd, and Rh) were dispersed in triethyleneglycol (TEG) in the presence of the surfactant, triethyl benzyl ammonium chloride (TEBA).^[^ ^140^ ^]^ Subsequently, after a few hours of mixing, a d‐(+)‐glucose‐TEG solution and molybdenum hexacarbonyl (Mo(CO)_6_) were poured into the above homogeneously dispersed solution, resulting in the formation of NFs by glucose reduction and TEBA modification.^[^ ^140^ ^]^

HEA materials were also synthesized using pulsed xenon light. Li et al.^[^ ^141^ ^]^ reported the synthesis of high entropy oxides (HEO) materials on carbon fiber paper by a computer‐programmed Xenon flash lamp.^[^ ^141^ ^]^ This Xenon flash lamp can deliver intense pulsed light for photon flash synthesis (PFS). In this method, premixed metal salts are drop‐cast in carbon fiber paper, followed by drying in an oven.^[^ ^141^ ^]^ After being well dried, the sample was irradiated with photonic pulses under N_2_ gas.^[^ ^141^ ^]^ Metal salts were converted to metal nanoparticles owing to substrate heating by adsorbing strong pulsed light. More importantly, this technique is simple and rapid, and HEAs materials could be produced on an industrial scale.^[^ ^141^ ^]^ Zou et al. synthesized HEAs (Pt, Ir, Cu, Ni, and Co) on carbon black through the laser planting process.^[^ ^76^ ^]^ During the synthesis process, the laser pulses produce defects in the substrate and reduce metal salts in MMAs.^[^ ^76^ ^]^ On the other hand, this approach presents several problems in the synthesis conditions (such as temperature and reducing agents).^[^ ^76^ ^]^ Wang et al.^[^ ^142^ ^]^ deposited FeCoNiCuMn HEA nanoparticles evenly onto carbon nanofibers (CNFs) in a CVD furnace. In this fabrication, FeCoNiCuMn/PAN/DICY nanofiber membrane was prepared by electrospinning.^[^ ^142^ ^]^ The membrane was cut to 2 cm × 3 cm in size and placed in a ceramic boat in the middle part of the furnace.^[^ ^142^ ^]^ Finally, the FeCoNiCuMn HEA/CNFs product was obtained by heating at two different temperatures.^[^ ^142^ ^]^ Iversen et al.^[^ ^143^ ^]^ introduced the facile solvothermal method to prepare Pt–Ir–Pd–Rh–Ru–Cu–Ni–Co HEAs. This method needs autoclaves heated to a low temperature (170, 200, and 230 °C). Huang et al. reported the synthesis of FeCoNiRu HEAs from MOF precursor.^[^ ^144^ ^]^ The MOF precursor was fabricated using a solvothermal method. In addition, FeCoNiRu HEAs on carbon skeleton were achieved using a pyrolysis process.^[^ ^144^ ^]^ During the heating process, MOF provided a porous structure, mitigated the growth of nanoclusters, and prevented their aggregation.^[^ ^144^ ^]^ The vacuum system assisted, and the tandem thermal decomposition was also utilized to synthesize HEAs nanoparticles.^[^ ^145^, ^146^ ^]^ The last few years have witnessed the development of approaches for constructing MMAs. These approaches include wet chemistry, CVD, laser process, solvothermal methods, and pyrolysis process. On the other hand, these approaches normally have special conditions for the anchored metals or the supports. Therefore, a new method for synthesizing MMAs is needed.

## 5 Characterization and Interpretation of SMA/DMA/TMA/MMA Sites Coordinated with Different Species

Structure identification is a key point in probing the active sites of SMA/DMA/TMA/MMA catalysts. Advanced characterization methods are essential for the rapid development of atomic site catalysts. Aberration‐corrected high‐angle annular dark‐field‐scanning transmission electron microscopy (AC‐HAADF‐STEM),^[^ ^17^, ^147^ ^]^ differential phase contrast (DPC‐STEM),^[^ ^148^ ^]^ electron energy loss spectroscopy (EELS),^[^ ^149^ ^]^ and scanning tunneling microscopy (STM),^[^ ^150^ ^]^ were used to observe the SMAs/DMAs/TMAs/MMAs directly on a 2D substrate. Combining X‐ray absorption spectroscopy (XAS) and DFT calculation provides reliable information about the local coordination numbers.^[^ ^17^, ^151^ ^]^ The XAS and DFT results also shed light on the coordination structures of catalytically active centers, making it one of the most powerful techniques for atomic catalytic research.^[^ ^17^, ^151^ ^]^ Atom probe tomography (APT)^[^ ^152^, ^153^ ^]^ displayed chemical composition in the form of atomic‐level three‐dimensional (3D) images, becoming another strong tool for observing real active sites. In situ studies have been carried out to monitor the change in chemical reactions discussed in this section, along with the abovementioned techniques.^[^ ^154^ ^]^ Lastly, the main problem of identifying active sites is highlighted.

### 5.1 Physical and Chemical Analysis of SMA/DMA/TMA/MMA Sites Coordinated with Different Species

Many physical and chemical characterizations have been used to examine the presence of single, double, triple, and multiple metal atoms at the surface of 2D materials, which is another significant part of SMA/DMA/TMA/MMA materials. These tools are divided into two groups. The first group includes high‐resolution STM, AC‐HAADF‐STEM, atomic force microscopy,^[^ ^155^ ^]^ and APT, which provide information on atomic‐scale structural details. The second group consists of XAS, Fourier‐transform infrared spectroscopy (FTIR), XPS, TEM‐energy dispersive X‐ray spectroscopy (EDX), EELS, and surface‐enhanced Raman spectroscopy (SERS), which are offered supporting evidence for the existence of single to multiple atoms (**Table** **2**). This section discusses the recent use of these techniques.

**Table 2:** Various techniques for in situ analysis of SMA/DMA/TMA/MMA‐based materials.

Tools	Studies	Physical and chemical properties	Pros and cons	Resolution [nm]
RS and SERS^a)^	Ex situ and in situ test	Chemical bonding and function groups	Atomic‐scale sensitivity; feasibility of in situ tests for the adsorption process; samples difficult to prepare; selective elements analyzed	[0.1]
FTIR^b)^	Ex situ and in situ test	Molecular groups and chemical bonding	Atomic‐scale sensitivity; feasibility of in situ tests for the adsorption process; samples easy to prepare; selective molecules probed	[0.1]
HR‐XPS^c)^	Ex situ and in situ test	Element and oxidation state analysis	Atomic‐scale resolution; probe detailed electronic structure and geometric information; feasibility of in situ tests; the information is not convincing	[0.1]
XAS^d)^	Ex situ and in situ test	Oxidation state, electronic configuration and coordination number	Atomic‐scale sensitivity; feasibility of in situ tests for the adsorption process; sensitive to functional/chemical groups, bonding geometry; local coordination environment; testing time is short and small damage to the material; complicated data processing; difficult to proof presence of single metal atoms; metal species is not determined directly; limited instrument accessibility	[0.1]
STM^e)^	Ex situ and in situ test	Topology and atomic distribution	Atomic‐scale sensitivity; feasibility of in situ tests for the adsorption process; results affected by environment; the test time is very long; sample preparation is difficult; simulations required for STM data analysis; taking too much time	[0.01]
STEM	Ex situ and in situ test	Topology and atomic distribution	Atomic‐scale sensitivity; feasibility of in situ tests for the adsorption process; thick and low contrast specimens can be monitored; micro diffraction can be realized; imaging is limited to small area	[0.08]
EDX^f)^	Ex situ and in situ test	Element analysis	Atomic‐scale sensitivity; feasibility of *in‐situ* tests for the adsorption process; low energy resolution	[0.1]
EELS^g)^	Ex situ and in situ test	Element and chemical state analysis	Atomic‐scale sensitivity; feasibility of *in‐situ* tests for the adsorption process; high‐sensitivity to electronic structure; insensitive to more number metal single atoms	[0.1]
MS^h)^	Ex situ and in situ test	Structure and chemical analysis	Atomic‐scale sensitivity; feasibility of in‐situ tests; probing intermediates; analyzed final products; complex processing	[<0.1]
ICP‐OES^i)^	Ex situ and in situ test	Element analysis	Atomic‐scale sensitivity; feasibility of in‐situ tests; simple data processing; specific elemental analysis; poor precision	[±0.1]

^a)^ RS, Raman spectroscopy; SERS, Surface‐enhanced Raman spectroscopy;

^b)^ FTIR, Fourier‐transform infrared spectroscopy;

^c)^ HR‐XPS, High‐resolution X‐ray photoemission spectrum;

^d)^ XAS, X‐ray absorption spectroscopy;

^e)^ STM, Scanning tunneling microscopy;

^f)^ EDX, Energy dispersive X‐ray;

^g)^ EELS, Electron energy loss spectroscopy;

^h)^ MS, Mass spectrometry;

^i)^ ICP‐OES, Inductively coupled plasma‐optical emission spectrometry.

#### 5.1.1 High‐Resolution Transmission Electron Microscopy (HR‐TEM)

High‐resolution transmission electron microscopy (HRTEM) is used for nanoscale morphologies. On the other hand, this technique cannot examine the configurations of SMAs/DMAs/TMAs/MMAs and their interactions with the support. In contrast, to directly validate the presence of SMAs/DMAs/TMAs/MMAs on the 2D surface, the scanning tunneling microscopy (STM) and the AC‐HAADF‐STEM techniques precisely detect and deliver direct configuration of the SMAs/DMAs/TMAs/MMAs on supports.

#### 5.1.2 Scanning Tunneling Microscopy (STM)

**Figure 18:**
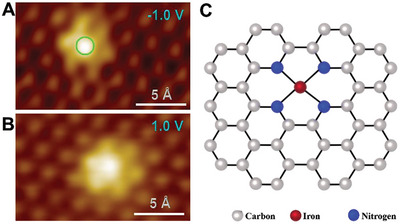
A,B) Atomic resolution scanning tunneling microscopy (STM) images of the Fe–N–C at different bias functions. C) Structure of the FeN_4_ moiety anchored in the graphene sheets.^[^ ^156^ ^]^ Reproduced with permission.^[^ ^156^ ^]^ Copyright 2017 Elsevier B.V.

STM allows researchers to determine the electronic structures of a material surface at the atomic level. The principle of the setup is based on a quantum mechanical phenomenon called tunneling, in which wavefunctions penetrate through a potential energy barrier. This system works in constant‐height and constant‐current modes. The constant‐height mode applies to the smooth surface, and the probe tip scans the sample surface at a fixed *z*‐position. On the other hand, constant‐current mode is used mainly by researchers and operates on irregular surfaces. The tunneling current is kept constant by a feedback circuit that regulates the gap between the tip and surface of the sample. Owing to the lateral resolution of 0.1 nm, it is applicable for examining the SMAs. For example, Bao et al.^[^ ^156^ ^]^ utilized ultra‐high vacuum (UHV) STM to analyze the distribution of FeN_4_ moieties embedded in the graphene sheets. The bright contrast spots in STM images show the Fe atoms (**Figure** **18**A,B). They also reported that the height of these bright dots did not change, suggesting that the electronic configuration of the dots is strongly hybridized with that of the graphene sheets. DFT calculations showed that a Fe–N–C structure was present in the graphene sheets (Figure 18C). Bao et al.^[^ ^150^ ^]^ also reported the embedded Cu(I)–N active sites within graphene sheets. The combination of experimental and theory revealed the successful formation of Cu–N sites embedded in graphene sheets. Ho et al.^[^ ^157^ ^]^ applied inelastic electron tunneling spectroscopy (IETS) with the STM to examine the molecular structure. In these experiments, CO molecules are transferred to the probe tip, and the vibration mode detects the bonding between two atoms.

Based on the above discussion, the combined STM and DFT theory provided morphological and geometrical information. On the other hand, this system is sensitive to atomic contaminants and has difficulty analyzing samples with high surface corrugations. Therefore, STM could be updated to an advanced level that works on any surface that can sense DMA/TMA/MMA structures. Second, in situ STM that can observe SMA/DMA/TMA/MMA structures directly under the reaction environments is needed.

#### 5.1.3 Aberration‐Corrected High‐Angle Annular Dark‐Field‐Scanning Transmission Electron Microscopy (AC HAADF‐STEM)

AC HAADF‐STEM is a method to observe the surface morphologies of SMA/DMA/TMA/MMA‐based materials. Over the last decades, this technique has been used widely for the atomic‐level analysis of SMA/DMA/TMA/MMA‐based materials. A tiny, strong white dot in the image shows the real atoms because more electrons are diffracted from elements with the highest atomic number, resulting in bright dots in the AC HAADF‐STEM images. For deeper analysis of structures, AC HAADF‐STEM should be well combined with other spectroscopic techniques, such as XAS, XPS, and IR.

#### 5.1.4 Energy Dispersive X‐Ray Spectroscopy (EDX)

The 0.1 nm resolution obtained by TEM‐EDS analysis can be used to confirm the presence of SMA/DMA/TMA/MMA on a 2D substrate. During the analysis, the surface area and the shape of the focused electronic beam must be considered, which alters the detection resolution. In addition, a higher peak/background ratio (P/B) showed that SMAs/DMAs/TMAs/MMAs could be recognized by TEM‐EDS. The STEM‐EDS mapping images also exhibited the spatial distribution of the elements. EDS is used to detect metal atoms in hybrids, and STEM can check whether they are single atoms.

#### 5.1.5 Electron Energy Loss Spectroscopy (EELS)

EELS spectrometry provides chemical/elemental information on the picometer scale of SMAs/DMAs/TMAs/MMAs on 2D materials surface.^[^ ^158^ ^]^ In this method, the energy loss occurs due to inelastic interactions between electrons and metal atoms. The changes in kinetic energy of electrons are examined as they pass through a specimen. The technique can be coupled to TEM or STEM because the electrons pass through a thin sample. In analyzing samples, the energy loss of the SMA/DMA/TMA/MMA state differs from that of bulk chemical states. Therefore, the presence of metal atoms can be detected.

#### 5.1.6 Atom Probe Tomography (APT)

**Figure 19:**
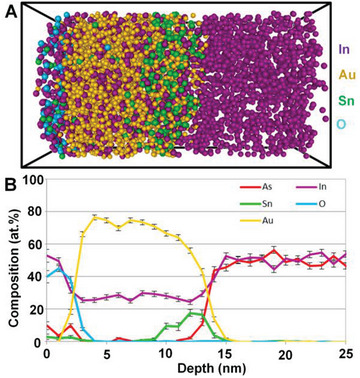
A) APT atom mapping of the In–Au–Sn–O contains nanowires. B) APT composition profile.^[^ ^159^ ^]^ Reproduced with permission.^[^ ^159^ ^]^ Copyright 2019 American Chemical Society.

APT is the only analytical tool that can offer extensive capabilities for 3D imaging and composition of materials with atomic resolution (0.1–0.3nm resolution in depth and 0.3–0.5nm laterally) and parts‐per‐million (ppm) level elemental sensitivity. APT analyses involve applying a large electrical field to a needle‐shaped specimen tip that ionizes, making APT a promising method to verify local compositions. Giddings et al.^[^ ^159^ ^]^ used APT to investigate the In/Sn/Au/O concentrations at the atomic level. The APT mappings and their profile reveal the existence of In/Sn/Au/O atoms at the tip of the nanowires (**Figure** **19**A,B). Heteroatoms, such as B and P distributed in silicon nanocrystals (Si NCs) implanted in borosilicate glass (BSG), phosphosilicate glass (PSG), and borophosphosilicate glass (BPSG), were analyzed by APT.^[^ ^153^ ^]^ This analysis suggested that heteroatoms can be mapped by APT. Based on the insights gained from the above discussion, APT reveals some support for characterizing materials. On the other hand, when the materials are reduced to the atomic scale, this APT cannot distinguish the bond between the metal‐to‐metal or metal‐to‐heteroatoms. Therefore, it is necessary to modify and design an in situ process in this tool to overcome the aforementioned bottlenecks.

#### 5.1.7 X‐Ray Photoelectron Spectroscopy (XPS)

X‐ray photoelectron spectroscopy (XPS) is used to examine the chemical compositions at the surface of materials (top 0–10 nm) and provides the oxidation states of different elements. This is based on striking X‐rays (1–2 keV) on a specimen, and the kinetic energy of photoelectrons emitted by the surface‐metal atoms is measured. XPS can be upgraded and used for in situ studies of surface materials that can reveal the chemical reaction mechanism. This technique can study the characteristics of SMA/DMA/TMA/MMA, providing evidence of different metal atoms.^[^ ^160^, ^161^, ^162^ ^]^

#### 5.1.8 Surface‐Enhanced Raman Spectroscopy (SERS)

Raman spectroscopy examines the relationship between the metal atoms and substrate materials.^[^ ^164^ ^]^ This technique is based on the scattering spectra of different impinged light‐wave frequencies to acquire the rotational and vibrational spectral data of the molecules identified. In addition, the method is also used to examine the molecular structure.^[^ ^165^ ^]^ A highly surface‐sensitive technique called SERS further enhances Raman scattering by single molecules.^[^ ^165^ ^]^ The Raman shifts of atoms in the SERS analysis were used to detect metal atoms in a 2D substrate.^[^ ^165^ ^]^ On the other hand, SERS is applied to a limited number of metal atoms because the enhanced Raman effect only exists in restricted metals (e.g., silver, gold, and copper).

#### 5.1.9 Fourier‐Transform Infrared Spectroscopy (FTIR)

FTIR spectroscopy^[^ ^166^, ^167^ ^]^ is based on the interactions of the materials with irradiated infrared light. This analytical tool is used to obtain molecular structural information that appears in spectral signatures. The active sites of the materials are probed using vibration information^[^ ^166^, ^167^ ^]^ because SMAs/DMAs/TMAs/MMAs exhibit different absorption modes from the nanoclusters. Thus, the evidence obtained from FTIR spectroscopy can be used to analyze the presence of metal atoms on the surface of 2D materials.^[^ ^166^, ^167^ ^]^ IR spectroscopy is classified into several modes, such as transmission IR (TIR), diffuse reflectance infrared Fourier transform spectroscopy (DRIFTS), attenuated total reflection IR (ATR‐IR), and reflection−absorption IR (RAIR). In situ FTIR (referred to as DRIFTS) is a very powerful instrument for determining the presence of metal atoms.^[^ ^167^ ^]^ On the other hand, there are a limited number of probe molecules for in situ FTIR analysis, making it difficult to investigate the behaviors of metal atoms under different reaction conditions.

#### 5.1.10 X‐ray Absorption Spectroscopy (XAS) and Theoretical Model Analysis

Among the various surface analysis tools available, XAS provides deeper information on the distribution of the metal particles or even SMAs/DMAs/TMAs/MMAs, the coordination motif, the binding modes, and the oxidation states of the metal atoms.^[^ ^168^ ^]^ This technique is divided into XANES and extended X‐ray absorption fine structure (EXAFS). XANES analysis obtains the near absorption edge, which ranges from −50 to +200 eV relative to the edge. The XANES spectra provide information on the electronic configuration, oxidation states, and coordination structure (e.g., tetrahedral and octahedral coordination). On the other hand, this technique has limited information because the sample is analyzed under low energy. This problem is overcome using high‐energy resolution fluorescence detection XANES (HERFD‐XANES) spectroscopy. This technique provides the same data with a higher energy resolution and detection sensitivity. EXAFS is also a strong tool for metal atom analysis to identify the coordination numbers, radial distances, and local coordination motifs of the active sites. More importantly, metal–metal bonds are not displayed by the EXAFS spectra of SMAs/DMAs/TMAs/MMAs.^[^ ^158^ ^]^ A spectrum of metal–metal bonds would indicate the presence of nanoparticles or clusters. In situ XANES and EXAFS analyses with machine learning are also available, which help determine the reaction mechanism in real‐time experiments and develop the activity descriptors.^[^ ^169^ ^]^ This technique provides more accurate information on SMAs/DMAs/TMAs/MMAs when combined with XAS, HAADF‐STEM, and DRIFTS. The theoretical model was matched with the XANES/EXAFS spectra, which provide detailed information on the molecular structures. This model was used further for theoretical DFT calculations to measure the binding energy and Gibbs free energy and correlated with the experimental results.^[^ ^170^ ^]^ These results were used to understand the fundamental reaction mechanism that occurred by SMAs/DMAs/TMAs/MMAs.

#### 5.1.11 Other Methods

In addition to the techniques discussed above, there are some other reported methods for analyzing SMAs/DMAs/TMAs/MMAs. For example, solid‐state magic‐angle spinning (MAS) NMR,^[^ ^171^ ^]^ Mössbauer spectroscopy,^[^ ^172^ ^]^ and fluorescence spectroscopy^[^ ^173^ ^]^ provide information on the metal species, local coordination motif, oxidation states, and electronic structures of metal atoms.

### 5.2 Problems in Interpreting Active Sites of SMA/DMA/TMA/MMA Sites Coordinated with Different Species

Many types of direct and indirect techniques have been introduced. Among them, AC HAADF‐STEM and STM are promising tools for confirming the SMAs/DMAs/TMAs/MMAs active sites at the surface of 2D materials. Nevertheless, these techniques cannot probe metal‐to‐carbon and metal‐to‐heteroatom bonding. Furthermore, these tools are useful when testing SMAs but provide incorrect information when characterizing and analyzing DMAs, TMAs, and MMAs on a 2D substrate. On the other hand, EXAFS analysis and the DFT model provided hints of metal‐to‐carbon and metal‐to‐heteroatom structures. Moreover, the main issue with the EXAFS and DFT approach is determining the accurate coordination number. Furthermore, the radial distance between metal atoms and C, N, O, B, and S atoms closely coincide, making it difficult to obtain the precise configuration because of the overlap of different DFT models with experimentally obtained EXAFS spectra. Therefore, there is considerable disagreement about identifying di‐, tri‐, and multi‐metallic sites in SMAs/DMAs/TMAs/MMAs catalysts. Overall, current characterization methods still limit the deep understanding of the bonding between metal atoms and C, N, P, S, O, and B species, limiting the development of various atomic site catalysts. Therefore, new techniques or approaches are needed to understand the coordination environment, which helps identify the true catalytic sites.

## 6 Applications of SMA/DMA/TMA/MMA Implanted 2D Materials

Carbon‐ and non‐carbon‐based SMAs/DMAs/TMAs/MMAs with N, S, P, B, and O species modulated the electronic structure of single‐, di‐, tri‐, and multi‐atom sites, which exhibit excellent catalytic and detecting properties. Therefore, these catalysts are explored in energy‐, sensors‐, and biomedical‐related fields, such as chemical sensors, gas sensors, biosensors, enzymatic reaction, producing H_2_O_2_, ORR, HER, OER, NRR, CO_2_RR, water splitting, fuel cells, and ZABs. This section presents the recent progress in the above‐mentioned applications of SMAs/DMAs/TMAs/MMAs coordinated with N, S, P, B, and O species.

### 6.1 Hydrogen Evolution Reaction

The HER plays a key role in renewable and sustainable hydrogen energy (mass‐energy density: 120 or 142 MJ kg^−1^)^[^ ^174^ ^]^ generation through neutral (potassium phosphate buffer), acid, and alkaline water splitting.^[^ ^170^, ^175^, ^176^ ^]^ More details of the reaction processes with rate‐determining configuration are reported elsewhere.^[^ ^167^, ^168^, ^169^, ^170^, ^171^, ^172^, ^173^, ^174^, ^175^, ^176^, ^177^, ^178^, ^179^ ^]^ Generally, the following elementary steps are involved at the cathode during the water splitting in different pH electrolytes:

(1) $$2H^{+}+2e^{-}\to H_{2},\;E^{\circ }=0.0\;V\left(\mathrm{Acidic}\;\mathrm{medium}\right)$$

(2) $$\begin{array}{ccc} & & 2H_{2}O+2e^{-}\to H_{2}+2OH^{-},\;E^{\circ }=-0.828\;V\left(\mathrm{Basic}\;\mathrm{medium}\right) \end{array}$$

(3) $$\begin{array}{c} 2H_{2}O+2e^{-}\to H_{2}+2OH^{-},\;E^{\circ }=-0.413\;V\;\left(\mathrm{Neutral}\;\mathrm{medium}\right)\;\;\;\;\; \end{array}$$

**Tables** **3**, **4**, **5** list the overpotential (η), Tafel slope, and stability of previously reported SMA/DMA/TMA/MMA‐based materials in acidic, basic, and neutral media, suggesting that the optimal SMA/DMA/TMA/MMA content needs to be optimized to achieve a low potential for hydrogen generation. Therefore, noble‐metal‐based materials have attracted attention owing to their benchmark catalytic activity and stability.

**Table 3:** HER performance of the SMA/DMA/TMA/MMA catalysts in an acidic medium.

Materials	Electrolyte	Overpotential @ 10 mA cm^−2^	Tafel slope [mV dec^−1^]	Stability	Reference
Os/CNS (SMAs)	0.5 m H_2_SO_4_	22	41	60 h	[180]
Pt@DG (SMAs)	0.5 m H_2_SO_4_	30	72	8.3 h	[78]
Pt@WS_2_ (SMAs)	0.5 m H_2_SO_4_	32	28	–	[181]
C‐Co‐ZIFs (SMAs)	0.5 m H_2_SO_4_	322	–	12 h	[182]
(NiPt)‐N_4_C_2_ (DMAs)	0.5 m H_2_SO_4_	30	27	27 h	[183]
Pt–Ru dimers (DMAs)	0.5 m H_2_SO_4_	18	28.9	5000 cycles	[69]
Ni‐doped W_2_C NSs (DMAs)	0.5 m H_2_SO_4_	57	39	28 h	[184]
PANI@Pt/S‐TiN NTs/Ti (DMAs)	0.5 m H_2_SO_4_	12	26.9	25 h	[185]
Co‐NCNTs/MoS_2_ (DMAs)	0.5 m H_2_SO_4_	130	96	28 h	[186]
Co–O–Ti (DMAs)	0.5 m H_2_SO_4_	41	59	50 h	[187]
IrCo_4.9_/NC (DMAs)	0.5 m H_2_SO_4_	14	35	10 000 cycles	[176]
CoP@CoP@(Co/Ni)_2_P (DMAs)	0.5 m H_2_SO_4_	139	70.83	–	[188]
V_0.8_Mo_0.2_Se_2−x_ (TMAs)	0.5 m H_2_SO_4_	67.2	51	60 h	[189]
(MoWReMnCr)S_2_ (MMAs)	0.5 m H_2_SO_4_	229	111.6	20 h	[146]
HEA‐NPs‐14/CNTs (MMAs)	0.5 m H_2_SO_4_	11	27.2	400 h	[190]
ZnNiCoIrMn (MMAs)	0.1 m HClO_4_	50@mA cm^−2^	30.6	100 h	[145]

**Table 4:** HER performances of the SMA/DMA/TMA/MMA catalysts in an alkaline solution.

Materials	Electrolyte	Overpotential @ 10 mA cm^−2^	Tafel slope [mV dec^−1^]	Stability	Reference
Pt_SA_‐NiO/Ni	1 m KOH	26	27	30	[191]
Pt@DG (SMAs)	1 m KOH	37	156	8.3	[78]
Pt_SA_‐NiCo LDH (SMAs)	1 m KOH	≈10	50.2	50	[192]
NiTPP(SMAs)	1 m KOH	138	83	60	[193]
Co@CN_x_ (SMAs)	1 m KOH	270	126	NA	[194]
Fe/SAs@Mo‐based‐HNSs	1 m KOH	38.5	35.6	600	[195]
Ni‐doped W_2_C NSs (DMAs)	1 m KOH	81	87	28	[184]
PANI@Pt/S‐TiN NTs/Ti (DMAs)	1 m KOH	25	31.2	25 h	[185]
Co‐NCNTs/MoS_2_ (DMAs)	1 m KOH	58	68	38	[186]
*β*‐Ni(OH)_2_/Ni‐Ru SAs (DMAs)	1 m KOH	16	21	250	[196]
Co–O–Ti (DMAs)	1 m KOH	29	46	50 h	[187]
Ce‐CoP (DMAs)	1 m KOH	81	68.7	25 h	[197]
CoP@CoP@(Co/Ni)_2_P (DMAs)	1 m KOH	147	72.7	30 h	[188]
V_0.8_Mo_0.2_Se_2−x_ (TMAs)	1 m KOH	72.3	71	60 h	[189]
Ni_18_Fe_12_Al_70_ (TMAs)	1 m KOH	31	37	100 h	[134]
Zn‐Fe/Mn@Mn‐FeP (TMAs)	1 m KOH	53	20.4	80 h	[198]
c‐PtTe_2_ (TMAs)	1 m KOH	14	24.5	40 000 cycles	[199]
NiCoRu_0.3_/supra‐P (SP) (TMAs)	1 m KOH	59	53	100 h	[133]
CeRuSi‐EK (TMAs)	1 m KOH	28	24	10 h	[200]
Co_6_Mo_6_C_2_|NC (MMAs)	1 m KOH	114	58	60 h	[201]
PtCoMoPdRh NFs (MMAs)	1 m KOH	16.5	36.8	8 h	[140]
PtIrCuNiCo/CB (MMAs)	1 m KOH	22	NA	90 h	[76]
FeCoNiCuMn/CNFs (MMAs)	1 m KOH	∼210	53	20 h	[142]
FeCoNiRu (MMAs)	1 m KOH	40	84	40 h	[144]
HEA‐NPs‐14/CNTs (MMAs)	1 m KOH	18	30.7	264 h	[190]
Mo(NiFeCo)_4_/Ni (MMAs)	1 m KOH	47@100 mA cm^−2^	35	500 h	[138]
V_1.0_CuCoNiFeMn (MMAs)	1 m KOH	250@50 mA cm^−2^	148	20 h	[202]
Pt_4_FeCoCuNi (MMAs)	1 m KOH	20	31	50 h	[203]

**Table 5:** Overpotential (η) and Tafel slope of SMA/DMA/TMA/MMA‐based materials reported recently in a neutral medium, indicating that TMA/MMA‐based materials for the HER have not been reported. Therefore, work on the novel synthesis of TMAs/MMAs and their HER activity in PBS is urgent.

Materials	Solution	Overpotential @ 10 mA cm^−2^	Tafel slope [mV dec^−1^]	Reference
Pt_SA_‐NiO/Ni (SMAs)	1 m PBS	27	32	[191]
NiRu_0.13_‐BDC (SMAs)	1 m PBS	36	32	[204]
Co–Mo0.4‐S (SMAs)	1 m PBS	213	94	[205]
MoP (SMAs)	1 m PBS	196	79	[206]
Ni‐doped W_2_C NSs (DMAs)	1 m PBS	63	51	[184]
PANI@Pt/S‐TiN NTs/Ti (DMAs)	1 m PBS	39	37.7	[185]
Co‐NCNTs/MoS_2_ (DMAs)	1 m PBS	84	96	[186]
*β*‐Ni(OH)_2_/Ni‐Ru (DMAs)	1 m PBS	10	21	[196]
Co–O–Ti (DMAs)	1 m PBS	76	71	[187]
CoP@CoP@(Co/Ni)_2_P	1 m PBS	144	108.6	[188]
V_0.8_Mo_0.2_Se_2−x_ (TMAs)	1 m PBS	122.3	66	[189]

The transition metal‐based materials were reviewed for their HER activity in acidic, basic, and neutral electrolytes. In the case of SMAs, Cheng et al.^[^ ^180^ ^]^ examined the effects of coordination environments on the hydrogen production activity. They modified the oxidation states (+0.9 to +2.9) of osmium (Os) metal atoms by changing the coordination motifs (Os‐N_3_S_1_, Os‐N_4_, Os‐S_6_, Os‐C_3_, and Os‐C_4_S_2_).^[^ ^180^ ^]^ In acidic electrolytes, the ideal oxidation state of +1.3 (Os‐N_3_S_1_) showed the greatest HER activity because of the modification of the energy level of the d‐band center, which affected the adsorption of atomic hydrogen and the formation of molecular hydrogen.^[^ ^180^ ^]^ On the other hand, the overpotential of the optimized oxidation state sample (Os‐N_3_S_1_; 22 mV) is still higher than that of Pt/C (13 mV) at 10 mA cm^−2^, suggesting that it is still necessary to work on the materials to decrease the overpotential.^[^ ^180^ ^]^

**Figure 20:**
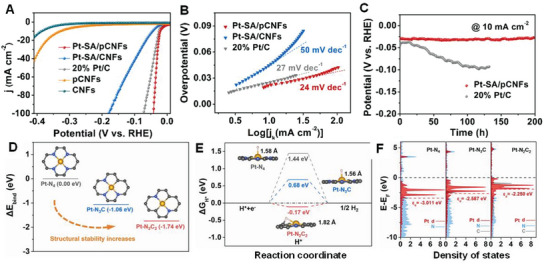
A) LSV‐HER polarization curves of the different materials. B) Tafel slopes. C) Constant‐potential durability of the Pt‐SA/pCNFs and commercial material (Pt/C). D) Binding energy of different Pt motifs. E) Gibbs free energy diagram of different Pt motifs. F) PDOS of Pt‐N_4_, Pt‐N_3_C, and Pt‐N_2_C_2_ configurations. Reproduced with permission.^[^ ^48^ ^]^ Copyright 2023 Elsevier B.V.

In 2023, Zhang et al.^[^ ^48^ ^]^ reported the fabrication of nitrogen‐doped porous carbon nanofiber (pCNFs) as scaffolds to embed well‐defined Pt SMAs (Pt‐SMAs/pCNFs). The efficient adsorption of Pt precursors can be attributed to the excellent hydrophilicity and strong microporous capillary forces in pCNFs. The Pt‐SMAs/pCNFs exhibited excellent HER activity and stability in an acidic medium, which was better than commercial (Pt/C) catalysts (**Figure** **20**A–C).^[^ ^48^ ^]^ Among the various atomic configurations, Pt‐N_2_C_2_ is the most stable structure because of its negative ΔE_bind_ value (−1.74 eV) (Figure 20D).^[^ ^48^ ^]^ DFT calculation reveals that the Gibbs free energy of H adsorption (ΔG_H*_) for a Pt‐N_2_C_2_ model is close to zero (ΔG_H*_ = −0.17 eV), suggesting that its superior HER activity (Figure 20E).^[^ ^48^ ^]^ Projected density of states (PDOS) shows that the Pt 5*d* orbitals in Pt‐N_2_C_2_ (−2.250 eV) shift to higher levels than that in Pt‐N_3_C (−2.587 eV) and Pt‐N_4_ (−3.011 eV), suggesting Pt‐N_2_C_2_ exhibits better HER activity than Pt‐N_3_C and Pt‐N_4_ (Figure 20F).^[^ ^48^ ^]^ Recently, Zang et al. investigated DMAs.^[^ ^207^ ^]^ They reported that Ni and Fe metal atoms (23 mV and 42 mV in acidic and basic, respectively) displayed a surprisingly low overpotential at 10 mA cm^−2^ in acidic/alkaline solutions, which is much better than SMAs and comparable to Pt/C.^[^ ^207^ ^]^ The higher catalytic activity was attributed to the introduction of Ni atoms, which increased the electron transfer rate between the two layers and weakened the adsorption of intermediates, resulting in an optimized energy level of the *d*‐orbital and improved reaction activity.^[^ ^207^ ^]^

**Figure 21:**
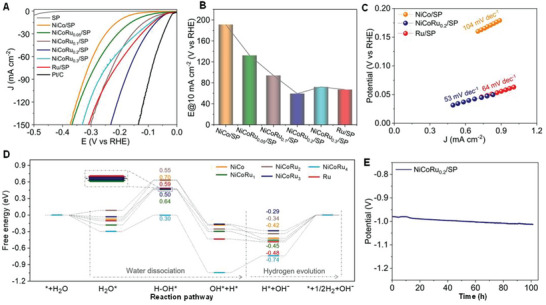
A) LSV‐HER polarization curves of the different materials. B) Overpotential of different materials at a fixed current density. C) Tafel slopes of various materials. D) Calculated energy barriers (water dissociation and hydrogen desorption) at different surfaces. E) Constant current density stability test. Reproduced with permission.^[^ ^133^ ^]^ Copyright 2023 Elsevier B.V.

Huang et al. reported TMAs (atomic Ru‐doped NiCo) for hydrogen in alkaline media.^[^ ^133^ ^]^ TMAs are deposited on the supra‐P (SP) carbon, which acts as a substrate. A tuned NiCoRu_0.2_/SP material reached 10 mA cm^−2^ at a very low overpotential (59 mV) with a small Tafel slope (**Figure** **21**A–C).^[^ ^133^ ^]^ Furthermore, the catalytic activity was not increased, even though the amount of Ru is increased in NiCo using the excessive Ru not needed to obtain the low overpotential. The DFT calculation showed that the incorporation of Ru in NiCo would have a synergistic effect on the Ru‐Ni/Co interface, which accelerates the water dissociation process and adjusts the adsorption/desorption energetics towards the H intermediate, leading to an increase in HER activity and stability (Figure 21D,E).^[^ ^133^ ^]^ In 2022, Ding et al.^[^ ^198^ ^]^ also synthesized TMAs (Zn‐Fe/Mn@Mn‐FeP; denoted as FMZP4) and evaluated their hydrogen generation activity. FMZP4 revealed low overpotentials (53 mV) to achieve 10 mA cm^−2^ for hydrogen generation.^[^ ^198^ ^]^ This material also exhibited good stability over 80 h.^[^ ^198^ ^]^ The high activity and stability were attributed to fast electron and mass transport channels, high conductivity, and large accessible active surface area.^[^ ^198^ ^]^ Researchers have also reported high‐entropy SAs (HESAs) with the coexistence of MMAs to overcome the potential barriers of hydrogen production in acidic and alkaline media.

Zou et al.^[^ ^76^ ^]^ synthesized MMAs (Pt, Ir, Cu, Ni, and Co atoms; HEAs) on the as‐produced defects (carbon black; CB) via electronic interactions using a laser‐planting method. The total MMA loading in defective CB support is 10.8 wt%.^[^ ^76^ ^]^ These HEAs exhibited an onset overpotential of ∼0 mV, and the mass activity was 11 times higher than that of the state‐of‐the‐art catalyst (Pt/C).^[^ ^76^ ^]^ This HEA material also showed a smaller overpotential at 10 mA cm^−2^.^[^ ^76^ ^]^ Wang et al.^[^ ^140^ ^]^ developed a wet chemical method to fabricate stretchable Pt(Co/Ni)MoPdRh nanoflowers (NFs). The mass activity of Pt(Co/Ni)MoPdRh is 16.64 A mg_HEA_^−1^, which was 6.38‐fold larger than of Pt/C.^[^ ^140^ ^]^ The high activity and stability were attributed to the different types of active sites in MMAs, which decrease the water dissociation energies and favor the adsorption of atomic hydrogen and the formation of molecular hydrogen via a moderate Pt–H interaction.^[^ ^140^ ^]^ Overall, SMAs/DMAs/TMAs/MMAs have high HER performance. On the other hand, the ultimate target of various metal atoms used in commercial‐level hydrogen generation devices was not achieved because of their susceptibility to corrosion in acidic, alkaline, and organic environments.

### 6.2 Oxygen Evolution Reaction

One of the best solutions to pursue sustainable energy conversion is to develop an electrochemical water splitting (2H_2_O → 2H_2_ + O_2_) system to cleanly produce hydrogen, which is combined with intermittent renewable electricity. On the other hand, the application of electrochemical water splitting is hindered by the sluggish kinetics of the OER,^[^ ^208^ ^]^ where the rate‐limiting step contains a complex‐coupled transfer process of four protons and four electrons with high energy barriers.

The detailed reaction mechanisms are provided in the previously reported review.^[^ ^209^ ^]^ Following these problems, electrocatalysts, including nanoclusters, nanoflowers, core‐shell, nano‐wrinkles, and nanoneedle structures, have been synthesized. The fabrication of these nanostructured involves complex steps and harsh conditions. Therefore, it is difficult to scale up for the industrial application. The SMA/DMA/TMA/MMA‐based materials were synthesized for the OER to overcome the above issues, which decreased the cost of catalysts and showed promising activity toward the OER. **Tables** **6** and **7** list some acidic and basic OER materials reported thus far.

**Table 6:** OER performance of SMA/DMA/TMA/MMA catalysts in acidic media.

Materials	Electrolyte	Overpotential @ 10 mA cm^−2^	Tafel slope [mV dec^−1^]	Stability	Reference
V@NMCNFs (SMAs)	0.5 m H_2_SO_4_	196	25	60 h	[103]
Nd_0.1_RuO_x_ (SMAs)	0.5 m H_2_SO_4_	211	50	25 h	[210]
CoO_x_/RuO_x_‐CC (SMAs)	0.5 m H_2_SO_4_	180	61.2	100 h	[211]
CeO_2_/Co‐Ni–P–O_x_ (DMAs)	0.5 m H_2_SO_4_	262	32.9	5 h	[212]
Ca_2_Y_0.2_Ir_0.8_O_4_ (DMAs)	1.0 m HClO_4_	213	44.5	168 h	[213]
NiFe@MoS_2_ (DMAs)	0.5 m H_2_SO_4_	201	48.3	16 h	[214]
CPF‐Fe/Ni (DMAs)	0.5 m H_2_SO_4_	201	169.5	200 h	[207]
IrGa‐IMC (DMAs)	0.1 m HClO_4_	272	57.2	1.67 h	[215]
Y_2_RuMnO_7_ (TMAs)	0.1 m HClO_4_	260	48	45 h	[216]
ZnNiCoIrMn (MMAs)	0.1 m HClO_4_	237	46	100 h	[145]

**Table 7:** OER performances for SMA/DMA/TMA/MMA catalysts in alkaline media.

Materials	Electrolyte	Overpotential @ 10 mA cm^−2^	Tafel slope [mV dec^−1^]	Stability	Reference
P/Fe−N−C (SMAs)	0.1 m KOH	304	65	NA	[217]
Co−N_x_−C (SMAs)	1 m KOH	351	84	300 h	[218]
m‐NiTPyP/CNTs (SMAs)	1 m KOH	267	33.1	60 h	[193]
Ni@CN_x_ (SMAs)	1 m KOH	360	260	100 cycles	[194]
Cu‐Co/NC (DMAs)	0.1 m KOH	335	83.8	3K cycles	[219]
FeN_4_‐SC‐NiN_4_ (DMAs)	0.1 m KOH	246	74.0	5K cycles	[119]
RuCo‐CAT (DMAs)	1 m KOH	200	45.7	15 h	[220]
Ni_2_Fe_1_ Sq‐zbr‐MOF (DMAs)	1 m KOH	230	37.7	16.7 h	[221]
CPF‐Fe/Ni (DMAs)	1 m KOH	194	102.1	200 h	[207]
NiFe SAC (DMAs)	0.1 m KOH	270	74	72 h	[222]
Fe/Co–N/S_1.9_–C (DMAs)	0.1 m KOH	294	70.7	6.7 h	[223]
Co/Fe (DMAs)	1 m KOH	240	47.9	26 h	[120]
FeCoNiO_x_/C/NF (TMAs)	1 m KOH	221	21	250	[224]
CoN_4_‐C‐NiN_4_‐C‐FeN_4_ (TMAs)	0.1 m KOH	393	75	2K cycles	[18]
Ni_18_Fe_12_Al_70_ (TMAs)	1 m KOH	255	44	100 h	[134]
Zn‐Fe/Mn@Mn‐FeP (TMAs)	1 m KOH	184 @ 20 mA cm^−2^	51.9	80 h	[198]
Fe–Co–Ni@NDC (TMAs)	0.1 m KOH	359	56	24 h	[225]
FeCoNiMo HEA/C (MMAs)	1 m KOH	250	48.0	65 h	[139]
Cu_2_S/CoFeCuOOH (MMAs)	1 m KOH	170	41.0	100 h	[226]
FeCoNiRu (MMAs)	1 m KOH	243	45	40 h	[144]
(Fe_0.2_Co_0.2_Ni_0.2_Cu_0.2_Zn_0.2_)Al_2_O_4_ (MMAs)	1 m KOH	430	NA	5 h	[227]

**Figure 22:**
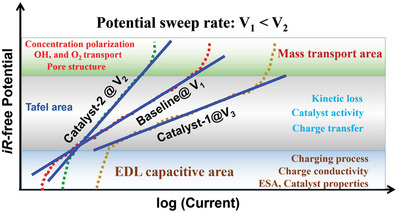
Identification of the electrocatalytic properties related to the OER. EDL: electronic double layer; ESA: electroactive surface area.

**Figure** **22** presents the identification of baselines, Tafel slopes, electronic double layers, and mass transport regions. Xue et al.^[^ ^103^ ^]^ reported a simple method to prepare atomically dispersed V sites embedded on N‐doped carbon nanofibers (called V@NCNFs) for the OER in an acidic electrolyte. The V@NCNFs material showed enhanced catalytic performance because of the presence of penta‐coordinated asymmetric V‐O_2_N_3_ configuration (SMAs), which is a reduced energy barrier to water oxidation, resulting in a low overpotential (196 mV@10 mA cm^−2^), small Tafel slope (25 mV dec^−1^), and extraordinary long‐term stability (60 h).^[^ ^103^ ^]^ Yang et al.^[^ ^210^ ^]^ synthesized Nd‐doped RuO_2_ (Nd_0.1_RuO_x_) that improved the catalytic performance compared to commercial RuO_2_/CC. DFT calculations showed that the d‐band center is shifted away from the Fermi level after Nd doping, lowering the d‐band localization, which weakens the adsorption of key intermediates (oxygen) and boosts the OER kinetics.^[^ ^210^ ^]^ Kibria et al.^[^ ^218^ ^]^ combined the condensation of cobalt phthalocyanine tetramers (CoPc) and melem species (CoMM) with a pyrolysis strategy to synthesize high‐density Co SAs (10.6 wt. %) in a nitrogen‐doped carbon network, which formed the pyridinic Co‐N_4_ sites. CoMM significantly promoted the electrocatalytic OER in 1 m KOH and displayed remarkable stability because of the abundant vicinal Co sites (SMAs).^[^ ^218^ ^]^ The C to N ratio was 1:1, which formed from the fusion of the C_6_N_7_ units of melem.^[^ ^218^ ^]^ The predicted model was well matched with the experiential results. Theoretical calculations showed that facile electron transfer from Co to oxygen species is responsible for the superior OER activity.^[^ ^218^ ^]^ Peng et al. reported a series of pseudo‐pyridine‐substituted Ni(II)‐porphyrins (o‐NiTPyP, m‐NiTPyP, and p‐NiTPyP) with pseudo‐pyridine N‐atoms placed in ortho‐, meta‐, or para‐positions were synthesized and used for alkaline water oxidation.^[^ ^193^ ^]^ They reported that the location of pseudo‐pyridine N‐atom plays a vital role in regulating the active sites and decreasing the overpotential for water oxidation. Among the different positions of pseudo‐pyridine N‐atoms, m‐NiTPyP/CNTs deliver the smallest overpotentials of 267 mV to reach 10 mA cm^−2^.^[^ ^193^ ^]^ In addition, m‐NiTPyP promoted the charge transfer of active sites, which accelerated the OER performance.^[^ ^193^ ^]^ A more recent investigation reveals the origin of the OER performance in P‐Ce SAs@CoO material.^[^ ^228^ ^]^ This model catalyst was synthesized by the effective plasma (P)‐assisted processes. The P‐Ce SAs@CoO displayed the lowest overpotential (261 mV) at 10 mA cm^−2^ and excellent electrochemical durability. In situ Raman analysis showed that the incorporation of Ce atoms could induce electron redistribution and hinder Co–O bond breaking in the Co–O–Ce active site during the OER process.^[^ ^228^ ^]^ The gradient orbital coupling of Ce(4f)‐O(2p)‐Co(3d) enhanced the Co(3d)‐O(2p) covalency, increasing the OER performance.^[^ ^228^ ^]^

**Figure 23:**
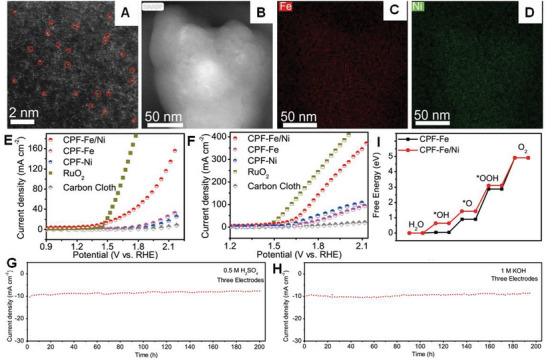
A–D) AC‐HAADF‐STEM image and elemental mapping of the CPF‐Fe/Ni (DMAs). E–H) LSV and chronopotentiometry curves of CPF‐Fe/Ni in acidic (0.5 m H_2_SO_4_; G) and alkaline (1 m KOH; H) media. I) Gibbs free energy profiles of the OER on CPF‐Fe and CPF‐Fe/Ni. Reproduced with permission.^[^ ^207^ ^]^ Copyright 2023 The Author(s).

The intrinsic activity further induced OER catalytic activity, which increases using DMAs. The DMAs tuned the electronic structures, promoting intrinsic activity owing to differences in electronegativity.^[^ ^37^ ^]^ Zang et al.^[^ ^207^ ^]^ fabricated a 2D conjugated phthalocyanine framework (CPF) containing single atomic Ni/N/C and Fe/N/C (called CPF‐Fe/Ni; DMAs) via ion exchange under microwave irradiation. The electronegativities of Fe and Ni ions are different, resulting in an uneven distribution of metal sites during CPF‐Fe/Ni synthesis (**Figure** **23**A–D).^[^ ^207^ ^]^ CPF‐Fe/Ni showed excellent catalytic activities in terms of overpotential (201 mV in 0.5 m H_2_SO_4_ at 10 mA cm^−2^; 194 mV in 1 m KOH at 10 mA cm^−2^) and stability (200 h in both acidic and basic solutions), which are much better than that of the state‐of‐the‐art catalysts (20% Pt/C and RuO_2_; Figure 23E‐H).^[^ ^207^ ^]^ According to the DFT calculations, the energy level tuned after the incorporation of DMAs, suggested a smaller overpotential (ŋ = 0.58 V) than that of pure CPF‐Fe (ŋ = 0.81 V) and accelerating the OER kinetics and intermediate evolution (Figure 23I).^[^ ^207^ ^]^ Qiu et al.^[^ ^119^ ^]^ synthesized separate FeN_4_ and NiN_4_ sites (called FeN_4_‐SC‐NiN_4_) via a layer‐by‐layer space‐confinement approach. Elemental analysis showed that the Fe and Ni content in the FeN_4_‐SC‐NiN_4_ is 0.98 at% and 1.02 at%, respectively.^[^ ^119^ ^]^ The FeN_4_‐SC‐NiN_4_ (DMAs) requires a η of 246 mV in O_2_‐saturated 0.1 m KOH electrolyte, which was much smaller than NiN_4_‐SC (SMAs, 272 mV) and FeN_4_‐SC (SMAs, 401 mV). They reported that the anisotropic modification in the electronic structure adjusted the binding ability for different oxygen‐containing intermediates, improving the catalytic activity of oxygen at two different sites (FeN_4_ and NiN_4_).^[^ ^119^ ^]^ Li et al.^[^ ^120^ ^]^ reported the fabrication of Co−Fe heteronuclear (DMAs) using a microemulsion‐co‐precipitation reaction, pyrolysis, and acid etching strategies. The OER activity of the Co−Fe heteronuclear is determined in O_2_‐saturated 1 m KOH. The optimized Co/Fe‐SNC800 material showed the onset potential (@1 mA cm^−2^), ŋ (@10 mA cm^−2^), Tafel slope, and turnover frequency (@10 mA cm^−2^) were 1.42 V, 240 mV, 47.9 mV dec^−1^, and 146 s^−1^, respectively, which is superior to the commercial catalyst IrO_2_.^[^ ^120^ ^]^ Furthermore, the long‐time stability test (26 h) at 20 mA cm^−2^ displayed that the Co/Fe‐SNC800 material is still active and durable under harsh conditions.^[^ ^120^ ^]^ More importantly, Fe and Co atoms discharge the excessive adsorbed hydroxide ion on the Fe sites and accelerate the production of Co_site_OOH active moieties that can promote the OER activity via the synergistic effect.^[^ ^120^ ^]^ Li et al. proposed a DMA for the OER in 0.1 m KOH with Co‐N_4_ and Cu‐N_4_ moieties anchored in a nitrogen‐doped carbon matrix (Cu‐Co/NC) using a pyrolysis process.^[^ ^219^ ^]^ The optimized catalyst exhibited high catalytic performance for the OER with ŋ (@10 mA cm^−2^) up to 335 mV and demonstrated a Tafel slope of 83.8 mV dec^−1^, which was lower than the other controls, suggesting rapid OER kinetics.^[^ ^219^ ^]^ Furthermore, theoretical calculations indicated that the CuN_4_ active species optimize the electronic configuration of the nearby CoN_4_ active species during the OER process,^[^ ^219^ ^]^ resulting in high OER activity.

Yuan et al. reported the synthesis of NiFe DMAs at the isolated S defects of 2D MoS_2_ by a trapping process.^[^ ^214^ ^]^ The interlayer confined NiFe@MoS_2_ exhibited a lower overpotential (201 mV) and small Tafel slope (46.3 mV dec^−1^) in an acidic electrolyte, which is much better than that of IrO_2_,^[^ ^214^ ^]^ indicating the role of confinement effect in OER performance. DFT calculations also indicated that the extraordinary activities came from confined structures because the energy barrier (Ew) at Fe and Ni sites of 1.2 nm interlayer‐confined NiFe@MoS_2_ are much lower than that on the 1.5 nm interlayer‐confined NiFe@MoS_2_.^[^ ^214^ ^]^

TMAs/MMAs, as an extension of DMAs, have become a topic of intense interest because of their notable benefits in synergistic actions and driving multi‐step catalytic reactions, such as OER. Ding et al. developed Zn‐Fe/Mn‐based TMAs materials with a hierarchical ultrathin nanosheet structure (Mn‐FeP) by chemical etching, thermal oxidation, and in situ phosphatization processes.^[^ ^198^ ^]^ These Zn‐Fe/Mn‐based TMAs showed a very small overpotential of 184 mV at 20 mA cm^−2^, with a Tafel slope of 51.9 mV dec^−1^ and a smaller charge‐transfer resistance.^[^ ^198^ ^]^ Mn–FeP sheets help in fast charge transport and efficient mass transport channels.^[^ ^198^ ^]^ In addition, the Zn‐Fe/Mn‐based TMAs had exceptional stability with almost no change in the distribution of elements after the long‐time tests in an alkaline electrolyte.^[^ ^198^ ^]^ Qiu et al. fabricated Co‐, Ni‐, and Fe‐based TMAs with isolated Co, Ni, and Fe atoms onto the N‐doped carbon support, where Co‐N_4_, Ni–N_4_, and Fe‐N_4_ have been reported to be catalytic‐active sites.^[^ ^18^ ^]^ Such Co‐, Ni‐, and Fe‐based TMAs exhibited much lower η of 393 mV (at 10 mA cm^−2^) and a Tafel slope of 75.0 mV dec^−1^ in a 0.1 m KOH solution in comparison with CoN_4_‐C (467 mV), NiN_4_‐C (404 mV), and FeN_4_‐C (650 mV) catalysts.^[^ ^18^ ^]^ They also reported that incorporating TMAs offers strong electronic reciprocity, with Ni donating electrons to Fe and Co, simultaneously increasing the catalytic activity for the OER.^[^ ^18^ ^]^

Decreasing the overpotential and increasing intrinsic activities still require high current densities, which are achieved by synthesizing MMA‐driven catalysts. In this regard, HEMs (MMAs) are the most promising catalysts. Huang et al. reported a FeCoNiRu high‐entropy alloy (HEA) for the OER in alkaline electrolyte.^[^ ^144^ ^]^ Such HEA was prepared using a high‐entropy MOF (HEMOF) precursor.^[^ ^144^ ^]^ The carbon skeleton derived from the MOF precursor has several advantages, such as higher porosity, rapid transfer of reaction species through MOF channels, and aggregation of metal ions.^[^ ^144^ ^]^ The optimized FeCoNiRu HEA materials possessed a Tafel slope of 45 mV dec^−1^ and a minimum overpotential value of only 243 mV at 10 mA cm^−2^, which are lower than that of commercial RuO_2_ and commercial Pt/C.^[^ ^144^ ^]^ Furthermore, the catalytic activity was maintained after the 40 h stability test.^[^ ^144^ ^]^

Song et al. synthesized the ZnNiCoIrMn HEA materials for OER in an acidic medium.^[^ ^145^ ^]^ The presence of a low Ir content in ZnNiCoIrMn delivered low overpotential (237 mV@10 mA cm^−2^) and excellent stability (100 h) for the OER in an acidic medium.^[^ ^145^ ^]^ The DFT results suggest that the optimized adsorption energies are due to modification of the electronic structure of ZnNiCoIrMn HEA, resulting in higher OER activity.^[^ ^145^ ^]^ Overall, SMA/DMA/TMA/MMA‐based catalysts exhibited remarkable activity toward the OER. The main challenge was that the loading of active metals remains relatively low, and the overpotential was still high at large current densities for SMAs/DMAs/TMAs/MMAs, limiting further industrial use.

### 6.3 Membrane‐Based Water Electrolyzers

**Figure 24:**
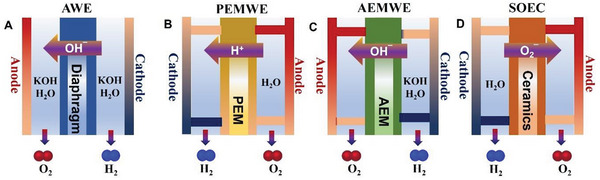
Schematic illustrations of A) AWE, B) PEMWE, C) AEMWE, and D) SOEC.

**Figure 25:**
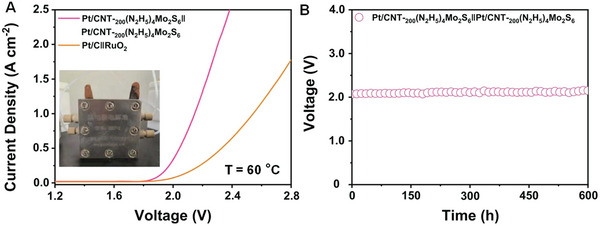
A) AEM electrolyzer testing at 60 °C. The inset shows a picture of the AEM setup. B) Chronopotentiometry performance at 1 A cm^−2^. Reproduced with permission.^[^ ^231^ ^]^ Copyright 2023 Elsevier B.V.

Membrane‐based water electrolyzers are a burgeoning field for hydrogen and oxygen production, in which various electrolyzers emerge. Thus far, different types of electrolyzers have been developed, e.g., alkaline water electrolyzer (AWE), proton exchange membrane water electrolyzer (PEMWE), anion‐exchange membrane water electrolyzer (AEMWE), and solid oxide electrolysis cell (SOEC).^[^ ^229^ ^]^ AWE and PEMWE are industrial‐adopted technologies, while AEMWE and SOEC have newly introduced water electrolysis technologies (**Figure** **24**). AWE uses highly concentrated KOH electrolyte (20–40% KOH and operated at 60–80 °C) and is used more widely owing to its low‐cost configuration.^[^ ^229^ ^]^ On the other hand, the main problem with AWE is that it needs a long start‐up preparation and exhibits a slow response to the changes in electrical power. In addition, the diaphragm does not stop the passage of gases. Thus, some O_2_ reaches the cathode and combines with H_2_ to form H_2_O, resulting in reduced efficiency of the whole cell, causing safety issues. By contrast, PEMWE operates at low temperatures (25–80 °C), superb conversion efficiency (80–90%), high H_2_ purity (>99.99%), and delivers large current densities with a rapid response under electrical energy supply.^[^ ^229^ ^]^ On the other hand, AEMWE requires a heat exchange method (HEM) to produce the stable migration of OH^–^ ions, whereas SOEC requires high pressures and temperatures to transport O^2–^ ions.^[^ ^229^ ^]^ Overall, AEMWE and PEMWE have unique attention to hydrogen production. Therefore, this paper reports the latest approaches to fabricating water electrolysis devices. In particular, the device preparation based on SMA/DMA/TMA/MMA materials is discussed. In this regard, Lee et al.^[^ ^230^ ^]^ reported single Pt atom‐embedded metal hydroxides {(Ni_2_(OH)_2_(NO_3_)_2_; NiNH} and NiNH for hydrogen generation and oxygen production, respectively. The optimized Pt_SA−1.73_–NiHN material showed the η of 24 mV at 10 mA cm^−2^ for the HER, whereas the NiNH needs an η of 280 mV at 50 mA cm^−2^ for the OER in a 1 m KOH solution.^[^ ^230^ ^]^ When assembled in the two‐electrode configuration, a cell voltage of 1.45 V requires 10 mA cm^−2^ for full water splitting, which is superior to commercial‐adopted catalysts.^[^ ^230^ ^]^ This device also exhibited long‐term stability. The high activity and stability were attributed to the electron‐modified Ni with favorable free energies for adsorption.^[^ ^230^ ^]^ Zhao et al. used Pt/CNT materials modified by molecular metal chalcogenide complexes (denoted as Pt/CNT‐200(N_2_H_5_)_4_Mo_2_S_6_).^[^ ^231^ ^]^ The function of metal chalcogenide complexes is to prevent the formation of insoluble precipitates during the electrolysis of alkaline seawater, helping improve the stability of H_2_ production. This was confirmed by fabricating the AEMWE and checking their activity and stability. They reported that modified Pt/CNT material has excellent stability (600 h@1.0 A cm^−2^) at a large current density and exhibited an industrially required current density (1.0 A cm^−2^@2.0 V; 60 °C; **Figure** **25**).^[^ ^231^ ^]^ Huang et al.^[^ ^232^ ^]^ introduced single‐site Pt into the RuO_2_ hollow nanospheres (SS Pt‐RuO_2_ HNSs) with interstitial C that acted as a catalyst for whole water splitting in an acidic medium. In particular, when SS Pt­RuO_2_ HNSs were exploited as material for acidic water splitting, the cell voltages required were 1.49, 1.59, and 1.65 V for achieving current densities of 10, 50, and 100 mA cm^−2^, respectively, which is far better than that of benchmark materials (commercial Pt/C and RuO_2_ materials for cathode and anode).^[^ ^232^ ^]^ The fabricated PEMWE device exhibited excellent durability with a small cell voltage change after 100 h.^[^ ^232^ ^]^ The DFT results suggested that effective synergy increases the OER activity by lowering the energy barriers and improving the dissociation energy of the *O moieties.^[^ ^232^ ^]^ Although developing effective catalysts is the best strategy to reduce the overpotential, more work is needed on active materials. Furthermore, the synthesis and design of new SMA/DMA/TMA/MMA materials are urgently needed to reduce the overpotential and increase the stability.

### 6.4 Oxygen Reduction Reaction

The electrochemical ORR in acidic or alkaline environments is a key reaction in energy conversion and storage devices, such as PEM fuel cells, alkaline fuel cells, and Zn/air batteries. The efficiency of energy conversions and storage devices is mainly determined by the ORR occurring at the cathode because the ORR rate is much slower than the hydrogen oxidation reaction (HOR) at the anode. Pt‐based materials are excellent catalysts for both reactions. On the other hand, the scarcity of Pt and its high price motivate researchers to explore low‐cost, non‐precious substitutes to replace commercial Pt/C. For the ORR, heteroatom‐coordinated transition‐metal catalysts have attracted increasing interest in the last year because of their good electrical conductivity, high catalytic activity, and excellent corrosion resistance. The application parts discuss the fabrication of devices and their performance separately. This section discusses OER catalysts based on SMA/DMA/TMA/MMA materials. **Tables** **8** and **9** list the SMA/DMA/TMA/MMA‐based materials for enhanced ORR activity in acidic and alkaline media.

**Table 8:** ORR performance of SMA/DMA/TMA/MMA materials in acid media.

Electrocatalysts	Electrolyte	E_1/2_ [V vs RHE]	Reference
SnN_3_O‐50 (SMAs)	0.1 m HClO_4_	0.816	[233]
Zn‐MOF‐74 NPC (SMAs)	0.1 m HClO_4_	0.698	[234]
FeN_4_‐hcC (SMAs)	0.5 m H_2_SO_4_	0.85	[235]
Co‐SAs/N‐C/rGO (SMAs)	0.5 m H_2_SO_4_	0.77	[236]
Fe–N–C (SMAs)	0.1 m HClO_4_	0.56	[237]
S_1_‐Cr_1_N_4_‐C (SMAs)	0.1 m HClO_4_	0.72	[238]
FeSNC (SMAs)	0.5 m H_2_SO_4_	0.76	[239]
COP_BTC_@Cl‐CNTs (SMAs)	0.1 m HClO_4_	0.75	[240]
TAP 900@Fe (SMAs)	0.1 m HClO_4_	0.77	[241]
Sb–N/C (SMAs)	0.5 m H_2_SO_4_	0.70	[242]
Cu SAC/P‐700 (SAMs)	0.1 m HClO_4_	0.75	[243]
Zn/CoN‐C (DMAs)	0.1 m HClO_4_	0.79	[244]
FeMo–N–C (DMAs)	0.1 m HClO_4_	0.84	[245]
Pt_4_FeCoCuNi (MMAs)	0.1 m HClO_4_	0.94	[203]

**Table 9:** ORR performance of SMA/DMA/TMA/MMA materials in alkaline solutions.

Electrocatalysts	Electrolyte	E_1/2_ [V vs RHE]	Reference
SnN_3_O‐50 (SMAs)	0.1 m KOH	0.905	[233]
Zn‐MOF‐74 NPC (SMAs)	0.1 m KOH	0.902	[234]
Co‐SAs/N‐C/rGO (SMAs)	0.1 m KOH	0.84	[236]
Fe–N–C (SMAs)	0.1 m KOH	0.53	[237]
P/Fe–N–C (SMAs)	0.1 m KOH	0.90	[217]
S_1_‐Cr_1_N_4_‐C (SMAs)	0.1 m KOH	0.90	[238]
FeSNC (SMAs)	0.1 m KOH	0.91	[239]
Cu‐N_4_ (SMAs)	0.1 m KOH	0.89	[246]
Cu‐S_1_N_3_/Cux (SMAs)	0.1 m KOH	0.90	[107]
NiFe‐LDH/Fe_1_‐N‐C (SMAs)	0.1 m KOH	0.90	[247]
Sb–N/C (SMAs)	0.1 m KOH	0.89	[242]
Co‐SA@N‐CNFs (SMAs)	0.1 m KOH	0.85	[248]
Cu SAC/P‐700 (SAMs)	0.1 m KOH	0.87	[243]
Zn/CoN‐C (DMAs)	0.1 m KOH	0.86	[244]
FeN4‐SC‐NiN4	0.1 m KOH	0.84	[119]
Cu‐Co/NC (DMAs)	0.1 m KOH	0.92	[219]
Fe/Co–N/S_1.9_–C (DMAs)	0.1 m KOH	0.84	[223]
FeCu‐SAC (DMAs)	0.1 m KOH	0.93	[249]
FePtNC (DMAs)	0.1 m KOH	0.90	[127]
Co‐Te DASs/N‐C (DMAs)	0.1 m KOH	0.85	[250]
Fe–Co–Ni (TMAs)	0.1 m KOH	0.90	[225]
Co_2_MnN_8_/C (TMAs)	0.1 m KOH	0.91	[251]
HEA‐NPs‐(14) (MMAs)	0.1 m KOH	0.86	[190]
PdNFe_3_@Pd/C (MMAs)	0.1 m KOH	0.91	[252]

Among the various types of SMAs, Fe‐based materials are very active for the ORR in acidic and basic solutions.^[^ ^236^, ^237^, ^240^, ^242^ ^]^ Feng et al.^[^ ^235^ ^]^ reported Fe–N–C materials with dense FeN_4_ sites on a highly curved carbon surface, which can effectively modulate the electronic structures of Fe *d*‐band centers and prevent the adsorption of oxygenated moieties, resulting in a higher half‐wave potential (E_1/2_) of 0.85 V (vs reversible hydrogen electrode; RHE) and stability (30 000 cycles) in acidic solutions.^[^ ^235^ ^]^ Similarly, Titirici et al.^[^ ^241^ ^]^ prepared a highly porous Fe–N–C catalyst (≈3295 m^2^ g^−1^) with outstanding Fe utilization for the ORR in acidic media. This material was synthesized by the pyrolysis of 2,4,6‐triaminopyrimidine (TAP) in the presence of Mg^2+^ salt at temperatures between 800 and 1000 °C. Among the various temperatures, the TAP‐900@Fe sample (fabricated at 900 °C) showed the highest catalytic activity due to the abundance of FeN_x_ (52%) active sites, which are penta‐coordinated via an axial ligand.^[^ ^241^ ^]^ Xu et al.^[^ ^247^ ^]^ reported a NiFe‐layered double hydroxide (NiFe‐LDH)/ Fe_1_–N–C heterostructure that enhances the ORR activity and performs an efficient bifunctional ORR/OER activity in a monolithic catalyst. They found that NiFe‐LDH/Fe_1_–N–C nanorods have higher ORR activity with an E_1/2_ of 0.90 V vs RHE, which is better than that of bare Fe_1_‐N‐C and commercial Pt/C.^[^ ^247^ ^]^ DFT calculations showed that NiFe‐LDH donated electrons to the Fe_1_–N–C hybrid and reduced the Fe *d*‐band center, drastically lowering the ORR rate‐determining‐step energy barriers.^[^ ^247^ ^]^ Overall, Fe‐based SMAs with varied electronic structures can show several spin states (low‐spin, medium‐spin, and high‐spin) and numerous valence states (Fe(II), Fe(III), and Fe(IV)). The Fe(II) and Fe(III) states are prevalent, low‐ or medium‐spin FeN_4_ moieties that are considered the main catalytic sites for the ORR activity in Fe–N–C materials. The P, S, and O functionalities in M–N–C (M = Fe, Co, Cu, and Cr) further regulated the electronic structures of M‐N_4_ sites, which weaken the adsorption of ORR intermediates, indicating the remarkable ORR activity and stability in acidic and alkaline electrolytes.^[^ ^107^, ^233^, ^238^, ^239^, ^242^, ^248^ ^]^ For example, Cho et al.^[^ ^253^ ^]^ fabricated P‐doped Fe−N−C materials. They confirmed the formation of the FeN_3_PO moiety, which promoted *OH desorption due to the Fe d‐band center downward from −0.84 to −1.06 eV via P doping, thereby improving the ORR activity. Lin et al.^[^ ^254^ ^]^ synthesized a Fe‐SA/PNC catalyst with a FeN_2_P_2_ site using a chemical vapor deposition strategy. The density of Fe atoms was 3.97% and coordinated with N‐ and P‐doped carbon sheets.^[^ ^254^ ^]^ Consequently, the Fe‐SA/PNC material exhibited a half‐wave potential of 0.92, 0.83, and 0.86 V (vs RHE) in alkaline, neutral, and acidic electrolytes, suggesting excellent catalytic activity towards the ORR over the entire pH range.^[^ ^254^ ^]^ The Fe‐SA/PNC material also showed outstanding durability by the lack of shift in the potential after 30 000 cycles.^[^ ^254^ ^]^ The catalytic activity and stability were attributed to the enhancements of the intrinsic activity of the Fe‐N_4_ motif by introducing P, which reduces the energy barrier for the final OH* desorption step.^[^ ^254^ ^]^ Similarly, P and S also modified the electron structure metal–nitrogen coordination moieties (M–N_x_) sites.^[^ ^107^, ^238^, ^239^ ^]^ In this regard, Han et al. constructed Fe‐N_4_ motifs with S functionalities (FeSNC) and found that the optimized material has very good ORR activity in the full pH range (E_1/2_ of 0.76 V in 0.5 m H_2_SO_4_ and 0.91 V in 0.1 m KOH).^[^ ^239^ ^]^ The DFT results suggested that thiophene S and oxidized S have electron‐liberating characteristics for adjusting the electronic structure of Fe–N_4_ motifs, weakening the adsorption of the ORR intermediates, and accelerating the catalytic activity.^[^ ^239^ ^]^ Similarly, Zhang et al.^[^ ^238^ ^]^ reported that chromium (Cr)‐N_4_ material contains S that transformed into axial configurations S_1_‐Cr_1_N_4_ (S_1_–Cr_1_N_4_–C) during polymerization and confined pyrolysis processes. Intrinsic activity is promoted by modifying the coordination structure and electronic distribution of the M center through the axial motif (S_1_–Cr_1_N_4_–C) and accelerated ORR process (E_1/2_ of 0.90 V vs. RHE).^[^ ^238^ ^]^ In addition to single Fe atoms, exceptional ORR performances are achieved by Sn, Co, and Cu metal atoms. For example, Zhao et al.^[^ ^233^ ^]^ constructed a p‐block SMAs (Sn) with hierarchical pore structures using a soft template strategy. By tuning the pore structures, highly exposed Sn active species with N/O coordination were attained.^[^ ^233^ ^]^ These active sites in the SnN_3_O material showed an E_1/2_ value of 0.816 V (vs RHE) with a small change in potential (15 mV) after 10 000 cycles.^[^ ^233^ ^]^ According to the DFT results, the N/O coordination promotes the localization of outer 5p electrons by Sn species, which enhances the O_2_ adsorption via a strong *p*–*p* orbital coupling effect, regulating the energy barrier of the four successive ORR pathways.^[^ ^233^ ^]^ Sun et al.^[^ ^248^ ^]^ synthesized atomically dispersed Co–N_4_O motifs embedded on N‐doped carbon nanofibers (denoted as Co‐SA@N‐CNFs) using a predesigned phenolic resin‐mediated method. They reported that the O atom is in the axial direction perpendicular to the Co‐N_4_ active sites and plays a major role in increasing the ORR activity.^[^ ^248^ ^]^ These Co‐N_4_O active sites showed that the E_1/2_ is 0.85 V (vs. RHE) in an alkaline solution.^[^ ^248^ ^]^ The Co‐N_4_O configuration can facilitate the regulation of reaction steps and adjust the bond length between the Co motifs and the intermediate species, significantly decreasing the dissociation energy and boosting the ORR activity.^[^ ^248^ ^]^ Yuan et al.^[^ ^243^ ^]^ developed P‐doped Cu single atoms (denoted as Cu SAC/P) that exhibited an outstanding E_1/2_ value of 0.87 V (RHE). The good ORR performance was attributed to P tuning the density of states and modifying the d‐band center of Cu atoms, which enhanced the adsorption of the reaction intermediates and improved the ORR performance.^[^ ^243^ ^]^ Cao et al.^[^ ^246^ ^]^ designed Cu SMAs and examined their ORR activity. They reported that the optimized Cu SMAs with controllable carbon defects have high ORR activity with an E_1/2_ value of 0.897 V (RHE)^[^ ^246^ ^]^ Theoretical calculations reported that the O–O bond in the OOH* intermediate can be weakened by suitable carbon defects around the Cu‐N_4_ motifs, resulting in a reduced free energy barrier of OOH* species and accelerating the overall ORR process.^[^ ^246^ ^]^

The catalytic activity of SMAs remains unsatisfactory because the adsorption behaviors of the intermediates during the ORR at the M–H_x_ (M: Fe, Co, Cu, Sn, and H_x_: heteroatoms and carbon) sites are too strong. Therefore, the reaction kinetics barrier is still downshifted by O–O bond breaking and the desorption pathway. In this issue, DMA sites prefer to adsorb ORR intermediates via a bridging‐*cis*/*trans* adsorption pattern, which is further down the *d*‐band center of SMA and favors O–O bond breaking, improving the ORR catalytic activity.^[^ ^119^, ^223^, ^244^, ^249^, ^250^, ^255^ ^]^ Qiu et al.^[^ ^119^ ^]^ synthesized a sulfur‐promoted anisotropic electronic modulation for Fe/Ni dual‐atom moieties (called FeN_4_‐SC‐NiN_4_; DMAs) for the ORR in basic solutions. They reported that the S reduces the charges of the FeN_4_ moieties decrease the *d*‐band center of the NiN_4_ moieties.^[^ ^119^ ^]^ Therefore, a large difference in the electronic distribution occurred, which enhanced OH* desorption at the FeN_4_ moieties but accelerated O_2_* stripping at the NiN_4_ moieties, allowing FeN_4_‐SC‐NiN_4_ (DMAs) sites to exhibit increased oxygen reduction activity (E_1/2_ = 0.844 V vs. RHE).^[^ ^119^ ^]^

**Figure 26:**
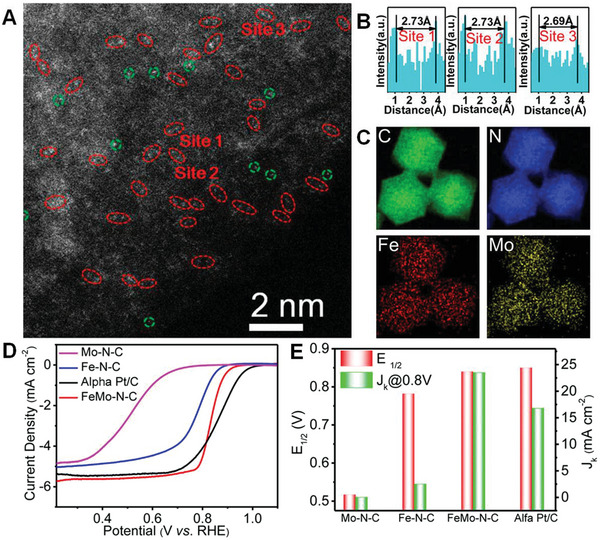
A) AC HAADF‐STEM image of FeMo–N–C. B) The intensity profiles were obtained from the marked areas of 1–3 in (A). C) Elemental mapping images of FeMo–N–C. D) ORR activities of FeMo–N–C, Fe–N–C, Mo–N–C, and commercial 20 wt% Pt/C in oxygen‐saturated 0.1 m HClO_4_ solution. E) Half‐wave potential (E*_1/2_*) and kinetic current density (j_k_) of ORR at 0.8 V for different materials. Reproduced with permission.^[^ ^256^ ^]^ Copyright 2022 American Chemical Society.

Li et al.^[^ ^256^ ^]^ modulated the FeN_x_ site (i.e., downward shift of the Fe d‐orbital center) by incorporating Mo atoms. The Fe–Mo (DMAs) atom‐coordinated N‐doped carbon (FeMo–N–C) was synthesized using host–guest chemistry.^[^ ^256^ ^]^ The AC HAADF‐STEM image (**Figure** **26**A) revealed no metallic NPs over the N‐doped carbon sheets. The gaps between the two conjoined metal atoms (Fe–Mo DMAs) in the areas marked 1, 2, and 3 were 0.27 nm (Figure 26B).^[^ ^256^ ^]^ The EDS maps of the FeMo–N–C material revealed the elemental distributions of Fe, Mo, and N at the N‐doped carbon sheets (Figure 26C).^[^ ^256^ ^]^ The above analysis confirmed the presence of Fe–Mo DMAs. The well‐defined Fe–Mo DMAs delivered an E_1/2_ value of 0.84 V (vs. RHE), which is best among the as‐synthesized materials (Figure 26D)^[^ ^256^ ^]^ Furthermore, the kinetic current density (j_k_; 23.5 mA cm^−2^ @ 0.8 V vs RHE) of FeMo–N–C was also far better than the control samples (Fe–N–C and Mo–N–C), showing the synergistic effect between the Fe–N–C and Mo–N–C motifs (Figure 26E).^[^ ^256^ ^]^ Zhang et al.^[^ ^223^ ^]^ constructed Fe/Co–N/S_x_–C (DMAs) materials by doping various amounts of S. Under the optimized conditions, Fe/Co–N/S_1.9_–C material displays an E_1/2_ of 0.84 V and a limiting current density (j_l_) of 5.4 mA cm^−2^, surpassing the other synthesized materials.^[^ ^223^ ^]^ The remarkable activity of the Fe/Co–N/S_1.9_–C material was attributed to the regulated coordination environment and the altered local strain of the active sites.^[^ ^223^ ^]^ Strasser et al.^[^ ^257^ ^]^ reported the FeSnNC and FeCoNC materials and compared the ORR activity with the FeNC and SnNC (SMAs) materials. Physical and chemical analysis revealed Fe‐N_x_ and Sn‐N_x_ or Co‐N_x_ species, whereas no proof was observed for binuclear Fe‐M‐N_x_ moieties.^[^ ^257^ ^] 57^Fe Mössbauer spectroscopy revealed a different spectral signature (higher D1/D2 ratio corresponded to two distinct Fe‐N_x_ motifs) for FeSnNC and FeCoNC materials compared to SnNC and CoNC.^[^ ^257^ ^]^ They reported that the mass activity of FeSnNC and FeCoNC is even higher than the FeNC and SnNC (SMAs) materials, indicating the importance of DMAs.^[^ ^257^ ^]^ Wang et al.^[^ ^249^ ^]^ synthesized Fe,Cu DMAs using a ligand‐mediated strategy, which is dispersed atomically at carbon black in the form of FeN_4_ and CuN_4_ motifs. The as‐fabricated Fe,Cu DMAs showed higher ORR activity in terms of E_1/2_ (0.926 V vs RHE) and good stability in basic media, which was superior catalytic activity to the corresponding Fe‐SMAs and Cu‐SMAs.^[^ ^249^ ^]^ According to theoretical analysis, the excellent activity was attributed to the modification of Fe motifs by Cu‐SMAs, resulting in regulated adsorption/desorption barrier energies for ORR intermediates and facilitating the ORR intermediates and ORR activity.^[^ ^249^ ^]^ Fan et al.^[^ ^127^ ^]^ conducted an experimental and theoretical investigation of atomically dispersed Fe–Pt DMAs (called FePtNC). The FePtNC material exhibited a superb E_1/2_ (0.90 V vs RHE) for the ORR, which is significantly higher than that of the SMAs and nanocatalysts, owing to the electronic modulation effect between the adjacent Fe and Pt atoms.^[^ ^127^ ^]^ Wang et al.^[^ ^219^ ^]^ fabricated a material of copper‐cobalt DMAs on a nitrogen‐doped carbon matrix (denoted as Cu–Co/NC) by pyrolyzing Zn‐Co MOF/Cu@polymer under an inert gas, which achieved an E_1/2_ value of 0.92 V for the ORR in an alkaline medium. They reported outstanding ORR activity in acidic (0.85 V) and neutral (0.74 V) electrolytes.^[^ ^219^ ^]^ The Cu–Co DMA motifs with metal‐N_4_ sites induced asymmetric charge distributions that modified the adsorption/desorption behavior with oxygen intermediates, enhancing the ORR.^[^ ^219^ ^]^ On the other hand, DMAs enhanced the ORR activity and stability because of a downshift of the d‐band center of SMAs by the existence of other SMAs sites. Nevertheless, details of the modulation and interaction effect of the dual‐metal configuration at the atomic level are yet to be revealed.

Like DMAs, TMAs have several benefits, such as alterable composition, tailorable active sites, and optimizable electronic distribution.^[^ ^135^, ^225^, ^251^ ^]^ Hu et al.^[^ ^225^ ^]^ reported the tri‐metal Fe–Co–Ni embedded N‐doped carbon for ORR in an alkaline solution. The optimized composition (TMAs) exhibited an E_1/2_ value of 0.902 V (vs RHE) toward the ORR,^[^ ^225^ ^]^ surpassing the control samples. The high activity was attributed to the modified d‐band center and the electronic structures, reducing the free energy barriers for the ORR intermediates during the electrocatalytic process.^[^ ^225^ ^]^ Zhang et al.^[^ ^135^ ^]^ developed a TMA consisting of Cu_1_Au_1_ at Cu_1_Pd_3_ sites implanted on N‐doped graphene sheets using an annealing strategy (Cu_1_Au_1_@Cu_1_Pd_3_ NDs/NGS‐A). The as‐synthesized Cu_1_Au_1_@Cu_1_Pd_3_ NDs/NGS‐A demonstrates excellent catalytic activity (E_1/2_ of 0.90 V vs RHE)) toward the ORR in alkaline electrolytes.^[^ ^135^ ^]^ In this TMA structure (Cu–Au–Pd), regulated electronic structures of the metal and dragged down the metal d‐band center, weakening intermediate adsorption, protecting the active sites, and accelerating the ORR kinetics.^[^ ^135^ ^]^ More recently, nitrogen‐coordinated one Mn atom adjacent to two Co atoms (Co_2_MnN_8_) implanted in N‐doped carbon exhibited outstanding ORR performance (E_1/2_ = 0.912 V vs RHE) together with superior stability in 0.1 m KOH, and the key factors were attributed to the dz^2^ orbital‐related re‐distribution of d electrons.^[^ ^251^ ^]^ The synergy between the adjacent Co and Mo atoms co‐existing in a small area, the t‐Co_2_MnN_8_‐2OH active site provided an optimal adsorption/desorption energy toward the ORR intermediates, resulting in high ORR activity.^[^ ^251^ ^]^ In summary, TMAs exhibited outstanding catalytic activity and long‐life stability, providing a new direction for developing high‐performance materials. On the other hand, the reaction mechanism was not well presented in TMAs because of the limited number of TMAs available from the experiments.

In the case of MMAs, HEAs have been reported for the ORR in both alkaline and acidic media.^[^ ^190^, ^203^, ^252^ ^]^ In this context, Cui et al.^[^ ^252^ ^]^ reported an antiperovskite‐typed PdNFe_3_ on the surface of Pd/C (PdNFe_3_@Pd/C) material for the ORR in 0.1 m KOH solution. The as‐obtained PdNFe_3_@Pd/C material exhibited excellent ORR catalytic activity in terms of E_1/2_ (0.91 V vs RHE), mass activity (1.14 A mg^−1^ P_d_ at 0.9 V), and stability (20 000 cycles).^[^ ^252^ ^]^ DFT calculations showed that the compressive strain was responsible for the high ORR activity, which downshifted the d‐band center and weakened the adsorption of oxygen intermediates, reducing the free energy barrier of the ORR rate‐determining step.^[^ ^252^ ^]^ The structure stability and cycling durability were attributed to a stable core in PdNFe_3_@Pd material.^[^ ^252^ ^]^ Huang et al.^[^ ^203^ ^]^ synthesized a series of multi‐metals Pt_4_FeCoCuNi NCs with tailorable degrees of ordering. The catalytic performance is related directly to crystal and electronic structures. They reported that increasing the degree of ordering enhances the catalytic activity toward the ORR in an alkaline solution.^[^ ^203^ ^]^ The highly ordered Pt_4_FeCoCuNi obtained the E_1/2_ of 0.943 V (vs RHE), which is significantly higher than that of partially ordered Pt_4_FeCoCuNi (0.927 V vs RHE), disordered Pt_4_FeCoCuNi (0.91 V vs RHE), and commercial Pt/C (0.861 V vs RHE).^[^ ^203^ ^]^ The promoted ORR activity was attributed to the modified electronic configuration (i.e., downshifted *d*‐band center) after mixing with FeCoNiCu.^[^ ^203^ ^]^ Li et al.^[^ ^190^ ^]^ also fabricated high‐entropy alloy NCs (HEA‐NCs‐14; PtZrNbFeCuTaMoHfBiWZnSnPdNi:14) using a step‐alloying method. Benefiting from the large range of active sites, HEA‐NCs‐14 displayed outstanding electrocatalytic performance (E_1/2_ of 0.86 V vs RHE) toward the ORR in an alkaline solution.^[^ ^190^ ^]^ Furthermore, the polarization curve of HEA‐NPs‐(14) showed that the current density was maintained, even after 5000 cycles, indicating its excellent durability.^[^ ^190^ ^]^ The constructed HEA‐NPs‐(14) displayed exceptional performance for the ORR due to the modified absorption of oxygen intermediates, the rapid electron transport in the distorted lattice, and the high entropy nature.^[^ ^190^ ^]^ The MMA‐based HEAs provided an additional pathway to maximize through fabricating favorable geometric structures. They delivered a well‐defined platform to understand the complex structure–activity relationships of MMA‐based HEA materials. Despite the great advances, the construction controlled by the morphology and composition of MMA‐based HEA structures is still a great challenge because of the enormous differences of constituent metals in reduction potential, atomic size, and electronic configuration.

### 6.5 Fuel Cells

Proton‐exchange‐membrane fuel cells (PEMFCs) and anion‐exchange‐membrane fuel cells (AEMFCs) are the most promising electrochemical‐generating systems because of their high conversion efficiency, high power density, low emission of greenhouse gases, and energy supply.^[^ ^258^, ^259^, ^260^, ^261^, ^262^ ^]^ These cells convert chemical energy directly into electricity, which is based on two main reactions: the hydrogen or fuel oxidation reactions (HOR, MOR, and EOR; anode‐side) and the oxygen reduction reaction (ORR; cathode‐side).^[^ ^258^, ^259^, ^260^, ^261^, ^262^ ^]^ Compared to HOR, the ORR has slower kinetics and a high over‐potential, which is a more critical issue for the overall performance of PEMFCs and AEMFCs.^[^ ^258^, ^259^, ^260^, ^261^, ^262^ ^]^ Currently, research has focused mostly on decreasing the Pt content in cathodes through material design based on the usage of less Pt‐group metals.^[^ ^258^, ^259^, ^260^, ^261^, ^262^, ^263^ ^]^ In this regard, the strategies applied to promote the ORR of cathode materials are based on SMAs/DMAs/TMAs/MMAs.^[^ ^264^, ^265^, ^266^, ^267^ ^]^ This section discusses the recent advances in cathode materials based on SMAs/DMAs/TMAs/MMAs, including HEAs for developing PEMFCs and AEMFCs.^[^ ^265^, ^266^, ^267^, ^268^, ^269^, ^270^, ^271^, ^272^, ^273^, ^274^, ^275^, ^276^, ^277^, ^278^ ^]^ Inspired by the great achievements of SMAs, Kang et al.^[^ ^268^ ^]^ synthesized Fe–N–C from a less stable D1 structure (O–FeN_4_C_12_) to a more stable D2 structure (FeN_4_C_10_) for PEMFCs. Under optimized conditions, Fe–N–C SMA shows outstanding operation durability in H_2_–O_2_ PEMFCs (more than 80% performance maintained after 30 h) and delivered a power density of 0.687 W cm^−2^.^[^ ^268^ ^]^ The power density decreased in non‐coordinating N active sites upon pyrolysis above 1100 °C, which reduced H_2_O_2_ production and alleviated the risk of single‐atom Fe demetallation, resulting in excellent ORR activity and PEMFC performance.^[^ ^268^ ^]^ In 2023, Feng et al.^[^ ^235^ ^]^ prepared dense FeN_4_ motifs on porous carbon with highly curved surfaces (denoted as FeN_4_‐hcC) and combined them into a membrane electrode assembly. This MEA‐based PEMFC delivered a maximum peak power density of 0.592 W cm^−2^ and exhibited operation stability over 30 000 cycles in the presence of H_2_‐air.^[^ ^235^ ^]^ They reported that the curved carbon support modified the Fe d‐band centers, lowering free energy barriers for oxygen intermediates.^[^ ^235^ ^]^ The same year, Liu et al.^[^ ^269^ ^]^ developed ZIF‐based Fe–N–C materials with a manageable N‐doped carbon for H_2_–O_2_ PEMFCs. This device with highly active Fe–N–C material delivered a power density of >1 W cm^−2^ and maintained stability even after 40 h.^[^ ^269^ ^]^ The high durability was attributed to the mitigation of carbon corrosion and the protection of Fe via a N–C coating.^[^ ^269^ ^]^ Feng et al.^[^ ^270^ ^]^ dispersed Pt atomically on an Fe–N–C aerogel (Pt–metal/Fe–N–C) was successfully fabricated and used in H_2_–Air PEMFCs. The tiny‐Pt‐loaded PEMFC exhibited a power density of 0.83 W cm^−2^ with a low voltage loss (8 mV@0.80 A cm^−2^) and no change in the ESA after 60 000 cycles.^[^ ^270^ ^]^ The long‐term stability and power density of the Pt–Fe/Fe–N–C in the fuel cell was attributed to rapid oxygen mass transport and the adsorption energy of *O on the Fe–N–C larger than pure carbon; therefore, less carbon corrosion occurred.^[^ ^270^ ^]^

Berthon‐Fabry et al.^[^ ^271^ ^]^ developed a series of Fe–N–C aerogel materials by regulating the nitrogen content with melamine molecules. ^57^Fe Mössbauer spectroscopy revealed most of the O–Fe(III)N_4_C_12_ structure of the active sites and played an essential role in improving the performance of PEMFCs.^[^ ^271^ ^]^ Under optimized conditions, such a Fe–N–C aerogel material exhibited a high current density and power density of 104 mA cm^−2^ (at 0.8 V in PEMFC) and 0.549 W cm^−2^ (H_2_–O_2_ PEMFC), respectively.^[^ ^271^ ^]^ They reported that the ORR activity trends are similar to PEMFCs.^[^ ^271^ ^]^ Active Fe atoms are bound by N‐pyrrole major activity sites for such high ORR/PEMFC performance.^[^ ^271^ ^]^ Although SMAs showed excellent activity, the power density and durability/stability were unsuitable for practical use. Therefore, DMAs might be more active and stable materials for improving the ORR/PEMFC performance.

In this context, Wu et al. fabricated^[^ ^272^ ^]^ a Zr/Fe co‐doped M–N–C material to improve the durability of the ORR. More importantly, when used as a cathode material in PEMFC, it lost only 25 and 40% of the voltages after 20 and 100 h stability tests.^[^ ^272^ ^]^ The PEMFC based on Zr/Fe co‐doped M–N–C material provided a power density of 0.72 W cm^–2^ (H_2_–Air PEMFC), while N_2_(N)–Fe–N_2_–Zr–N_2_(O_2_) active sites enhances the intrinsic ORR activity.^[^ ^272^ ^]^ Zr active sites also reduce H_2_O_2_ formation, influencing the fuel cell performance.^[^ ^272^ ^]^ Gao et al.^[^ ^273^ ^]^ reported the synthesis of nickel nitride (Ni_3_N) and zirconium nitride (ZrN) using a plasma process, which showed excellent HOR and ORR activity in alkaline media. They used these materials as an anode electrode and a cathode electrode in AEMFCs based on the HOR and ORR results.^[^ ^273^ ^]^ The AEMFC device produced power densities of 256 mW cm^−2^ (H_2_–O_2_) and 151 mW cm^−2^ (H_2_–Air), which is still far below the commercial catalysts.^[^ ^273^ ^]^

**Figure 27:**
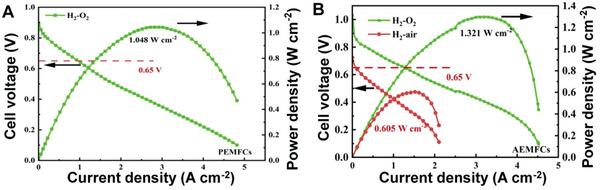
A,B) Polarization and power density plots of PEMFCs and AEMFCs, respectively. Reproduced with permission.^[^ ^275^ ^]^ Copyright 2022 Elsevier B.V.

Shao et al. synthesized high‐density Pt and Fe DMAs anchored in a nitrogen‐doped carbon (called Pt–Fe–N–C)^[^ ^274^ ^]^ There are few Pt–Fe NPs with DMAs.^[^ ^274^ ^]^ The PEMFC based on Pt–Fe–N–C material delivered a power density of 1.08 W cm^–2^ at 2.0 A cm^–2^ (H_2_–O_2_ PEMFC), which is much better than that of Pt/C (1.37 W cm^−2^).^[^ ^274^ ^]^ The morphology and structure of the Pt–Fe–N–C material remained the same even after the 100 000 test cycles, suggesting the protection of Pt‐N_x_ and Fe‐N_x_ (DMAs) coordination structures.^[^ ^274^ ^]^ The high PEMFC performance was attributed to profuse Pt–N_1_C_3_ active sites in Pt–Fe–N–C.^[^ ^274^ ^]^ Cao et al.^[^ ^275^ ^]^ synthesized a Fe–Mn–N–C DMAs with a new local structure of FeN_4_‐MnN_3_ sites, which suppressed the H_2_O_2_ generation and exhibited excellent ORR activity compared to other materials (Fe–N–C and Mn‐N‐C SMAs).^[^ ^275^ ^]^ In addition, Fe–Mn–N–C‐based PEMFC and AEMFC devices provided power densities of 1.048 W cm^−2^ and 1.321 W cm^−2^, respectively (**Figure** **27**A,B)^[^ ^275^ ^]^ Their DFT calculations showed that the Mn–Fe bond regulated the electronic configuration of the Fe–Mn–N–C material. In addition, the co‐adsorption of Fe–Mn DMAs motifs to *OOH eliminated the two‐electron ORR pathway and breaks in the linear relationship between G_OH*_ and G_OOH*_.^[^ ^275^ ^]^ The above results indicated that the DMAs might inspire new studies of the performance of ORR/PEMFCs. Unfortunately, the power density was still below the industrial requirement. Therefore, more work will be needed to develop DMAs for PEMFC/AEMFC.

In 2023, Huang et al.^[^ ^276^ ^]^ developed a platinum–iron–cobalt (denoted as Pt_3_FeCo NSs; TMAs) material with low‐coordination structures for the H_2_–O_2_ PEMFC. Importantly, Pt_3_FeCo NSs used as a cathode material in the PEMFC delivered a high‐power density (1.8 W cm^−2^) and a long‐time stability (188 h).^[^ ^276^ ^]^ They reported that the different coordinated Pt sites on the high‐indexed plane were responsible for the high electronic activities and PEMFC performance.^[^ ^276^ ^]^ Li et al.^[^ ^277^ ^]^ prepared L1_0_Pt_2_CuGa (MMAs) NPs with a unique interaction between Pt and Ga atoms (p–d orbital hybridization), which exhibited an excellent power density (2.60/1.24 W cm^−2^; H_2_–O_2_/Air) and stability (30 000 cycles) in PEMFC.^[^ ^277^ ^]^ The strong covalent bonding interaction between Pt and Ga and the optimized oxygen binding energies at the L1_0_Pt_2_CuGa surface are the main reasons for the higher ORR activity and long‐life durability.^[^ ^277^ ^]^

Similarly, Xia et al. reported the HEAs (PtIrFeCoCu; MMAs) for the ORR and H_2_–O_2_ PEMFCs.^[^ ^278^ ^]^ This material exhibited high ORR mass activity and displayed a superb power density of 1.73 W cm^–2^ with outstanding durability (60 000 cycles and 80 h), verifying the top‐level performance.^[^ ^278^ ^]^ DFT calculations showed that the (001) plane allows minimal activation barriers for the rate‐determining step. In addition, the ideal downshift of the d‐band center was responsible for the better PEM fuel cell performance.^[^ ^278^ ^]^ Overall, TMAs/MMAs exhibit the highest power density, but the TMAs/MMAs are still unsatisfactory for industrial use in H_2_–O_2_/air PEMFCs owing to their unsatisfactory performance in the ORR activity and stability. Thus, exploring SMAs/DMAs/TMAs/MMAs with excellent stability and superior catalytic activity toward the ORR is one of the great topics in fuel cells.

### 6.6 Zn–Air Batteries

The continuous increase in global demand for renewable energy storage and electricity‐powered vehicles makes advanced energy storage devices a major research topic. Among the various battery types, the electrically rechargeable ZABs are considered a promising energy storage device to meet this growing energy demand because of their high energy density (theoretical energy density: 1353 W h kg^−1^ excluding oxygen), outstanding stability, safety, and low cost. On the other hand, the use of this battery in the commercial and industrial sectors is limited by the slow electrokinetics of the ORR and OER in their discharge/charge processes. Thus, highly efficient and stable bifunctional oxygen electrocatalysts are urgently needed to ensure high‐performance rechargeable ZABs. In this issue, SMAs/DMAs/TMAs/MMAs have attracted considerable attention for developing ZABs because of their maximized atom dispersion and utilization efficiency of noble/non‐noble metals (**Table** **10**).

**Table 10:** Zn‐air battery performance by SMA/DMA/TMA/MMA‐based materials.

Materials and types	Maximal power density [mW cm^−2^]	Specific capacity [mAh g^−1^]	Durability [h]	Reference
FeSAs/NC (SMAs)	306.1	746.9 @10 mA cm^−2^	315	[279]
Fe_1_‐N‐C (SMAs)	205.0	815@20 mA cm^−2^	400	[247]
NiO_x_@FePc‐PI/KB (SMAs)	176.9	814.4@2 mA cm^−2^	13.3	[280]
Ce SAs/PSNC (SMAs)	212	783@10 mA cm^−2^	300	[281]
SnN_3_O‐50 (SMAs)	173.5	802@5 mA cm^−2^	200	[233]
Cu‐SACs (SMAs)	165	915@5 mA cm^−2^	1100	[246]
S_1_‐Cr_1_N_4_‐C (SMAs)	153	834@5 mA cm^−2^	100	[238]
Mo‐O_2_S_2_‐C (SMAs)	197	771@10 mA cm^−2^	50	[282]
P/Fe–N–C (SMAs)	269	785@20 mA cm^−2^	192	[283]
FeN4S1/CoN4S1 (DMAs)	152.8	782.1@20 mA cm^−2^	120	[131]
Ni,Fe‐DSAs/NCs (DMAs)	217.5	780.1@40 mA cm^−2^	500	[284]
FeCo‐NC (DMAs)	133	747@2 mA cm^−2^	400	[285]
Cu‐Co/NC (DMAs)	296	752.2@10 mA cm^−2^	510	[219]
Fe‐Se/NC (DMAs)	135	764@5 mA cm^−2^	200	[286]
Fe/Co–N/S_x_–C (DMAs)	138	763.2@20 mA cm^−2^	16.7	[223]
FeCu‐SAC (DMAs)	201.4	827.7@20 mA cm^−2^	NA	[249]
FePtNC (DMAs)	191.8	713@10 mA cm^−2^	64	[127]
FeCo‐NCH (DMAs)	414.5	809.2@20 mA cm^−2^	100	[287]
CuCo‐NC/NPs (DMAs)	170	806 @10 mA cm^−2^	540	[288]
A‐SAC(Fe, Ni, Zn)/NC (TMAs)	300	809@50 mA cm^−2^	358	[289]
CoN_4_‐C‐NiN_4_‐C‐FeN_4_ (TMAs)	132.1	728.3@5 mA cm^−2^	260	[18]
Fe–Co–Ni@NDC (TMAs)	247	894@10 mA cm^−2^	100	[225]
N/P‐Cu_0.1_Co_0.3_Mn_0.6_O_2_/CNTs (TMAs/MMAs)	108.1	–	200	[290]
Fe_4_Co_1_Ni_2_@hNCT (TMAs/MMAs)	125.6	743.6@5 mA cm^−2^	110	[291]
Co_3_Mo_3_N (TMAs/MMAs)	‐	850.0 @ 10 mA cm^−2^	260	[292]

Individually, the recent progress on ZABs is reported.^[^ ^236^, ^287^, ^293^, ^294^, ^295^, ^296^, ^297^, ^298^, ^299^, ^300^, ^301^ ^]^ Chen et al.^[^ ^236^ ^]^ fabricated Co single atoms embedded on N‐doped carbon (denoted as Co‐SAs/N–C/rGO) as an ORR catalyst for liquid ZAB in 6.0 m KOH–0.2 m Zn(OAc)_2_ solution. The liquid ZAB assembled with Co‐SAs/N–C/rGO material delivered the open‐circuit voltage (OCV) of 1.52 V and a discharging specific capacity of 671.94 mA h g^−1^, which is far better than the commercial catalysts‐based device (Pt/C + RuO_2_ (1.49 V and 657.32 mA h g^−1^).^[^ ^236^ ^]^ They fabricated a flexible solid‐state ZAB using Co‐SAs/N–C/rGO (FSZAB@Co‐SAs/N–C/rGO) as an air cathode material.^[^ ^236^ ^]^ This flexible device exhibited a high OCV (1.36 V), excellent power density (74.61 mW cm^−2^), good mechanical flexibility (LED device lighted at 90°, 135°, 180°, and returned to 0°), long‐term stability (26.31 hours), highlighting the potential of this material for industrial applications.^[^ ^236^ ^]^ They reported that the CoN_4_ structure is the leading active site for high‐performance ZABs.

Among the non‐precious 3*d* metals, Fe single atom‐based materials have attracted increasing attention as promising ORR catalysts and easily adjusted coordination environments.^[^ ^293^, ^294^ ^]^ In this context, Wu et al.^[^ ^293^ ^]^ synthesized N,O symmetrical double‐bonded Fe single atoms (FeSAs) anchored in a graphene framework (Fe–N,O/G) for the ZAB. The ZAB assembled with Fe–N,O/G as the cathode catalyst exhibited a maximum power density of 164.7 mW cm^−2^ and a durability of >150 h at 20 mA cm^−2^.^[^ ^293^ ^]^ The symmetrical N,O double bonds modulated the electronic structure of FeSAs and the strength of adsorption of the atomic Fe–N_x_ sites toward the intermediate, resulting in high catalytic efficiency.^[^ ^293^ ^]^ Müllen et al.^[^ ^217^ ^]^ improved the Fe–N–C activity by introducing phosphorus atoms into the second coordination shell (called P/Fe–N–C). First‐principles calculations showed that the P increased the catalytic activity by balancing the intermediate (*OOH/*O) adsorption at the FeN_4_ active sites.^[^ ^217^ ^]^ The constructed ZAB based on the P/Fe–N–C catalytic air cathode achieved a maximum power density of 269 mW cm^−2^ and a discharge durability of 192 h (negligible decay after 190 h), indicating good catalytic activity and stability.^[^ ^217^ ^]^ Similarly, Li et al.^[^ ^294^ ^]^ reported the Fe SAs/NC deposited on nickel foam as an air cathode for ZAB. The FeSAs/NC‐based ZAB displayed a good OCV (1.46 V), a high peak power density (306.1 mW cm^−2^), a large specific capacity (746.9 mAh g^−1^), and excellent stability (no noticeable loss before 315 h; 630 discharge/charge cycles).^[^ ^294^ ^]^ According to the theoretical results, the Fe_1_N_4_O_1_ species in FeSAs/NC optimized the charge redistribution near the Fe center and tuned the binding of ORR intermediates, improving the ZAB performance.^[^ ^294^ ^]^ Like FeSAs, the MoSAs (i.e., Mo‐O_2_S_2_‐C moieties) provided more active sites, facilitated mass transfer, and regulated the adsorption energies of oxygenated intermediates on the Mo sites, resulting in high catalytic efficiency.^[^ ^282^, ^295^ ^]^

More recently, Kim et al.^[^ ^295^ ^]^ developed the ZAB using MoSAs anchored on a N‐doped carbon framework. The ZAB delivered a high maximal power density and a long service life of 376.4 mW cm^−2^ and 630 h, respectively.^[^ ^295^ ^]^ This rechargeable ZAB is also worked at low to high temperatures (−20 to 80 °C) under mechanical deformation. First‐principles calculations suggested that the Mo atom motifs in carbon supported via 2 pyrrolic‐N/2 pyridinic‐N sites are the main active sites for enhancing the ZAB performance.^[^ ^295^ ^]^ Cu single atoms (CuSAs) confined in a carbon framework exhibited promising activity and stability for ZABs. ^[^ ^243^, ^244^, ^296^ ^]^

In this regard, Yuan et al.^[^ ^243^ ^]^ designed the CuSAs and modulated their properties by P‐doping as a secondary heteroatom (abbreviated as Cu SAs/P). The CuSAC/P‐based ZAB exhibited a similar OCV to the commercial material (Pt/C), while the specific capacity of the Cu SAC/P‐based ZAB (766 mAhg_Zn_^−1^) was higher than that of Pt/C‐based ZAB (725 mAhg_Zn_^−1^).^[^ ^243^ ^]^ In addition, SAC/P‐700‐based ZAB maintained outstanding coulombic efficiency (93.4%), suggesting its potential application in energy storage devices.^[^ ^243^ ^]^ Theoretical studies suggested that secondary heteroatomic P effectively adjusted the Cu d‐band center and increased the adsorption of ORR intermediates, enhancing the ZAB performance.^[^ ^243^ ^]^ Cerium single atoms (CeSAs) showed high catalytic activity and stability toward ZABs.^[^ ^281^, ^297^ ^]^

In 2023, Du et al.^[^ ^281^ ^]^ reported CeSAs anchored on a P, S, and N co‐doped carbon substrate (called Ce SAs/PSNC) for ZABs. The Ce SAs/PSNC‐based ZAB exhibited a high OCV (1.49 V) and a peak discharge power density of 212 mW cm^−2^.^[^ ^281^ ^]^ They also fabricated the flexible electronic device using the Ce SAs/PSNC material.^[^ ^281^ ^]^ Theoretical calculations showed that incorporating S/P motifs modified the electronic structure of CeSAs, promoted the electroactivity of the CeSAs, and increased the transfer of electrons within the Ce SAs/PSNC, improving the catalytic properties.^[^ ^281^ ^]^

Zhang et al.^[^ ^238^ ^]^ designed and synthesized a chromium (Cr)‐N_4_ catalyst for ZABs containing S_1_‐Cr_1_N_4_ axial bonds formed by extraterrestrial S (called S_1_‐Cr_1_N_4_‐C). The S_1_‐Cr_1_N_4_‐C‐ based ZAB delivered a high specific capacity of 834 mAh g^−1^ and excellent cycling stability over 100 h.^[^ ^238^ ^]^ Theoretical analysis suggested that the axial S could regulate the Cr *d*‐band center (most positive *d*‐band center), and Cr‐N_4_ active sites promoted electron transport during the ORR, boosting the performance of ZABs.^[^ ^238^ ^]^ Zhao et al.^[^ ^233^ ^]^ reported that the SnN_3_O configuration is very stable in both acidic and alkaline media. Therefore, this material was used for fuel cells and ZAB. In the case of ZAB, the maximum power density of 173.5 mW cm^−2^ was reached.^[^ ^233^ ^]^ They reported that the N/O coordination of Sn localizes the 5*p* electrons, leading to strong coupling with the p orbitals of oxygen.^[^ ^233^ ^]^ At the same time, defects are modulated in the adsorption of oxygenated intermediates, resulting in significant improvement in the performance of the ZAB.^[^ ^233^ ^]^ As discussed above, several experimental and theoretical studies reported that 2D materials supported by SMAs exhibited promising results for improving the ZAB performance. On the other hand, little progress has been made under acidic/alkaline conditions, and the performance of ZAB is still unsatisfactory. Therefore, ZAB based on SMAs should be improved in terms of power density, current density, and stability.

Several researchers reported that the 2D materials supported by DMAs improved the performance of ZABs because of their further modification in the metal *d*‐band center.^[^ ^119^, ^127^, ^284^, ^286^, ^287^, ^298^, ^299^, ^300^ ^]^ The double active sites drastically increased the intrinsic activity drastically, improving the electrocatalyst efficiency.^[^ ^119^, ^219^, ^284^, ^286^, ^287^, ^298^, ^299^, ^300^ ^]^

Qiu et al.^[^ ^119^ ^]^ reported Janus‐distributive FeN_4_ and NiN_4_ motifs (DMAs) anchored in sulfur‐doped carbon hollow spheres (FeN_4_‐SC‐NiN_4_) for ZAB applications. A ZAB with FeN_4_‐SC‐NiN_4_ material displayed an initial potential of 2.05 V and 0.90 V for charge and discharge, respectively, achieving a low charge‐discharge voltage difference of 1.15 V @ 5 mA cm^−2^ with good stability (small change observed after 200 cycles test).^[^ ^119^ ^]^ They reported that S reduced the charges of FeN_4_ sites and decreased the *d* band center of the NiN_4_ sites, boosting the ZAB performance.

Chen et al.^[^ ^298^ ^]^ also reported Zn and Fe DMAs implanted in nitrogen‐doped carbon for ZAB. This DMA (Zn/Fe‐NC) was obtained by pyrolysis.^[^ ^298^ ^]^ The Zn/Fe‐NC‐based ZAB exhibited an outstanding peak power density (186 mW cm^−2^) and specific capacity (815 mAh g^−1^), highlighting potential industrial applications.^[^ ^298^ ^]^ The Fe d‐band center was downshifted and affected by Zn, alleviating the OH* adsorption intermediates and increasing the catalytic efficiency.^[^ ^298^ ^]^ Wang et al.^[^ ^286^ ^]^ synthesized an Fe−Se atom pair embedded on N‐doped carbon (Fe−Se/NC) for ZAB. The solid‐state rechargeable ZAB with Fe−Se/NC delivered a durable charge/discharge of 200 h at room temperature.^[^ ^286^ ^]^ Interestingly, this device also operated at extremely low temperatures (−40 °C).^[^ ^286^ ^]^ According to the theoretical results, asymmetrically bonded Fe−Se active sites can modify the electronic structure and adsorption/desorption strength of the active site towards oxygenated intermediates, resulting in improved catalytic performance.^[^ ^286^ ^]^

Fan et al.^[^ ^127^ ^]^ synthesized the atomically dispersed Fe–Pt DMAs anchored in a N‐doped graphene framework (Fe–PtNC). This material was prepared via a polymerization–pyrolysis process.^[^ ^127^ ^]^ ZAB, with a Fe–PtNC material, displayed a good specific energy density of 713 mA h g^−1^ and a peak power density of 191.83 mW cm^−2^.^[^ ^127^ ^]^ They reported that the Fe works as an active center, and the neighboring Pt sites modulated the Fe‐site electronic configuration and lowered the potential barriers during the ORR process.^[^ ^127^ ^]^ Hu et al.^[^ ^287^ ^]^ prepared Fe/Co‐Nx DMAs with high mass loadings for the ZAB. The quantity of DMAs was ≈7.9 wt%, as determined by inductively coupled plasma‐optical emission spectroscopy (ICP‐OES).^[^ ^287^ ^]^ The Fe/Co‐Nx‐based ZAB delivered an OCV, specific capacity, and maximum power density of 1.45 V, 809.2 mAh g_Zn_^−1^, and 414.5 mW cm^−2^, respectively.^[^ ^287^ ^]^ These results indicated that a high DMA loading improves the ZAB performance. In 2023, Li et al.^[^ ^219^ ^]^ reported the Cu–Co DMAs implanted on nitrogen‐doped carbon matrix (Cu‐Co/NC) for ZAB. Many active sites are achieved in these DMAs.^[^ ^219^ ^]^ Based on the Cu–Co/NC material, the homemade ZAB delivered a peak power density of 295.9 mW cm^−2^ and a long‐time durability of 510 h, indicating it to be a promising material for the ZAB.^[^ ^219^ ^]^ DFT calculations showed that the asymmetrically coordinated Cu−Co sites optimize the adsorption/desorption process between the active sites and the oxygen intermediates, thereby boosting the ORR kinetics.^[^ ^219^ ^]^

Lou et al.^[^ ^299^ ^]^ prepared atomically dispersed Cu and Zn sites (DMAs) embedded on N,P‐codoped carbon fibers (called Cu/Zn‐N/P‐CFs) for the ZAB. The Cu/Zn‐N/P‐CFs‐based battery had a life span of over 630 h and a good rate capability (voltage hysteresis is increased with different current densities).^[^ ^299^ ^]^ Lin et al.^[^ ^300^ ^]^ conducted a theoretical study based on Ru/Fe and Ru/Co DMA models. They reported that Ru/Fe DMAs enhance the catalytic efficiency and the activities of the Ru sites. A rechargeable ZAB with RuFe–N–C material delivered an OCV of 1.52 V, a larger energy density of 916.1 Wh kg_Zn_^−1^, a maximum power density of 139.9 mW cm^−2^, and superior long‐term cycling stability after 200 h.^[^ ^300^ ^]^ Based on the above studies, new types of DMA materials can be designed for the ZAB.

TMAs allow reciprocal tailoring of the electronic structures of each metal moiety, thereby breaking activity and stability limitations for the host and guest metals.^[^ ^289^, ^290^, ^292^ ^]^ In this context, Cai et al.^[^ ^290^ ^]^ incorporated Cu, N, and P into the Co_0.4_Mn_0.6_O_2_ nanosheets, which activated the Co/Mn catalytic sites. Using N/P‐Cu_0.1_Co_0.3_Mn_0.6_O_2_/CNTs material for ZAB, the authors achieved an excellent OCV (1.46 V), maximum power density (108.1 mW cm^−2^), small voltage gap, and good stability (200 h).^[^ ^290^ ^]^ They reported that the dopants (Cu, P, and N atoms) largely modified the electronic configuration of the catalytic sites, improving the ZAB performance.^[^ ^290^ ^]^ Li et al.^[^ ^289^ ^]^ designed a Fe–Ni–Zn TMA embedded in a N‐doped carbon framework (called A‐SAC(Fe, Ni, Zn)/NC), which was synthesized by impregnation, pyrolysis, and a NH_3_ heating treatment. The ZAB with A‐SAC(Fe, Ni, Zn)/NC material had excellent specific capacity (809 mAh g^−1^ is close to the theoretical capacity of 820 mAh/g), a good power density (300 mW cm^−2^), and ultralong cycling stability (2150 cycles, 358.3 h).^[^ ^289^ ^]^ Furthermore, the all‐solid‐state ZAB displayed a remarkable peak power density of 64.5 mW cm^−2^ and cycling durability of 25 h, indicating the potential of this material in a ZAB.^[^ ^289^ ^]^

The adsorption/desorption behavior toward oxygenated intermediates could be tailored owing to the co‐existence of more than three different types of SMAs in the adjacent sites (called MMAs), leading to enhanced catalytic efficiency.^[^ ^292^ ^]^ Yang et al.^[^ ^292^ ^]^ reported a Co_3_Mo_3_N (MMAs) material for the ZAB. The integration of Co_3_Mo_3_N in the ZAB delivered an excellent specific capacity of 850 mA h g_Zn_^−1^ at 10 mA cm^−2^ and superior stability of 260 h, suggesting that Co_3_Mo_3_N can be used as a cathode material for the ZAB.^[^ ^292^ ^]^ The band energy was modified through changes in the valence states, improving the catalytic activity.^[^ ^292^ ^]^ SMA/DMA/TMA/MMA‐based materials could improve the ZAB performance but at a high price. In addition, CO poisoning and material instability during the stability test were the main obstacles to ZAB applications. Therefore, the development of durable and cost‐effective configurations and highly tolerant materials for the efficient operation of ZABs is essential.

### 6.7 Hydrogen Peroxide (H_2_O_2_) Production

Hydrogen peroxide (H_2_O_2_) is a relatively green oxidant used widely in several areas, such as industrial bleaching, chemical synthesis, antibacterial agents, and disinfection.^[^ ^301^, ^302^, ^303^, ^304^, ^305^ ^]^ These extensive applications of H_2_O_2_ indicate that the demand is higher for H_2_O_2_. Therefore, large‐scale and rapid production is desirable. H_2_O_2_ generation involves the sequential hydrogenation and oxidation of a 2‐alkylanthraquinone. On the other hand, this procedure is very complex, energy‐intensive, and ecologically harmful. The instability of H_2_O_2_ products also places transport and storage safety at risk. A direct technique has also been introduced to produce H_2_O_2_ through the reaction of hydrogen and oxygen gases. Unfortunately, this process is explosive and requires precious metal catalysts. Thus, an electrochemical method for H_2_O_2_ production using a two‐electron oxygen reduction reaction (2e^–^ ORR) path has attracted extensive attention. The scalable production of H_2_O_2_ is achieved by the rational design of a highly efficient electrocatalyst. Among the promising candidates for the 2e^−^ ORR catalyst, SMA‐based materials have attracted considerable interest because of their tunable electronic configuration and reduced the kinetic barriers for H_2_O_2_ generation, providing very high selectivity and activity for the 2e^–^ ORR.^[^ ^302^, ^303^, ^304^, ^305^, ^306^, ^307^, ^308^, ^309^, ^310^, ^311^, ^312^, ^313^, ^314^, ^315^, ^316^ ^]^

Electrochemical synthesis of H_2_O_2_ in acidic and alkaline media using precious and non‐precious SMA‐embedded heteroatom‐doped carbon sheets has been reported.^[^ ^306^, ^307^, ^308^, ^309^, ^310^, ^311^, ^312^, ^313^, ^314^, ^315^, ^316^, ^317^, ^318^, ^319^, ^320^, ^321^, ^322^, ^323^, ^324^, ^325^, ^326^, ^327^, ^328^, ^329^, ^330^ ^]^ Chorkendorff et al.^[^ ^51^ ^]^ reported the synthesis of atomic Pd active sites (SMAs) on reduced graphene oxide/N‐doped carbon nanospheres (Pd_1_/N‐C) for the generation of electrochemical H_2_O_2_ in an acidic electrolyte. The as‐synthesized material exhibited excellent selectivity (78.9 ± 2.5%) and outstanding cycling “on‐off” stability (10,000 cycles). The high activity, selectivity, and stability were attributed to Pd active sites bonded with six coordinating pyridinic N species.^[^ ^51^ ^]^

The DFT results showed that N‐coordinated Pd atoms regulate the adsorption energy of HOO* intermediates, increasing H_2_O_2_ production.^[^ ^51^ ^]^ Peng et al.^[^ ^307^ ^]^ synthesized Pt single atoms anchored g‐C_3_N_4_ for H_2_O_2_ generation in an alkaline electrolyte. The amount of Pt single atoms is 0.21 wt%, which was confirmed by ICP‐OES analysis.^[^ ^307^ ^]^ Under the optimized conditions, the Pt/CN exhibited high activity (the onset potential: ≈0.81 V vs RHE), selectivity (selectivity of H_2_O_2_: 98%), and durability during H_2_O_2_ production.^[^ ^307^ ^]^ The excellent catalytic performance was attributed to the cooperative effect of Pt single atoms and the strong adsorption and water activation at g‐C_3_N_4_ nanosheets.^[^ ^307^ ^]^ Similarly, Fei et al.^[^ ^308^ ^]^ fabricated single Sb atoms embedded N‐ and S‐codoped carbon fibers (Sb‐NSCF) using the Sb_2_S_3_ template strategy. The Sb‐NSCF exhibited high Faradaic efficiency (97.2%), better mass activity (114.9 A g^−1^ at 0.65 V), and a good production rate of 7.46 mol g^−1^ h^−1^ with insignificant degradation in selectivity after 75 h in alkaline media.^[^ ^308^ ^]^ The DFT calculations showed that S dopants effectively modified the electronic configuration of metal motifs, optimizing the adsorption energy of *OOH intermediates and accelerating H_2_O_2_ production.^[^ ^308^ ^]^

Gao et al.^[^ ^309^ ^]^ reported the Zn‐N_3_O single‐atom sites for H_2_O_2_ production. Owing to the unsymmetrically coordinated structure and high density of single atom Zn, the tuned material Z‐PPy‐600 showed superb H_2_O_2_ selectivity up (100%), small Tafel plot (113 mV dec^−1^) and no loss in activity after 36 h (at 0.60 V vs RHE, pH 13) in alkaline media.^[^ ^309^ ^]^ The theoretical calculations showed that the unsymmetrical‐structure Zn‐N_3_O species regulated the adsorption energy of OOH* intermediates, enhancing the 2e^−^ ORR kinetic rate.^[^ ^309^ ^]^ Sun et al.^[^ ^310^ ^]^ reported that the electron delocalization (lowers the *d*‐band center of the Fe metal) in the Fe−O structure of FeN_2_O_2_ helped facilitate the 2e^−^ ORR step. According to the DFT results, the downshifted d‐band center weakened the intermediate adsorption ability and improved H_2_O_2_ production.^[^ ^310^ ^]^ Dionysiou et al.^[^ ^311^ ^]^ developed an ethanol‐assisted slow nucleation strategy to implant Co‐S_x_ active sites on CuNW (CuNW@tCoS_x_) and demonstrated its ORR selectivity and excellent stability in acidic media. As‐synthesized material showed a low overpotential (0.018 V) with 93% H_2_O_2_ selectivity, and 91% Faraday efficiency at 0.1 V vs RHE.^[^ ^311^ ^]^ Moreover, the Co‐active sites at the surface of CuNW@tCoS_x_ are stable, even undergoing long‐time tests (5 h).^[^ ^311^ ^]^ Theoretical calculations suggested that the *d*‐band energy level of CoS_x_ tuned the Gibbs adsorption free energy of *OOH intermediates, drastically decreasing the H_2_O_2_ potential and enhancing the rate of the 2e^−^ ORR pathway.^[^ ^311^ ^]^ Furthermore, the axially coordinated Co‐N_5_ site fabricated by Chen et al. exhibited efficient electrochemical H_2_O_2_ generation.^[^ ^312^ ^]^ The Co–N_5_C structure motif has good selectivity and a better H_2_O_2_ production rate (6.78 mol peroxide g_catalyst_^−1^ h^−1^) in acidic media.^[^ ^312^ ^]^ The adjusted local electronic structure was attributed to charge transfer between the axial‐N ligand and the Co atom.^[^ ^312^ ^]^ Therefore, the modified binding energy of the *OOH intermediate (protonation of *OOH to yield H_2_O_2_) is responsible for such high activity and selectivity.^[^ ^312^ ^]^

Kim et al.^[^ ^114^ ^]^ reported the Co‐N_5_ motif for oxygen reduction to H_2_O_2_ production in alkaline media. The experimental and theoretical results suggested that the Co–N_5_–O–C structure (Co–N_5_ active sites) optimized the binding energy of the *OOH intermediate, enhancing the mass activity (87.5 A g^−1^ at 0.75 V vs RHE).^[^ ^114^ ^]^ Shi et al.^[^ ^313^ ^]^ probed the role of the edge sites (Co−N_2_ species) in atomic Co−N−C material during H_2_O_2_ production. They reported that edge‐site (reconstructed Co_1_−N_2_‐oxo) configuration provides valence state exchange between Co^(2−δ)+^ and Co^2+^, resulting in 2e^−^ ORR selectivity for H_2_O_2_ production and high Faradaic efficiency (92%) in acidic media.^[^ ^313^ ^]^

Chen et al.^[^ ^314^ ^]^ fabricated a metalloenzyme‐like model that mimicked carbon‐based SMAs for electrocatalytic H_2_O_2_ production in acidic media. The final products were obtained by carbonization processes.^[^ ^314^ ^]^ They found that the CoNOC active configurations exhibited high selectivity (98%) and mass activity (10 A g^−1^@0.60 V vs RHE) toward the H_2_O_2_ production.^[^ ^314^ ^]^ In addition, epoxy‐surrounded CoNOC active sites optimized the binding energy of the *OOH intermediate, accelerating the 2e^−^ ORR kinetic rate.^[^ ^314^ ^]^ More recently, Chen et al. reported industrial‐level current densities.^[^ ^315^ ^]^ They utilized theoretical strategies to recognize SMAs for the 2e^−^ ORR using the *OOH Gibbs adsorption free energy as a descriptor.^[^ ^315^ ^]^ Based on theoretical results, they fabricated the O‐modified Co‐(pyrrolic N)_4_ structure for oxygen reduction to H_2_O_2_ production in alkaline media.^[^ ^315^ ^]^ The as‐fabricated configuration exhibited the industrial‐level current densities up (300 mA cm^−2^) with excellent Faradaic efficiencies (96–100%) for H_2_O_2_ production at 11,527 mmol h^−1^ g_cat_^−1^.^[^ ^315^ ^]^

**Figure 28:**
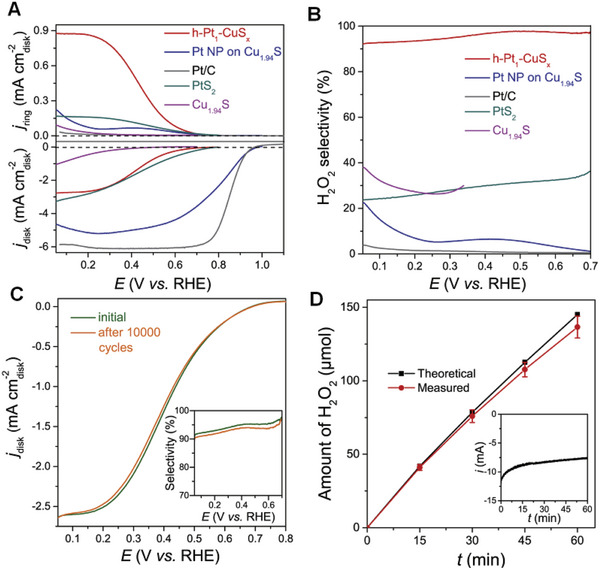
A) Selective oxygen reduction of the materials in an O_2_‐saturated 0.1 m HClO_4_ solution, which was recorded by a rotating ring‐disk electrode (RRDE) voltammograms. B) The calculated H_2_O_2_ selectivity of the materials. C) The LSV of h‐Pt_1_‐CuS_x_ fresh and after stability test (10 000 CV cycles). The inset shows the selectivity of the material. D) H_2_O_2_ concentration as a function of the reaction time at a fixed output voltage (0.05 V). The theoretical line displays the accumulated H_2_O_2_ concentration from the current, and the measured line indicates the H_2_O_2_ amount determined by titration. The inset shows the chronoamperometry plot of the system. Reproduced with permission.^[^ ^317^ ^]^ Copyright 2019 Elsevier Inc.

Although the above studies reported the high potential of SMAs for H_2_O_2_ production, the long‐term stabilities at high current densities are major obstacles because the leaching of SMA moieties reduces the ORR activity with prolonged operation tests. In this regard, DMAs/TMAs/MMAs are the best choice because they prevent the leaching of SMAs during the ORR process.^[^ ^316^, ^317^, ^318^, ^319^, ^320^ ^]^ Li et al.^[^ ^317^ ^]^ synthesized Pt SMA anchored to a hollow CuS_x_ support (called h‐Pt_1_‐CuS_x_; DMAs) for electrochemical H_2_O_2_ production. ICP‐OES confirmed that the actual Pt‐atom amount was ≈24.8 at%.^[^ ^317^ ^]^ The h‐Pt_1_‐CuS_x_ (DMAs) material produced H_2_O_2_ via the reduction of O_2_ with high selectivity (92–96%) at various potentials (0.05–0.7 V vs RHE) in acidic media (**Figure** **28**A,B).^[^ ^317^ ^]^ In addition, h‐Pt_1_‐CuS_x_ exhibited a small change (<2%) in selectivity after 10 000 cycles (Figure 28C).^[^ ^317^ ^]^ More importantly, the h‐Pt_1_‐CuS_x_‐based electrochemical device showed the best stability (Figure 28D) and H_2_O_2_ production ≈546 ± 30 mol kg_cat_^−1^ h^−1^.^[^ ^317^ ^]^

Lin et al. reported atomically dispersed Pt atoms embedded on the surface of AuCu as TMAs for efficient H_2_O_2_ production.^[^ ^318^ ^]^ Under optimized conditions, the AD‐Pt@AuCu material delivered excellent selectivity (91.8%) and stability (5 h) toward H_2_O_2_ production because of its atomic configuration, which optimized the Gibbs adsorption free energy of *OOH intermediate.^[^ ^318^ ^]^ Liang et al.^[^ ^319^ ^]^ developed multi‐metal atoms (MMAs) for the efficient electrocatalytic production of H_2_O_2_ through the 2e^−^ ORR pathway. The Co_x_–Ni MMAs showed better electrochemical performance for the 2e^−^ ORR in terms of H_2_O_2_ production (28.96 mol L^−1^ g_cat_.^−1^ h^−1^) and selectivity (>80%) in a wide potential range (0–0.7 V).^[^ ^319^ ^]^ According to the DFT results, the Co_2_NiN_8_ site structure induced Ni–d orbital filling and regulated *OOH adsorption, promoting the 2e^−^ ORR capability.^[^ ^319^ ^]^ The catalytic activity toward H_2_O_2_ production could be tailored reasonably and accurately by modulating the active sites of the DMAs/TMAs/MMAs. On the other hand, the researchers needed to work on various metal atoms to increase the selectivity, stability, and H_2_O_2_ concentrations.

### 6.8 Carbon Dioxide Reduction Reaction (CO_2_RR)

The electrochemical CO_2_RR to value‐added chemical products (such as methane, ethylene, and ethanol) is one of the most promising approaches for mitigating the atmospheric CO_2_ increase and achieving net‐zero emission energy devices.^[^ ^320^, ^321^, ^322^, ^323^, ^324^, ^325^, ^326^, ^327^, ^328^, ^329^, ^330^, ^331^, ^332^, ^333^, ^334^, ^335^, ^336^, ^337^, ^338^, ^339^, ^340^, ^341^ ^]^ Active, economical, and durable catalysts are needed to obtain efficient CO_2_ conversion and selectivity (achieving the desired product) for industrial applications. Copper (Cu)‐based nanosheets or nanoparticles as electrocatalysts are more popular for the CO_2_RR. On the other hand, reports on these materials are still limited, and their activities are relatively low compared to those containing SMA/DMA/TMA/MMA‐coordinated C, N, S, P, B, and O. Additionally, theoretical studies reported that the coordination environment of Cu substantially changes the adsorption free energy of *CO intermediates, resulting in an alteration of reaction step.^[^ ^342^ ^]^ High coordination number Cu favors producing C_2+_ as a main product with a high Faradaic efficiency (FE) of 82.5%, whereas low coordination number Cu prefers for CH_4_ generation with a FE of 56.7%.^[^ ^342^ ^]^ Therefore, this section discusses SMA/DMA/TMA/MMA‐coordinated C, N, S, P, B, and O for CO_2_RR (**Table** **11**).

**Table 11:** Activities of SMA/DMA/TMA/MMA‐based materials for the CO_2_RR to form chemical products.

Materials	Cathode potential	Current density [mA cm^−2^]	Faradaic efficiency [%]	Chemical products	Stability [h]	Reference
SnO_6_^2−^ (SMAs)	−1.6 V vs (RHE)	34.5	46.5	CH_4_	80	[321]
Co‐N_4_ (SMAs)	−0.9 V vs (RHE)	7.5	59.6	CO	10	[322]
S, V(Bi)‐Bi (SMAs)	−0.95 V vs (RHE)	200	80	Formic acid	2.8	[323]
ZnN_4_S_1_/P‐HC (SMAs)	−0.8 V vs (RHE)	15.8	90	CO	30	[324]
MCs‐(N,O) (SMAs)	−0.55 V vs (RHE)	13.7	94.5	CO	10	[325]
Ni‐N_x_/CB (SMAs)	−0.8 V vs (RHE)	15	90	CO	36	[326]
CuN_3_O/C (SMAs)	−0.9 V vs (RHE)	8.8	90	CO	15	[327]
In_A_/NC (SMAs)	−2.1 V vs (Ag/AgCl)	39.4	72.4	Formate	NA	[328]
In_A_/NC (SMAs)	−2.1 V vs (Ag/AgCl)	39.4	97	CO	24	[328]
CoPc/α‐Co(OH)_2_ (SMAs)	−2.1 V vs (Ag/AgCl)	22.4	98.4	CO	12	[329]
Ni SAs/OMMNC (SMAs)	−0.6 V vs (RHE)	325	99	CO	17	[330]
Fe_1_N_2_O_2_/NC (SMAs)	−0.7 V vs (RHE)	9.5	95	CO	12	[331]
Fe_2_C‐Cs@DC (DMAs)	−0.7 V vs (RHE)	8.53	97.1	CO	24	[343]
Cu–Ag atom pair (DMAs)	−1.2 V vs (RHE)	4.36	21.4	C_2_H_4_	4	[344]
Cu–Pd atom (DMAs)	−0.8 V vs (RHE)	10	74	CO	5	[345]
Cu–Pd atom (DMAs)	−0.5 V vs (RHE)	3.5	79	Formate	–	[345]
Ni_2_NC (DMAs)	−0.37 V vs (RHE)	290	97	CO	30	[335]
N‐bridged Ni‐Mn (DMAs)	−0.6 V vs (RHE)	100	95	CO	10	[118]
Ni–Cu atom‐pairs (DMAs)	−1.1 V vs (RHE)	7.81	99.8	CO	NA	[336]
Ni_2_‐NCNT (DMAs)	−1.1 V vs (RHE)	70	92	CO	52	[337]
Pd_2_ DAC (DMAs)	−0.8 V vs (RHE)	8.5	98.2	CO	12	[338]
Cu–O–Si (DMAs)	−1.27 V vs (RHE)	10.8	72.5	CH_4_	12	[339]
CuMgAl (TMAs)	−0.4 V vs (RHE)	NA	84	CH_3_COOH	1	[340]
FeN/Fe_3_N (TMAs/MMAs)	−0.4 V vs (RHE)	5	98	CO	100	[341]

Different types of isolated metal atoms as active sites for CO_2_RR have been reported.^[^ ^323^, ^325^, ^343^, ^344^, ^345^, ^346^, ^347^, ^348^, ^349^, ^350^, ^351^, ^352^, ^353^, ^354^, ^355^, ^356^ ^]^ Yao et al.^[^ ^354^ ^]^ explored DFT calculations to understand the effect of the configuration and coordination atmosphere on the catalytic CO_2_RR activity of single Cu atoms. Among the different types of Cu–N/C based models, CuN_3_V and CuN_2_V_2_ have good CO_2_RR activity with *COOH (Gibbs free energy; ΔG) as a descriptor.^[^ ^354^ ^]^ Furthermore, CuN_3_V/CuN_2_V_2_ have better CO_2_RR selectivity with an activation energy barrier ≈1.37 eV/1.32 eV (CO_2_RR), which is lower than HER (3.22/4.53 eV).^[^ ^354^ ^]^ The modified catalyst environment and coordination can regulate the nature of the adsorption and the CO_2_RR potential‐determining step (PDS). Moreover, the unsaturated sites are favorable for enhancing the SMA activity.^[^ ^354^ ^]^

Chen et al. fabricated single Cu atoms with different asymmetric atomic interfaces and explored their catalytic performance.^[^ ^327^ ^]^ The CuN_3_O/C exhibited a large turnover frequency (TOF) up to 2782.6 h^−1^ (−0.9 V vs RHE) and high selectivity with a Faradaic efficiency of 96% at −0.8 V vs RHE, whereas CuCO_3_/C structure showed poor selectivity with a Faradaic efficiency of 20% at −0.5 V vs RHE.^[^ ^327^ ^]^ The theoretical results showed that the CuN_3_O motif required a small Δ*G* in the rate‐determining step (RDS) of CO desorption, leading to the efficient electrocatalytic reduction of CO_2_.^[^ ^327^ ^]^ Li et al.^[^ ^355^ ^]^ explored and synthesized the single Ni atom‐embedded N‐doped carbon nanotubes (Ni‐N/NCNT) to boost the CO_2_RR. The Ni‐N/NCNT (SMAs) obtained showed superior CO_2_RR performance with a Faradaic efficiency of 96.73% (at 2.1 V) for CO production.^[^ ^355^ ^]^ High long‐term stability (at −100 mA cm^−2^ for over 60 h) was also maintained using the Ni‐N/NCNT material.^[^ ^355^ ^]^ They reported that Ni‐N/NCNT material easily absorbed the CO_2_, leading to CO_2_ enrichment, which is useful for CO_2_ activation and reduction.^[^ ^355^ ^]^

Chen et al.^[^ ^322^ ^]^ reported the DFT calculations for porphyrin porous organic layers (PPOLs) with various metal centers (M = Fe, Ni, and Co). Among them, the Co‐PPOLs catalyst is most suitable for the CO_2_RR owing to the strong *COOH binding energy and low *CO adsorption energy at the Co‐N_4_ species^[^ ^322^ ^]^ The catalyst of Co‐PPOLs delivered high CO_2_RR selectivity with a peak Faradaic efficiency of more than 90% for CO production.^[^ ^322^ ^]^ In addition, Co‐PPOLs exhibited an industrial‐level current ≈200 mA in a membrane electrode assembly with good durability in CO_2_ electrolysis.^[^ ^322^ ^]^ Liu et al.^[^ ^356^ ^]^ reported a unique catalyst comprising iron as a high‐spin (HS) Fe(III)N_4_ center, in which HS Fe(II)Pc^–^ moieties promoting the current density (*j*_CO_ = 23 mA cm^–2^ at −0.45 V vs. RHE) and selectivity (Faradaic efficiency = 97% at −0.45 V vs RHE) of the CO_2_RR into CO. The DFT results suggested that the electroreduction of the Pc ligand adjusts the center of the d‐band, resulting in moderate binding energy to CO_2_ and increasing the catalytic activity of the CO_2_RR.^[^ ^356^ ^]^

Lu et al.^[^ ^325^ ^]^ anchored atomically dispersed Mn on a mesoporous carbon catalyst, presenting a well‐defined Mn–N_4_ active site and functionalized epoxy groups in the second coordination spheres. Single‐atom Mn bonded with C, N, and O exhibited a Faradaic efficiency for CO production (94.5%) with a current density of 13.7 mA cm^−2^ in the aqueous environment.^[^ ^325^ ^]^ The DFT calculations showed that epoxy groups close to the Mn–N_4_ site modified the electronic configuration of the catalyst and lowered the energy barriers for CO_2_ electroreduction, facilitating the electrocatalytic reduction of CO_2_ to CO.^[^ ^325^ ^]^ Li et al.^[^ ^324^ ^]^ developed SMAs consisting of S‐coordinated Zn‒N_4_ anchored in surrounded phosphorus atoms in the carbon supports (ZnN_4_S_1_/P‐HC), prepared by a pyrolysis process. The ZnN_4_S_1_/P‐HC‐based catalyst showed extraordinary electrocatalytic performance with a high CO Faradaic efficiency of 100% at −0.6 V vs RHE and a Faradaic efficiency over more than 90% over a wide potential range.^[^ ^324^ ^]^ They reported that cooperatively near‐ and long‐range regulation modifies the electronic structure of single Zn atoms, leading to excellent performance for the CO_2_RR.^[^ ^324^ ^]^

Thus far, most SMAs can reduce the CO_2_ gas,^[^ ^322^, ^324^, ^325^, ^327^, ^353^, ^354^, ^355^, ^356^ ^]^ but high overpotentials are required to activate CO_2_ on these SMAs for CO production. Therefore, the design and synthesis of DMAs/TMAs/MMAs are required for the CO_2_RR, which are suitable for potential industrial applications. In this context, Lu et al.^[^ ^337^ ^]^ reported dual‐atom Ni_2_ embedded in N‐doped carbon nanotubes (Ni_2_‐NCNT), which was fabricated by the pyrolysis of a mixture of dinuclear Ni complexes with CNTs and dicyandiamide. The dinuclear Ni complex did not migrate during heating and formed Ni_2_ double atom sites. Under the optimized conditions, the Ni_2_‐NCNT exhibited extraordinary electrocatalytic performance with a high CO Faradaic efficiency (90–97%) over a wide potential window (−0.8 V to −1.2 V vs RHE), a large CO current density of 76.2 mA cm^−2^ at −1.4 V vs RHE, and good stability (52 h), indicating that it is the best material for the CO_2_RR.^[^ ^337^ ^]^ The high activity, selectivity, and stability were attributed to the diatomic Ni_2_–N_3_ sites in Ni_2_‐NCNT that lowered the reaction energy barriers for the CO_2_RR.^[^ ^337^ ^]^ Choo et al.^[^ ^118^ ^]^ synthesized the N‐bridged Ni‐Mn diatomic metal atoms (NiMn DMAs) for the CO_2_RR. A pair of Ni and Mn single atoms was anchored on a N‐doped porous carbon framework by adsorption.^[^ ^118^ ^]^ The NiMn DMAs displayed a high Faradaic efficiency of 98.3%, an industrially relevant current density of 300 mA cm^−2^, and a low overpotential of 0.287 V in a 0.1 m KOH environment.^[^ ^118^ ^]^ According to the theoretical results, the cooperative electronic modification occurred due to the existence of Ni and Mn single atoms in the neighboring Ni‐N_4_ and Mn‐N_4_ species, leading to a decrease in the intermediate energy barriers for CO_2_RR, and boosting the selective production of CO.^[^ ^118^ ^]^

**Figure 29:**
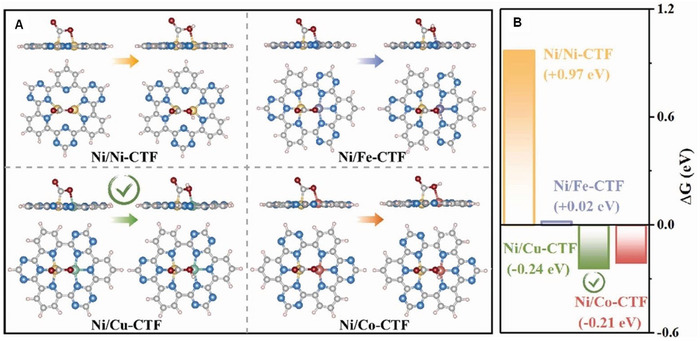
A) Simulated models and B) Free energy diagram of *CO_2_ protonation on Ni/Ni−CTF, Ni/Fe−CTF, Ni/Co−CTF, and Ni/Cu−CTF. Reproduced with permission.^[^ ^336^ ^]^ Copyright 2023 Elsevier B.V.

Song et al. used the Ni–Cu atom pairs for the CO_2_RR.^[^ ^336^ ^]^ They first determined the most favorable atom pair for CO_2_ to CO conversion using DFT calculations.^[^ ^336^ ^]^ Among the different dual‐atom pairs (Ni–Ni, Ni–Fe, Ni–Co, and Ni–Cu), the Ni–Cu pair was the best material for the CO_2_RR because of its facile protonation process and most stable structure (**Figure** **29**A,B).^[^ ^336^ ^]^ Based on the theoretical results, they fabricated DMAs (Ni–Cu) through two‐dip impregnation and annealing.^[^ ^336^ ^]^ The as‐synthesized Ni–Cu catalyst delivered high catalytic activity in terms of Faradaic efficiency (99.82% at −1.1 V vs RHE) and turnover frequency (5116 h^−1^ at −1.1 V vs RHE).^[^ ^336^ ^]^ The theoretical calculations showed that the superior CO_2_RR performance of Ni–Cu can be attributed to the electronic modulation effect of isolated Ni and Cu atoms, rupturing the protonation energy barrier and improving the catalytic activity.^[^ ^336^ ^]^

Han et al.^[^ ^123^ ^]^ designed and fabricated Ni‒Cu dual sites on nitrogen‐doped carbon (Cu/Ni‒NC) using a pregrowth pyrolysis strategy. The Cu/Ni‒NC exhibited extraordinary Faradaic efficiency (99%; electroreduction of CO_2_ into CO) and superb partial current densities for CO (190 ± 11, 225 ± 10, and 489 ± 14 mA cm^−2^ in acidic, neutral, and alkaline solutions, respectively).^[^ ^123^ ^]^ They reported that Cu atoms modified the center of the Ni *d*‐band, accelerating *COOH formation and improving the catalytic efficiency.^[^ ^123^ ^]^ Qiao et al.^[^ ^357^ ^]^ reported a dual‐atom NiCu on the N‐doped carbon (NiCu−NC) material containing a well‐defined NiN_4_−CuN_4_ species for CO_2_RR. Interestingly, they reported that the optimal distance between the NiN_4_ and CuN_4_ active sites (∼5.3 Å) plays a key role in increasing the CO_2_RR and catalytic efficiency.^[^ ^357^ ^]^ Although the above results suggested that the catalytic activities were improved further by DMAs, a recent report suggested that the CO_2_RR is still difficult in the DMA sites because it hinders subsequent C−C coupling, which was confirmed by the Pourbaix diagram.^[^ ^358^ ^]^ Pourbaix's data showed that CO occupied the two metal bridge sites rather than the metal‐top sites.^[^ ^358^ ^]^

In the case of TMAs/MMAs, little literature is available, mainly based on theoretical analysis.^[^ ^340^, ^341^, ^359^, ^360^, ^361^ ^]^ Recently, Lu et al.^[^ ^360^ ^]^ designed triple transition metals (TM = Cu, Fe, and Co) embedded on a graphyne (3TM‐GY) configuration. They reported that TMAs had more cohesive energy (6.91 to 6.98 eV per atom) and a high binding energy (2.28 to 5.95 eV), indicating structural stability in the CO_2_RR. They reported that boosted charge transfer between triple TM atoms and C atoms in 3TM‐GY.^[^ ^360^ ^]^ This unique TMA configuration facilitated the high adsorption energy and CO_2_ activation in the CO_2_RR.^[^ ^360^ ^]^ The 3Cu‐GY structure showed high‐throughput reaction pathways toward HCOOH and CH_4_ in the CO_2_RR. In contrast, 3Fe‐GY/3Co‐GY displayed high‐throughput reaction pathways toward CH_3_OH and CH_4_, suggesting metal atoms can be used to tailor the final products during CO_2_ electroreduction.^[^ ^360^ ^]^

Tonelli et al.^[^ ^340^ ^]^ synthesized a CuMgAl layered double hydroxides (CuMgAl LDH; TMAs) as a carbonaceous gas diffusion membrane for the CO_2_RR. Under optimized conditions, the CuMgAl LDH material displayed an acetic acid yield of 2.0 mmol g_cat_^−1^ h^−1^ at a low potential of −0.4 V vs RHE in a 0.3 m KHCO_3_ environment.^[^ ^340^ ^]^ The evenly dispersed copper species (Cu^0^/Cu_2_O moieties) are responsible for the superior selectivity (100%) of the C_2_ product during CO_2_RR.^[^ ^340^ ^]^ Liu et al.^[^ ^361^ ^]^ synthesized a four‐atom cluster embedded in carbon nitride (Cu_4_‐C_5_N_2_H_2_) structures for the CO_2_RR. They reported that Cu_4_‐C_5_N_2_H_2_ exhibited high selectivity and a low limiting potential (−0.50 V) for ethylene production during the CO_2_RR. By contrast, Cu_2_Zn_2_‐C_5_N_2_H_2_ was an efficient material for ethanol production at −0.46 V.^[^ ^361^ ^]^ Based on the theoretical results, they developed a descriptor, *D_(Cu – Cu)_* × (*D_(N – N)_* – 2 × R*_N_*), which is related to the characteristics of the substrate, support metal, and limiting potential.^[^ ^361^ ^]^

Sun et al.^[^ ^341^ ^]^ synthesized the FeN/Fe_3_N structure for the CO_2_RR. FeN/Fe_3_N exhibited high catalytic performance with a high CO Faradaic efficiency of 98% and excellent stability of 100 h in a CO_2_‐saturated 0.5 m KHCO_3_ solution.^[^ ^341^ ^]^ The Fe−N_4_ and Fe−N_2_ species in FeN/Fe_3_N played a major role in the electroreduction of CO_2_ to CO, which regulates the Fe d‐band center.^[^ ^341^ ^]^ TMAs/MMAs have several advantages over SMAs/DMAs, such as maximum adsorption capacity, high catalytic activity, and selectivity with low overpotentials in RDS toward multi‐carbon products. Nevertheless, more experimental and theoretical studies are needed to prove the importance of TMAs/MMAs.

The CO_2_RR behaviors of electrocatalysts are tested in an H‐type cell at neutral‐pH. On the other hand, the CO_2_RR in this H‐type cell suffers from slow reaction kinetics because of the insufficient CO_2_ supply to the cathode side, low current density, and low Faradaic efficiency/energy efficiency. The slow kinetics of the CO_2_RR for industrial applications can be overcome by loading the catalyst on a gas diffusion electrode (GDE) in a flow cell. This can allow the direct supply of CO_2_ to the catalyst‐electrolyte interface and maintain a sufficient CO_2_ concentration at the catalyst surface, which improves mass transport, dramatically accelerates the CO_2_RR kinetics, and enhances the CO_2_RR performance. Furthermore, CO_2_RR in flow cells is performed in highly alkaline solutions, suppressing the competitive HER reactions and improving the CO_2_RR performance at the expense of the HER. Nevertheless, the flow cell also has many serious problems, such as carbonate formation, crossover, GDE flooding, GDE durability, and the use of massive electrolytes. Therefore, this paper suggests a new design of GDEs with good durability that can eliminate flooding and carbonate formation.

### 6.9 Nitrogen Reduction Reaction (NRR)

Like CO_2_ gas, releasing reactive nitrogen (N_2_) gas into the environment has severe and ongoing impacts on the ecosystem, the economy, and human health.^[^ ^362^, ^363^ ^]^ Therefore, transforming N_2_ gas into high‐value fuel is a great challenge. Currently, the Haber–Bosch process is used to convert inert N_2_ gas into NH_3_, which requires harsh reaction conditions (e.g., high temperatures of 400–600 °C and high pressures of 20–40 MPa) for catalytic reactions, resulting in severe energy consumption (≈1% of global anthropogenic energy) and carbon emissions (≈1.4% of global greenhouse gas emissions). Therefore, discover a new synthesis procedure essential for next‐generation NH_3_ gas production to reduce energy usage, cut carbon emissions, and be compatible with renewable energy. In this respect, the electrocatalytic nitrogen reduction reaction (NRR) is one of the most promising strategies because it offers an ecological and more economical scenario. The electrocatalyst is crucial to the performance of electrochemical devices. Hence, SMAs/DMAs/TMAs/MMAs have been evaluated as high‐efficiency electrocatalysts (**Table** **12**). This section discusses recent progress in this area.

**Table 12:** NRR performance of SMA/DMA/TMA/MMA‐based materials.

Materials	Electrolytes	NH_3_ production	Faradaic efficiency [%]	Reference
Mo/VO_2_ (SMAs)	0.05 m H_2_SO_4_	190.1 µg h^−1^ mg_cat._^−1^	32.4	[364]
Sn ADPs (SMAs)	0.1 m Na_2_SO_4_	28.3 µg h^−1^ mg_cat._^−1^	26.8	[365]
NbB_2_ NFs (SMAs)	0.1 m HCl	30.5 µg h^−1^ mg_cat._^−1^	40.2	[366]
Fe–Co active sites (DMAs)	0.1 m Na_2_SO_4_	579.2±27.8 µg h^−1^ mg_cat._^−1^	79.0±3.8	[367]
CNT@C_3_N_4_‐Fe&Cu (DMAs)	0.001 m H_2_SO_4_	9.86 µg h^−1^ mg_cat._^−1^	34.0	[368]
PdCu/NC (DMAs)	0.05 m H_2_SO_4_	69.2 ± 2.5 µg h^−1^ mg_cat._^−1^	24.8±0.8	[369]
VFe/NC (DMAs)	0.1 m KOH	73.44 µg h^−1^ mg_cat._^−1^	43	[126]
NiCoP/CoMoP/Co(Mo_3_Se_4_)_4_ (TMAs)	0.1 m Na_2_SO_4_	24.54 µg h^−1^cm^−2^	23.2	[370]
RuFeCoNiCu (MMAs)	0.1 m KOH	57.1 µg h^−1^ mg_cat._^−1^	38.5	[371]
Fe_2_Mo_6_S_8_ (MMAs)	0.5 m Na_2_SO_4_	70 µg h^−1^ mg_cat._^−1^	12.5	[372]
Fe_2_Mo_3_O_8_/XC‐72 (MMAs)	0.1 m Na_2_SO_4_	30.4 µg h^−1^ mg_cat._^−1^	8.2	[373]

In the case of the SMAs, Lu et al.^[^ ^364^ ^]^ fabricated Mo single atom‐anchored VO_2_ (Mo/VO_2_) for NH_3_ production through the NRR. Mo/VO_2_ was prepared by a hydrothermal process.^[^ ^364^ ^]^ Mo/VO_2_ material delivered an NH_3_ yield and a Faradaic efficiency of 190.1 µgNH_3_ mg_cat_.^−1^ h^−1^ and 32.4%, respectively.^[^ ^364^ ^]^ The high rate of NH_3_ formation and good Faradaic efficiency were attributed to the incorporation of single Mo atoms, which provided electron‐deficient sites, decreasing the energy barrier for protonation and improving the NRR kinetics.^[^ ^364^ ^]^ In parallel, Lin et al.^[^ ^365^ ^]^ reported a novel Sn atomically dispersed protuberance (Sn ADPs) for the NNR to form NH_3_. In Sn ADPs, Sn is bonded to C and O, which prompted the positive charges on the Sn motif to enhance its N_2_ adsorption.^[^ ^365^ ^]^ They reported that Sn ADPs exhibited an excellent NH_3_ yield of 28.3 µg h^−1^ mg_cat_^−1^.^[^ ^365^ ^]^ Electronic localization on Sn ADP reduces the energy barrier (*N_2_→*NNH), resulting in remarkable NRR electrocatalytic activity.^[^ ^365^ ^]^

**Figure 30:**
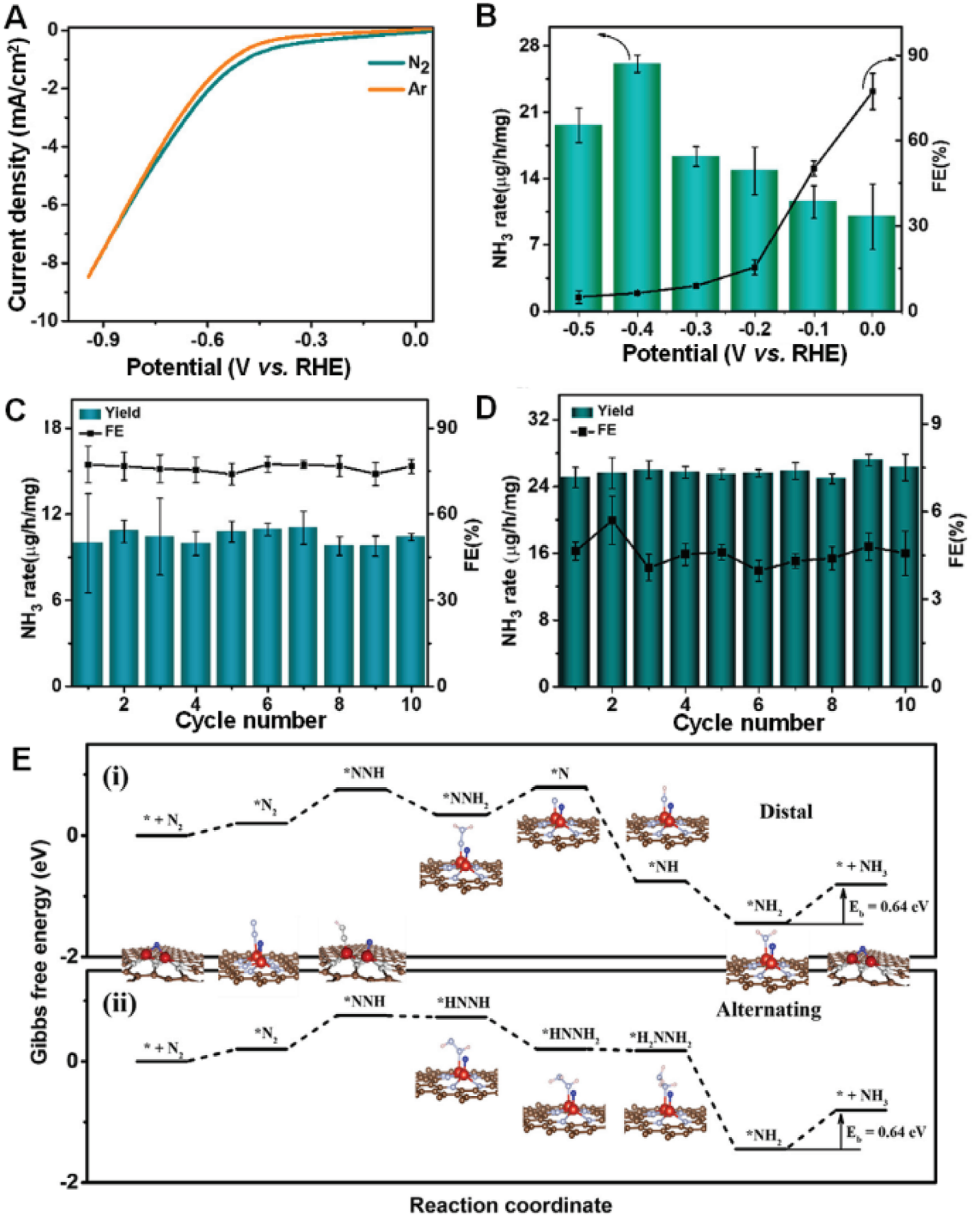
A) Linear sweep voltammetry (LSV) curves of O–V_2_–NC in nitrogen‐ and argon‐saturated electrolytes. B) Electrochemical N_2_ reduction reaction (NRR) obtained at different overpotentials. C,D) Recycling test of O–V_2_–NC at two different potentials (0.0 and −0.4 V vs RHE). E) Gibbs free energy diagram of the NRR on simulated O–V_2_–NC model with a distal step (i) and an alternating step (ii). Reproduced with permission.^[^ ^374^ ^]^ Copyright 2023 American Chemical Society.

Liu et al.^[^ ^366^ ^]^ reported that a boron‐vacancy‐rich diatomic Nb‐B material exhibited tremendous selectivity and stability in the NRR, which produces 30.5 µg h^−1^ mg_cat_^−1^ NH_3_ with a faraday efficiency of 40.2%. The theoretical results indicated that the non‐polar N≡N triple bonds were broken easily at both unsaturated Nb and B atoms, improving the NH_3_ production.^[^ ^366^ ^]^ Based on the above results, the SMAs could provide a high yield and good selectivity for NH_3_. On the other hand, the Faradaic efficiency and yield are yet challenging for the single‐atom center because of the multi‐step reaction during NRR. DMAs are a promising approach to solving the above problem because of the additional modifications of the electronic structure and coordination environment. Lee et al.^[^ ^374^ ^]^ synthesized an oxygen‐bridged vanadium dimer (V–O–V; DMAs) on N‐doped carbon (O–V_2_–NC) structure sites for the NRR. O–V_2_–NC was fabricated through a template‐assisted pyrolysis strategy.^[^ ^374^ ^]^ O–V_2_–NC showed that the current density in a N_2_‐saturated solution was much higher than that in an Ar‐saturated solution over a wide potential range, suggesting the catalytic activity for N_2_ reduction (**Figure** **30**A). The NH_3_ yield and Faradaic efficiency for O–V_2_–NC were increased with the applied voltage and reached a peak at −0.4 V vs RHE with values of 26.12 µg h^−1^ mg^−1^ and 77.2 %, respectively (Figure 30B). This O–V_2_–NC also displayed excellent durability over 10 cycles under 0 V/−0.4V with a negligible loss in yield and faraday efficiency (Figure 30C,D). DFT calculations suggested that the structural change of O–V_2_–NC reduced the energy barriers, and the loop evolution of the configuration played the primary role in the activity during the N_2_ reduction process (Figure 30E). Gao et al.^[^ ^126^ ^]^ suggested that atomically dispersed binary V/Fe implanted on N‐doped carbon (VFe/NC) exhibited excellent activity toward the NRR, which was screened by theoretical calculations. This material was selected from a family of M/Fe combinations (M  =  Sc, Ti, V, Cr, Mn, Co, and Ni).^[^ ^126^ ^]^ Taking advantage of the dual‐active sites, VFe/NC delivers NH_3_ with a production rate of 73.44 µg h^−1^ mg_cat_^−1^ and an ultra‐high faraday efficiency of 43%. Theoretical analysis suggested that the charge polarization of binding N_2_ is the main reason for the high NRR activity, which helps break the N≡N triple bonds.^[^ ^126^ ^]^

Similarly, Kan et al.^[^ ^375^ ^]^ reported that five polyoxometalate(POMs)‐based‐MOFs materials (denoted as Fe_x_Co_y_MOF‐P_2_W_18_) can be used in the electrolytic NRR. From their experimental results, they reported that Fe_x_Co_y_MOF‐P_2_W_18_ delivered an NH_3_ yield of 47.04 µg h^−1^ mg_cat_.^−1^ and a Faradaic efficiency of 31.56%.^[^ ^375^ ^]^ They also reported that P_2_W_18_ helped transfer electrons to MOFs, and Fe and Co lowered the energy barriers for the *N_2_ to *N_2_H step, increasing NH_3_ production.^[^ ^375^ ^]^ In addition to the experimental work, more theoretical studies based on DMAs (i.e., FeMo, TiV, TiCo, VCr, VCo, VNi, and FeRu) were reported for an NRR.^[^ ^376^, ^377^, ^378^ ^]^ Overall, DMA‐based materials are unique candidates for the NRR with substantial activity. On the other hand, the activity/selectivity for an NH_3_ product still does not meet industrial requirements. Therefore, improving the catalytic activity, selectivity, and durability of DMAs‐based materials for industrial uses is essential.

Many studies found that DFT calculation is the best way to screen the TMAs or MMAs for N_2_ reduction.^[^ ^379^, ^380^, ^381^, ^382^ ^]^ For example, Jiang et al.^[^ ^380^ ^]^ utilized the surfaces of graphdiyne and single‐layer graphene to anchor Fe_3_ atoms (denoted as Fe_3_‐GDY/Gra) for the NRR. According to theoretical calculations, the loading of active metal atoms reached 35.0%, which displayed high catalytic activity toward the NRR with a potential of −0.26 V vs RHE.^[^ ^380^ ^]^ More importantly, the active sites of the TMAs (M_3_; M: Mn, Fe, Co, and Ni) exhibited better activity than DMAs/SMAs, which produced more electrons to activate N_2_ and provided weak adsorption to release the desired products.^[^ ^380^ ^]^

Ji et al.^[^ ^381^ ^]^ used the Fe_2_Mo moiety in an Fe_2_Mo–NG material for NH_3_ synthesis under ambient conditions via N_2_ reduction, which was screened through DFT calculations. They also reported that more electrons near the Fermi level activated the triple N≡N bond, improving NRR activity.^[^ ^381^ ^]^ Unlike TMAs, MMAs present electronically diverse cooperativity and various artificial opportunities due to the abundance of metal atom sites. In this context, Zhuang et al.^[^ ^382^ ^]^ used the Ni_0.3_(FeCoCuPd)_0.175_ (HEAs; MMAs) for the NRR. According to theoretical studies, the Ni_0.3_(FeCoCuPd)_0.175_ with exposed {111} facets showed outstanding NRR performance with a small overpotential (0.34 eV) and excellent selectivity (99%). Similarly, Wang et al.^[^ ^371^ ^]^ synthesized the RuFeCoNiCu (HEA; MMAs) for the NRR. They reported that NH_3_ can be produced with a low overpotential. It has an outstanding NH_3_ yield of 57.1 µg h^−1^ mg_cat_^−1^ and an excellent Faradaic efficiency of 38.5%. Furthermore, this material was slightly degraded after 100 h in an alkaline solution, indicating good stability. Wang et al.^[^ ^370^ ^]^ designed and synthesized the NiCoP/CoMoP/Co(Mo_3_Se_4_)_4_ @C/NF material for the NRR. This hybrid exhibited good NRR activity with an NH_3_ yield of 24.54 µg h^− 1^ cm^− 2^ and a Faradaic efficiency of 23.15%. In this hybrid, Co(Mo_3_Se_4_)_4_ acted as the catalytic active sites, and NiCoP/CoMoP accelerated the charge transfer during the NRR.^[^ ^370^ ^]^ Nevertheless, these results were based on theoretical studies. Therefore, more experimental work will be needed to prove the effect of TMAs/MMAs during N_2_ reduction.

### 6.10 Sensors

C, N, S, P, B, and O‐bonded SMAs/DMAs/TMAs/MMAs, a new type of hybrid material, have great potential for sensing applications owing to their accelerated response, high sensitivity, good selectivity, nanoscale miniaturization, reproducible data, and robust sensing properties. The SMA/DMA/TMA/MMA‐coordinated C, N, S, P, B, and O structures have the desired linkers as supports and offer ample opportunities for fine‐tuning their molecular level interactions with the probe analytes. This has assisted scientists in using SMA/DMA/TMA/MMA‐coordinated C, N, S, P, B, and O to detect several analytes ranging from chemicals to biosensors (**Table** **13**).

**Table 13:** Sensing performance by SMA/DMA/TMA/MMA‐based materials.

Materials	Techniques	Analyte	Liner range	LOD	Real samples	Reference
Pd_1_/N‐C	CV	Furazolidone	0.01–50, 50–300 (µm)	3.1 nm	Tap water; Lake water	[383]
Ru_3_/NC	Amperometry	Uric acid (UA)	0.05–1000 (µm)	10 nm	Serum	[384]
PtHPCN‐222/GCE	DPV	Levodopa	0.1–1; 1–130 (µm)	0.003 (µm)	Human blood serum	[385]
Co SAC /SPCE	SWASV	As(III)	0.1–10 (ppb)	0.023 (ppb)	–	[386]
Ru‐Ala‐C_3_N_4_	Chronoamperometry	Dopamine (DA) and UA	0.06−490 for DA; 0.5−2100 for UA	0.02 for DA; 0.17 for UA	Serum	[387]
Fe_3_C@C/Fe‐N‐C	Amperometry	H_2_O_2_	1–6000 (µm)	0.26 (µm)	living cells	[388]
Pt_1_/Ni(OH)_2_/NG	Chronoamperometry	Glucose	0.01–2.18	–	–	[389]
Fe‐SASC/G	Chronoamperometry	H_2_O_2_	10–920 (µm); 920–7020 (µm)	0.2 (µm)	Human lung adenocarcinoma cells A549	[390]
Fe SAs‐N/C	Chronoamperometry	H_2_O_2_	1–54; 54–764; 764–9664	0.34 (µm)	MCF‐7 Cells	[391]
Fe‐SACs/Ti_3_C_2_T_x_	SWV	DA; (vanillylmandelic acid (VMA); homovanillic acid (HVA)	0.001–200 0.010–200 0.020–200 (µm)	1 5 10 (nm)	Human urine; Human serum; PC12 cells	[392]
Cu_1_/C_3_N_4_	Chronoamperometry	H_2_O_2_	–	–	Living rat brain	[32]
NiN_4_‐SACs	–	H_2_S	–	–	Mouse Brain	[393]
Ni SACs/N‐C/PDMS	Amperometry	Nitric oxide	–	1.8 (nm)	–	[31]
Mn‐MoS_2_/PGS	DPV	DA	5 × 10^−5^ to 5 × 10^1^	5 × 10^−5^	Artificial sweat solution; 10% serum	[394]
Fe‐SASC/NW	Amperometry	H_2_O_2_	50 nm–500 mm	46.35 × 10^−9^ m	–	[395]
Pt_1_/Cu@CuO	Amperometry	Glucose	0.01–5.12 (mm)	3.6 (µm)	–	[396]
Pt_1_/Ni_6_Co1LDHs/NG	Amperometry	Glucose	–	–	–	[397]
CuO‐Sn_1_	DPV	DA	0.02–400 (µm)	0.013 (µm)	–	[398]
In_1_‐N‐C	DPV	DA	0–500 (µm)	0.279 (µm)	Serum	[399]
Pt SA‐Ti_3_C_2_T_x_	Gas sensors	Triethylamine (TEA)		14 ppb	–	[400]
Ni‐N_2_O_2_/AB		Nitric oxide	0.3–180 ppb	0.05 ppb	–	[401]
Fe‐N‐C SAE	SGGT	Hg^2+^	30 nm–3 µm	1 (nm)	Drinking water	[402]
Pt‐SnO_2_	–	Triethylamine	–	7 ppb	–	[403]
Ni‐SAC/GCE	DPV	H_2_O_2_	0.02 – 1×10^9^	6.87 ×10^−3^	–	[404]
Pt_1_/ZnO	Gas sensors	Triethylamine	–	–	–	[405]
In_2_O_3_/Pd_atom_	Gas sensors	H_2_S		0.1 ppm	–	[406]
Ag‐LaFeO_3_ @ZnO‐Pt	Gas sensors	Methanol	–	5 ppm	–	[407]
Pt SAs@SnO_2_ NRs@SiC NSs	Gas sensors	Ethanol		500 ppm	–	[408]
Fe‐N_5_ SAC	Amperometry		0.005–500 (µm) 0.01–480 (µm)	0.007 (µm) 0.027 (µm)	–	[409]
SAC‐Ni/H‐SnO_2_ nanorods	–	–	–	0.1 (ppm)	–	[410]
FeN_3_P	SWASV	As(III)	1–11 (ppb)	0.01 (ppb)	–	[411]
SANb‐BCN/GCE	LSV	Nitrobenzene	2–100, 100–600 (µm)	0.70 (µm)	–	[412]
Co (4%)‐N/CNSs	Amperometry	H_2_O_2_	1‒500 500‒1 000 000 (µm)	0.006 (µm)	–	[413]

Note: The limit of detection (LOD) was determined using Equation (4):^[^ ^414^, ^415^, ^416^, ^417^ ^]^

(4) LOD=3S/m

The electroactive surface area (ESA) of the different materials‐modified electrodes can be evaluated using the Randles–Sevcik (Equation 5):^[^ ^414^, ^415^, ^416^, ^417^ ^]^

(5) $$I_{P}=\;2.69*105AD^{1/2}n^{3/2}\nu^{1/2}C$$

The relationship between the peak potential and the sweeping rate was used to calculate K_s_ between the DA and modified electrodes using Laviron (Equations 3 and 4). The formula was as follows:^[^ ^414^, ^415^, ^416^, ^417^ ^]^

(6) $$\begin{array}{ccc} \log\;K_{S} & = & \alpha \log\left(1-\alpha \right)-\left(1-\alpha \right)\log\alpha -\log\left(RT/nF\nu \right)\\ & & -(1-\alpha )\alpha nF\Delta Ep/2.3\;RT \end{array}$$

(7) $$E_{PC}=E^{0}\;+\frac{RT}{\alpha nF}\log\frac{RTk_{0}}{\alpha nF}+\frac{RT}{\alpha nF}\log\nu$$

The quasi‐reversible redox process of levodopa can be calculated using Equation 8:^[^ ^414^, ^415^, ^416^, ^417^ ^]^

(8) $$\frac{dE_{P}}{dpH}=\;-\frac{2.303mRT}{nF}$$

This section discusses SMAs, DMAs, TMAs, and MMAs for use in electrochemical, colorimetric, gas, electrochemiluminescence, photoelectrochemical, nonenzymatic, and enzymatic sensors (biosensors) in detail.^[^ ^393^, ^399^, ^400^, ^401^, ^402^, ^403^, ^404^, ^405^, ^406^, ^407^, ^408^, ^409^, ^410^, ^411^, ^412^, ^413^, ^414^, ^415^, ^416^, ^417^, ^418^, ^419^, ^420^, ^421^, ^422^, ^423^, ^424^, ^425^, ^426^, ^427^, ^428^, ^429^ ^]^ Recently, Lu et al.^[^ ^399^ ^]^ developed a single In atom (SMA) implanted on N‐doped carbon (In_1_−N−C) with an In−N_4_ structure for electrochemical dopamine (DA) sensing. The authors reported that In_1_–N–C is highly selective during DA sensing because of the high adsorption energy of the OH groups and reduced energy barrier related to DA oxidation.^[^ ^399^ ^]^ The In_1_−N−C‐based selective electrochemical sensor for DA detection exhibited an LOD of 279 nm.^[^ ^399^ ^]^ Moreover, ascorbic acid, DA, and uric acid (UA) were detected simultaneously by In_1_−N−C.^[^ ^399^ ^]^ Huo et al.^[^ ^418^ ^]^ proposed an electrochemical sensor for H_2_O_2_ detection with an LOD of 0.61 µm, containing a Fe single‐atom nanozyme (Fe‐SNC) with a Fe‐N_4_ active center. After five cycles of use, the peroxidase‐like and electrocatalytic activities of this sensor remained at 93.1% and 99.8%, respectively. However, the LOD related to the colorimetric and analysis process of H_2_O_2_ (H_2_O_2_ released from MCF‐7 cells) was 1.3 µm.^[^ ^418^ ^]^ When H₂O₂ served as the substrate, the *K*_m_ and V_m_ values of Fe‐SNC were determined to be 9.47 mm and 1.37 × 10⁻⁷ m s^−1^, respectively. These values can be attributed to the presence of polyaniline and doped S atoms, inhibiting the aggregation of Fe atoms and inducing the rearrangement of the coordination structure. Nevertheless, designing SMAs with sufficient sensitivity and selectivity for selectively detecting analytes in complex samples under extremely low concentrations remains a major challenge. Additionally, the biosafety assessment of SMAs is highly recommended before use in biosensors. Therefore, the development of SMAs continues to encounter obstacles.

According to Li et al.,^[^ ^383^ ^]^ increasing the number of active sites can improve the sensing performances of SMAs. The authors synthesized atomically dispersed Pd SAs sites embedded in N‐doped carbon (Pd_1_/N‐C) to promote the catalytic activity toward furazolidone redox, significantly enhancing the LOD of furazolidone.^[^ ^383^ ^]^ They designed DMA, TMA, and MMA sites to improve the LOD further and employed the cooperative effect to enhance the performances of the metal atoms.^[^ ^419^, ^420^ ^]^ Dong et al.^[^ ^419^ ^]^ designed and synthesized Fe–Se DMA sites on N‐doped carbon carriers for the nonenzymatic detection of H_2_O_2_. The Fe–Se DMA‐modified electrode was used to detect H_2_O_2_ over a wide linear range (0.02–13 mm) with good sensitivity (1508.6 µA mm ^−1^ cm^−2^) and a small LOD (11.5 µm).^[^ ^419^ ^]^ Additionally, the sensor was used to detect H_2_O_2_ disinfectant and urine environments. They reported that Fe–Se DMAs boosted catalytic activity during H_2_O_2_ reduction.^[^ ^419^ ^]^ Yun et al.^[^ ^420^ ^]^ reported Cu–Au DMAs anchored on bioinspired carbon (CuAu DMAs/BC) for the selective and sensitive detection of DA in biological and cellular samples. The authors demonstrated that Cu atoms facilitate the detection of DA and Au improves the signal, as confirmed via DFT calculations.^[^ ^420^ ^]^ Furthermore, the Cu–Au DMAs/BC‐based material detected DA in neuronal cells, suggesting that the DMA material has promising real‐time applications in electrochemical biosensors.^[^ ^420^ ^]^

Zhu et al.^[^ ^384^ ^]^ prepared Ru_3_ sites (TMAs) for detecting UA. They reported that Ru_3_ sites have higher catalytic activity toward biomolecule oxidation than Ru_1_ sites.^[^ ^384^ ^]^ The Ru_3_ sites displayed a superb UA oxidation process with a wide linear range (0.05–1000 µm) and a small LOD (10 nm).^[^ ^384^ ^]^ The high catalytic activity was attributed to the d‐band of Ru close to the Fermi level, which accelerated OH^−^ adsorption and increased the oxidation of small biomolecules.^[^ ^384^ ^]^ Zeng et al.^[^ ^397^ ^]^ fabricated an MMA‐based electrochemical non‐enzymatic glucose sensor. Pt SAs were deposited on Ni_6_Co_1_‐layered double hydroxides/N‐doped graphene (Pt_1_/Ni_6_Co_1_LDHs/NG), exhibiting a small oxidative potential (0.440 V) with improved sensitivity (273.78 µA mm ^−1^ cm^−2^) toward glucose. Furthermore, Pt_1_/Ni_6_Co_1_LDHs/NG displayed good selectivity and stability during 5‐week tests.^[^ ^397^ ^]^ The high binding energy between Pt_1_/Ni_6_Co_1_LDHs/NG and glucose and the collaborative effect of the Pt SAs, Co doping, and NG were the main reasons for the high glucose‐detection ability.^[^ ^397^ ^]^ Yang et al.^[^ ^55^ ^]^ developed a Fe/Cu‐NC material (DMAs as DAzyme) for high‐sensitivity and ‐selectivity S^2−^ colorimetric detection in water samples. The Fe/Cu‐NC material was able to detect S^2−^ over a wide concentration range of 0.09–6 µmol L^−1^ and an excellent detection limit of 30 nmol L^−1^.^[^ ^55^ ^]^ Chen et al.^[^ ^421^ ^]^ produced TMA sites for ascorbic‐acid sensing. They synthesized FeCoZn TMAs anchored on a S/N doped carbon matrix (FeCoZn‐TAC/SNC) via pyrolysis. The FeCoZn‐TAC/SNC material exhibited improved catalytic activity with respect to the SMA and DMA sites (FeZn‐, CoZn‐, and FeCo‐DAC/NC and Fe‐, Zn‐, Co‐SAC/NC). The high activity was attributed to the activation of O_2_ into ^•^O^2–^ radicals via TMA active sites, indicating a synergistic effect. A nanozyme sensor containing oxidase‐like FeCoZn‐TAC/SNC exhibited a wide linear range of 0.01–90 µm and a low detection limit of 6.24 nm determined by the colorimetric sensing of ascorbic acid. The FeCoZn‐TMA peroxidase‐like material was used as a colorimetric sensor to detect seven preservatives in food.^[^ ^422^ ^]^ During detection, the π–π stacking interaction and H bonding assisted in the absorption of food preservatives onto the surface of the nanozyme.^[^ ^422^ ^]^ The catalytic activity of FeCoZn TAzyme was modified, which differentiated colorimetric signal variations, yielding unique “fingerprints” corresponding to individual food preservatives.^[^ ^422^ ^]^

SMAs and DMAs are involved in gas sensing.^[^ ^19^, ^401^, ^407^, ^423^, ^424^, ^425^ ^]^ Aggarwal et al.^[^ ^423^ ^]^ reported Co‐based MOFs (metal–organic frameworks) for amines and on‐site ammonia detection. Based on the fluorescence behavior of MOF materials, they sensed both chemicals.^[^ ^423^ ^]^ The authors reported that the aliphatic amines and aromatic amines corresponded to the “turn‐off” and “turn‐on” fluorescence intensities of the two MOFs, resulting in the detection of amines.^[^ ^423^ ^]^ In contrast, the MOF‐based hybrid membranes exhibited a color change due to the release of ammonia during chemical reactions, leading to the naked eye sensing of ammonia.^[^ ^423^ ^]^

Li et al. ^[^ ^424^ ^]^ designed and developed Cu active sites on WO_2.72_ nanowires for sensing toluene at the ppb level (*R*_a_/*R*_g_  = 1.9 at 10 ppb) with high selectivity. The high selectivity for toluene gas detection was attributed to the strong binding sites presented by Cu SAs for toluene over other gaseous molecules.^[^ ^424^ ^]^ Jiang et al.^[^ ^425^ ^]^ used Co–Ni co‐doped in W_18_O_49_ (called Co‐Ni‐W_18_O_49_; DMAs) to sense triethylamine (TEA) gas species. The DFT results showed strong binding interactions and rapid charge transfer between the TEA gas and Co‐Ni‐W_18_O_49_ material.^[^ ^425^ ^]^ Owing to the synergistic modification of electronic configuration, the Co‐Ni‐W_18_O_49_ material exhibited excellent detection responses, rapid response/recovery, and high selectivity compared to Ni‐W_18_O_49_ and W_18_O_49_ at 250 °C, indicating its potential use in the manufacture of low‐cost gas sensors.^[^ ^425^ ^]^

SMAs and MMAs are also used in electrochemiluminescence (ECL) and photoelectrochemical sensors.^[^ ^19^, ^426^, ^427^, ^428^, ^429^ ^]^ ECL assays are applied for drug safety, clinical diagnostics, bioimaging, and atmospheric testing because of their acceptable sensitivity, easy handling, and minimal reagent use.^[^ ^426^ ^]^ In this regard, Fu et al.^[^ ^427^ ^]^ synthesized Ni SAs using a high‐temperature calcination strategy. The Ni SA was accelerated by dissolved oxygen, which delivered many reactive oxygen species (ROS) through the ORR on the cathode, improving the ECL signal significantly.^[^ ^427^ ^]^ The dispersibility of Ni SAs was improved by the functionalization of PEG 2000 (polyethylene glycol 2000) (called Ni@PEG).^[^ ^427^ ^]^ Ni@PEG was used as ECL probes to label the biorecognition species.^[^ ^427^ ^]^ Accordingly, Ni@PEG exhibited a wide linear range of 73–7.3 × 10^6^ CFU mL^−1^ and a lower LOD of 25 CFU mL. The recovery values for sample analysis were between 80.8 and 119.2%.^[^ ^427^ ^]^ The photoelectrochemical (PEC) immunoassay function was based on the photocurrent of photoactive materials, triggered by the antigen and antibody recognition.^[^ ^428^, ^429^ ^]^

In 2022, Kwok et al.^[^ ^428^ ^]^ used CuO nanoparticle‐assisted with Pt SAs embedded on Zn_0.5_Cd_0.5_S nanocrystals (denoted as CuO‐Pt‐Zn_0.5_Cd_0.5_S) to sense early‐stage prostate cancer (i.e., prostate‐specific antigen; PSA) via a photoelectrochemical immunoassay. The CuO‐Pt‐Zn_0.5_Cd_0.5_S‐based photoelectrochemical immunoassay delivered good PEC signals toward PSA (dynamic linear range 1.0 to 10 000 pg mL^−1^) and a low LOD (0.22 pg mL^−1^).^[^ ^428^ ^]^ The detection of CuO‐Pt‐Zn_0.5_Cd_0.5_S material in human serum specimens was similar to a commercial ELISA kit, which opens up the design of high‐performance photoelectrochemical sensors.^[^ ^428^ ^]^ Interestingly, the ion exchange reaction between Cu^2+^ ions and Pt‐Zn_0.5_Cd_0.5_S was responsible for the detection of PSA. More recently, Tang et al.^[^ ^429^ ^]^ introduced a multi‐metal atom (ZnCdFeMnCu)_x_S‐based photoelectrochemical immunoassay for sensing PSA. Under the optimized conditions, the (ZnCdFeMnCu)_x_S‐based PEC immunoassay displayed a better photocurrent response for target PSA determination with a broad linear range of 0.1–50 ng mL^−1^ and a lower LOD of 34.1 pg mL^−1^.^[^ ^429^ ^]^

Pt and Fe SAs (SMAs) have been used to improve the non‐enzymatic glucose and H_2_O_2_ detection.^[^ ^389^, ^390^, ^396^ ^]^ Zeng et al.^[^ ^389^ ^]^ synthesized Pt anchored on Ni(OH)_2_ nanoplates/N‐doped graphene (Pt_1_/Ni(OH)_2_/NG) hybrid as a non‐enzymatic glucose sensor. Pt_1_/Ni(OH)_2_/NG has a low anode peak potential (0.48 V) and an enhanced sensitivity (220.75 µA mm ^–1^ cm^–2^) toward glucose. In addition, the Pt SA‐based hybrid exhibited high selectivity, short response time, and good stability.^[^ ^389^ ^]^ They reported that Pt SAs sites helped improve the binding energy of glucose, and Ni provided rapid electron transfer, achieving good detection properties.^[^ ^389^ ^]^

**Figure 31:**
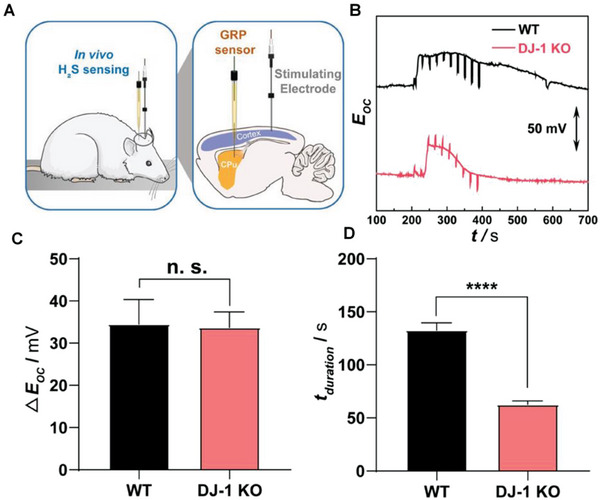
Detection of H_2_S in a mouse brain. a) Schematic diagram presenting the model of electrical stimulation of the cortex‐striatum and the implantation of the GRP sensor. b) Real‐time examination of the striatum H_2_S in the cortex of WT (black curve) and DJ‐1 KO (red curve) mice. c) Statistical findings of Δ*E*_OC_ in H_2_S detecting of WT (*n*  = 4) and DJ‐1 KO mice (*n*  = 4). d) Statistical information of *t*_duration_ in H_2_S detecting of WT (*n*  = 4) and DJ‐1 KO mice (*n*  = 4). Reproduced with permission.^[^ ^393^ ^]^ Copyright 2022 American Chemical Society.

Shan et al.^[^ ^396^ ^]^ fabricated Pt SA‐doped Cu@CuO core–shell nanowires (Pt_1_/Cu@CuO NWs) for nonenzymatic glucose sensors. Pt_1_/Cu@CuO NWs displayed a high sensitivity of 852.163 µA mm ^−1^ cm^−2^, low LOD of 3.6 µm, dynamic linear range of 0.01–5.18 µm, excellent selectivity, and high durability.^[^ ^396^ ^]^ The good detection performance was attributed to the collective effect of Pt SAs and Cu@CuO NWs, which provided the binding strength of glucose at the NW surface.^[^ ^396^ ^]^ Mao et al.^[^ ^393^ ^]^ reported the atomically dispersed NiN_4_ active sites for the detection of hydrogen sulfide (H_2_S) in living mice (**Figure** **31**). The NiN_4_‐SMAs‐GRP microsensors were inserted in the striatum area to detect the H_2_S concentration (Figure 31A).^[^ ^393^ ^]^ The OCV (E_OC_) profiles indicated that cortical stimulation triggered increased interstitial H_2_S quantities in the striatum (Figure 31B).^[^ ^393^ ^]^ The maximum increases in E_OC_ (ΔE_OC_) during all stimulating pulses showed no significant difference between WT mice (34.5 ± 10.1 mV, n = 4) and DJ‐1 KO mice (33.7 ± 6.4 mV, n = 4) (Figure 31C).^[^ ^393^ ^]^ In addition, the *t*_duration_ (i.e., maintaining time that the E_OC_ decreases to 80%) of DJ‐1KO was 62.6 ± 5.8 s (n = 4), which was much smaller than the WT group (132.7 ± 11.8 s, n = 4) (Figure 31D).^[^ ^393^ ^]^ These results highlight the potential of SMA‐based detection platforms for the real‐time monitoring of neurochemical processes.

SMAs/DMAs have been reported for sensing applications. On the other hand, no reports based on TMAs/MMAs were found. Therefore, researchers need to work continuously on DMAs/TMAs/MMA‐based materials for sensors to understand the fundamental physical and chemical properties of materials. DMAs/TMAs/MMAs have excellent detection properties different from SMAs owing to their additional modification in the center of the metal d‐band.

### 6.11 Biomedical

For biomedicine applications, the catalytic performance is an important parameter that needs to be optimized. SAzymes (SMA with enzyme‐like properties) with superb catalytic activities have attracted considerable attention over the past few years owing to the crucial role they can play in improving community health.^[^ ^430^, ^431^, ^432^, ^433^, ^434^ ^]^ **Table** **14** lists the kinetic parameters and respective enzyme mimic properties of various SAzymes and DAzymes. Various metal atoms have diagnostic and therapeutic potential owing to their outstanding properties compared to their nanoparticle counterparts.^[^ ^430^, ^431^, ^432^, ^433^, ^434^ ^]^ Well‐controlled molecular structures are bridging the gap between natural and biological enzymes and have been given more importance in biology to mimic the enzyme functionality from antimicrobial activity to therapy. This section provides a detailed review of the recent progress of SAzymes, DAzymes, TAzymes, and MAzymes in biomedical applications, including anti‐bacterial/viral/tumor/inflamatory.

**Table 14:** Kinetic parameters of different SAzymes and DAzymes with the TMB substrate.

Metal atoms	Support	Enzymatic mimics	*K*_m_ [mM]	V_max_	Catalytic efficiency [*K*_cat._/*K*_m_]	Categories	Reference
Cu	Carbon nanosheets	POD^a)^ mimic	19.94	20.07×10^−8^ (m s^−1^)	3.80 (m ^−1^s^−1^)	SAzymes	[435]
Cu	–	POD^a)^ and OXD^b)^ mimic	–	–	–	SAzymes	[433]
Cu	*g*‐C_3_N_4_	POD^a)^ mimic	0.01	–	1.6 ×10^4^ (mm ^−1^ s^−1^)	SAzymes	[436]
Cu	Ti_3_(A_lx_Cu_1‐x_)C_2_	POD^a)^ mimic	2.35	0.58 (m s^−1^)	–	SAzymes	[437]
Zn	Carbon nanomaterial	POD^a)^ mimic	0.22	10.66×10^−8^ (m s^−1^)	–	SAzymes	[438]
Fe	N‐doped/C	POD^a)^ mimic	3.92	5.88×10^−7^ (m s^−1^)	–	SAzymes	[439]
Fe	Carbon nanotubes	POD^a)^ mimic	0.12	1.56 × 10^−7^ (m s^−1^)	–	SAzymes	[440]
Fe	Porous carbon	POD^a)^ mimic	–	–	–	SAzymes	[441]
Fe	N‐doped C	POD^a)^ mimic	5.20	14.90×10^−7^ (m s^−1^)	–	SAzymes	[442]
Co	N‐doped C	POD^a)^ mimic	5.06	1.90×10^−7^ (m s^−1^)	–	SAzymes	[442]
Zn	N‐doped C	POD^a)^ mimic	0.28	0.43×10^−7^ (m s^−1^)	–	SAzymes	[442]
Fe	Carbon nanoframes	POD^a)^ mimic	–	–	–	SAzymes	[443]
Fe	N‐riched carbon	POD^a)^ mimic	0.65	8.47 (µm s^−1^)	6.01 (mm ^−1^ s^−1^)	SAzymes	[444]
Fe	‐	POD^a)^ mimic	2.06×10^−3^	4.65×10^−5^ (m min^−1^)	1.40 × 10^8^ (m ^−1^ min^−1^)	SAzymes	[445]
Pt	CeO_2_	POD^a)^, CAT^d)^, SOD^c)^, GP_x_ ^e)^	–	–	–	SAzymes	[446]
Pt_TS_ (thermally stable)	‐	POD^a)^ mimic	0.31	2.83 × 10^−7^ (m s^−1^)	1.06 × 10^6^ (m ^−1^ s^−1^)	SAzymes	[447]
Cu	Carbon dots	POD^a)^ mimic	1.37	6.57×10^−7^ (m s^−1^)	–	SAzymes	[448]
Co	N‐doped porous carbon	POD^a)^ mimic	0.51	1.34 ×10^−7^ (m s^−1^)	–	SAzymes	[449]
Fe	N‐doped porous carbon	POD^a)^ mimic	0.09	7.36 ×10^−8^ (m s^−1^)	0.527 (mm ^−1^ s^−1^)	SAzymes	[450]
Fe	N‐doped porous carbon nanoparticles‐edge	POD^a)^ mimic	0.57	1.24 ×10^−6^ (m s^−1^)	1.52 (mm ^−1^ s^−1^)	SAzymes	[450]
Fe	P‐carbon nanowires	POD^a)^ mimic	0.60	79.84 (µm min^−1^)	–	SAzymes	[451]
Fe	Carbon nanowires	POD^a)^ mimic	0.96	132.58 (µm min^−1^)	–	SAzymes	[451]
Fe	N‐doped porous carbon nanoparticles‐edge	OXD^b)^ mimic	0.37	4.2 ×10^−8^ (m s^−1^)	0.078 (mm ^−1^ s^−1^)	SAzymes	[450]
Co	N_2_‐C	OXD^b)^ mimic	0.154	65.2 ×10^−8^ (m s^−1^)	–	SAzymes	[452]
Co	N_3_‐C	OXD^b)^ mimic	0.073	114.5×10^−8^ (m s^−1^)	–	SAzymes	[452]
Co	N_4_‐C	OXD^b)^ mimic	0.100	95.5 ×10^−8^ (m s^−1^)	–	SAzymes	[452]
Fe	N‐doped C	OXD^b)^ mimic	0.13	2.25×10^−8^ (m s^−1^)	–	SAzymes	[439]
Fe	N‐doped C	Fenton	–	–	–	SAzymes	[453]
Fe	Carbon nanoframes	OXD^b)^ like	0.148	0.758 (µm s^−1^)	4.79 (mm ^−1^ s^−1^)	SAzymes	[454]
Mn	Carbon nanoframes	OXD^b)^ like	0.253	0.400 (µm s^−1^)	1.48 (mm ^−1^ s^−1^)	SAzymes	[454]
Co	Carbon nanoframes	OXD^b)^ like	0.682	0.177 (µm s^−1^)	0.26 (mm ^−1^ s^−1^)	SAzymes	[454]
Ni	Carbon nanoframes	OXD^b)^ like	0.120	6×10^−4^ (µm s^−1^)	5×10^−3^ (mm ^−1^ s^−1^)	SAzymes	[454]
Cu	Carbon nanoframes	OXD^b)^ like	0.124	4.7×10^−4^ (µm s^−1^)	4 ×10^−3^ (mm ^−1^ s^−1^)	SAzymes	[454]
Fe	N‐doped C	OXD^b)^ mimic	0.62	5.26×10^−7^ (m s^−1^)	–	SAzymes	[455]
Ni	N‐doped C	OXD^b)^ mimic	0.284	2.45×10^−8^ (m s^−1^)	–	SAzymes	[455]
Fe	N‐doped porous carbon	SOD^c)^ and CAT^d)^ mimic	‐	–	–	SAzymes	[456]
Zn/Mo	Amphiphilic aerogel	POD^a)^ mimic	0.43	3.84×10^−8^ (m s^−1^)	188.80 (Zn) 58.79 (Mo) (m ^−1^ s^−1^)	DAzymes	[457]

^a)^ POD, peroxidase;

^b)^ OXD, oxidase;

^c)^ SOD, superoxide dismutase;

^d)^ CAT, catalase;

^e)^ GP_x_, glutathione peroxidase.

SAzymes are the most studied catalysts because they have superior catalytic activity to conventional nanozymes by 10 to 100 times. Typically employed methods for synthesizing SAzymes frequently suffer from inadequate interactions between the metal active atoms and supports, leading to instability and the leaching of the active species. The presence of individual metal centers, separated at the atomic level, enhances the efficiency and density of active sites.

Ji et al.^[^ ^445^ ^]^ engineered the FeN_3_P SAzyme, which exhibited peroxidase‐like activity consistent with the Michaelis–Menten profile. Owing to the similarity in the chemical environment of as‐synthesized FeN_3_P and the active site of the horseradish peroxidase (HRP), the SAzyme catalyzed the peroxidation of 3,3′,5,5′‐tetramethylbenzidine (TMB), diazoaminobenzene (DAB), and o‐phenylenediamine (OPD), which can be monitored through a colorimetric response. The FeN_3_P SAzyme exhibits exceptional catalytic efficiency across a broad temperature range (30–60 °C) and various pH values (3–5). On the other hand, for comparative analysis between the FeN_3_P SAzyme and the natural enzyme HRP, the evaluation was conducted under optimal conditions that were more suitable for the natural enzyme, namely, a temperature of 37 °C and a pH of 3.6. The measured specific activity of FeN_3_P‐SAzyme was remarkably higher, reaching 316 U mg^−1^. In contrast, the Fe_3_O_4_ nanozyme displayed a specific activity of 9.12 U mg^−1^, which was more than 30 times less active. Similarly, the FeN_4_‐SAzyme exhibited a specific activity of 33.8 U mg^−1^, which was ≈10 times lower than that of the FeN_3_P‐SAzyme. In kinetic analysis, the *K*_m_ value is commonly used to assess the binding affinity, with a lower *K*_m_ indicating a stronger interaction between the nanozyme and substrates. The FeN_3_P‐SAzyme demonstrated a comparable catalytic efficiency (*k*_cat_/*K*_m_ = 1.40 × 10^8^ m ^−1^ min^−1^) and substrate affinity (*K*_m_ = 2.06 × 10^−3^ mm) to the natural HRP enzyme (*k*_cat_/*K*_m_ = 1.15 ×10^7^ m ^−1^ min^−1^, *K*_m_ = 5.55 mm), during oxidation of the peroxidase substrate TMB. Leveraging the elevated peroxidase‐like activity of the FeN_3_P‐SAzyme, which enabled the selective generation of abundant oxidative species within a tumor‐acidic environment, the authors used FeN_3_P‐SAzyme as an efficient therapeutic approach for suppressing tumor cells. A concentration‐dependent response was observed when human HepG2 hepatoma cells were treated with FeN_3_P‐SAzyme, demonstrating the potential effectiveness of FeN_3_P‐SAzyme in tumor cell suppression. The same outcomes were observed in HT‐29 colon cancer cells, SKOV‐3 ovarian cancer cells, 4T1 murine breast cancer cells, and HeLa cervical cancer cells. Hence, FeN_3_P‐SAzyme possesses a versatile inhibitory potential against various tumor cell types, displaying its universal effectiveness.

Chen et al.^[^ ^447^ ^]^ reported high‐performance nanozymes via direct atomization of Pt NPs into Pt_TS_‐SA, exposing the metal catalytic sites and dramatically enhancing enzymatic performance. Following the thermal atomization process, where platinum nanoparticles (Pt NPs) were transformed into individual atoms, the peroxidase‐like catalytic activity of the Pt active sites experienced a substantial enhancement, increasing from 1.05 units to 21.8 units (U). One activity unit is defined as the quantity of nanozyme that catalyzes 1 µmol of product per minute per nmol of Pt atoms. The thermally atomized Pt_TS_‐SAzyme displays the Michaelis–Menten kinetic profile during the oxidation of peroxidase substrate TMB, showing a significantly higher catalytic rate constant (*k*_cat_ = 329 s^−1^) and catalytic efficiency (*k*_cat_/*K*_m_ = 1.06 × 10^6^ m ^−1^ s^−1^) compared to the Pt‐NPs nanozyme (*k*_cat_ = 8.67 s^−1^; *k*_cat_/*K*_m_ = 9.96 × 10^3^ m ^−1^ s^−1^). Pt_TS_‐SAzyme displays remarkable peroxidase‐like activity of 21.8 U nmol^−1^ Pt atom, which was significantly higher than that of Pt‐SAzyme (4.19 U/nmol Pt atom). Moreover, the *k*_cat_/*K*_m_ value for Pt_TS_‐SAzyme is 1.06 × 10^6^ m ^−1^ s^−1^, which was 5.9 times greater than that of Pt‐SAzyme (1.79 × 10^5^ m ^−1^ s^−1^), indicating Pt_TS_‐SAzyme has superior catalytic kinetics. Furthermore, the antibacterial effects of Pt_TS_‐SAzyme and Pt‐NPs nanozyme were evaluated against five bacteria Gram‐negative bacteria (*Escherichia coli* (*E. coli*), *Pseudomonas aeruginosa* (*P. aeruginosa*), *Salmonella enteritidis* (*S. enteritidis*), *Klebsiella pneumoniae* (*K. pneumoniae*)), and Gram‐positive *Staphylococcus aureus* (*S. aureus*). Peroxidase‐like nanozymes have been studied extensively for their potential antibacterial applications. Their peroxidase‐like catalytic activity has been used to break down H_2_O_2_ into highly toxic free radicals, disrupting the bacterial membrane and triggering bacterial death. In 1 mm H_2_O_2_, at a 0.25 mg mL^−1^ concentration, Pt_TS_‐SAzyme had potent antibacterial effects against the five bacterial strains tested. Remarkably, it achieved an inhibition rate of ≥90% against *E. coli*, *P. aeruginosa*, *S. enteritidis*, and *K. pneumoniae* while displaying an inhibition rate of 81% against *S. aureus*.

Xu et al.^[^ ^438^ ^]^ synthesized a Zn‐based zeolitic imidazolate framework‐derived carbon nanomaterial with atomically dispersed Zn atoms with a peroxidase mimic (PMCS). This SAzyme had a potent in vitro antibacterial effect, inhibiting the growth of *Pseudomonas aeruginosa* by up to 99.87% while also notably enhancing wound healing. The exceptional peroxidase‐like activity of PMCS can be attributed to the presence of coordinatively unsaturated Zn–N_4_ sites, enabling the decomposition of H_2_O_2_ and facilitating the generation of hydroxyl radicals (•OH). The plate count method was used to assess the in vitro antibacterial properties of PMCS against *Pseudomonas aeruginosa*. In the presence of H_2_O_2_, PMCS showed remarkably potent antibacterial effects, with growth inhibition of *P. aeruginosa* reaching up to 99.87%. Its therapeutic potential was evaluated by establishing a wound infection model in mice using *P. aeruginosa* to infect wounds. Treatment with PMCS and a low concentration of H_2_O_2_ (100 µm) resulted in effective wound healing.

A highly efficient CNT/FeNC nanozyme containing robust atomic Fe–Nx moieties was synthesized, exhibiting exceptional peroxidase‐like activity. This CNT/FeNC nanozyme was used as the signal element in a series of paper‐based bioassays, enabling the ultrasensitive detection of H_2_O_2_, glucose, and ascorbic acid.^[^ ^440^ ^]^ The calculated turnover number (TON) of the single‐atom‐dispersed CNT/FeNC nanozyme was 0.23 × 10^−4^/1.7 × 10^−7^ = 135 cycles in 5 min. The measured *K*_m_ of the single‐atom dispersed CNT/FeNC nanozyme was significantly lower than that of the conventional Fe_3_O_4_ nanozyme.

Zhang et al.^[^ ^449^ ^]^ designed hollow mesoporous Co single‐atom nanozyme (SAN) to mimic biological processes. The primary objective was to target inflamed regions, quell the presence of inflammatory agents, and effectively eliminate deeply entrenched bacteria to enhance the eradication of biofilms. After accumulation at the sites of infection facilitated by specific receptors on the RAW 264.7 cell membrane (RCM), Co@SAHS catalyzes the conversion of H_2_O_2_ into hydroxyl radicals. This catalytic process was augmented by the near‐infrared II (NIR‐II) photothermal effect and a decrease in glutathione levels, enabling permeation and disintegration of the biofilm structure. This interleukin can reprogram macrophages, effectively curbing the oxidative damage and mitigating tissue inflammation.

Oxidase‐like activity was also observed for the SAzyme featuring active centers of MN_5_ confined within a carbon nanoframe.^[^ ^454^ ^]^ FeN_4_ SA/CNF and MN_5_ SA/CNF, where (M is Mn, Fe, Co, Ni, or Cu), demonstrated oxidase‐like activity in the following order: FeN_5_ SA/CNF > MnN_5_ SA/CNF > CoN_5_ SA/CNF > FeN_4_ SA/CNF ≫ NiN_5_ SA/CNF > CuN_5_ SA/CNF. Hence, the central metal atom and the five‐N‐coordinated structure equally influence the performances of single‐atom nanozymes.

Maintaining a proper balance of intracellular reactive oxygen species (ROS) is crucial, as low levels of ROS function as secondary messengers in cell signaling, aiding in cell proliferation, metabolic regulation, apoptosis, and pathogen resistance.^[^ ^458^, ^459^ ^]^ Conversely, high ROS levels exacerbate tissue damage, causing chronic inflammatory conditions such as wound sepsis, inflammatory bowel disease, and acute liver injury.^[^ ^460^, ^461^ ^]^ Therefore, restoring intracellular redox balance is essential. Notably, SAs mimic natural enzymes such as SOD and catalase, with ROS‐scavenging functions that protect cells from oxidative stress. Inflammatory responses often involve the release of pro‐inflammatory cytokines such as tumor necrosis factor α (TNF‐α), interleukin 6 (IL‐6), and IL‐1β. SAzymes can influence the signaling pathways regulating cytokine production, thereby reducing inflammation. Additionally, SAzymes can interact directly with immune cells such as macrophages and neutrophils, modulating the activation and polarization of these cells to promote an anti‐inflammatory phenotype in macrophages, resolving inflammation.

In this regard, Yang et al.^[^ ^459^ ^]^ synthesized Cu‐SAzyme for treating sepsis by mimicking SOD activity to eliminate O_2_^•−^. A lipopolysaccharide (LPS)‐induced inflammation model was used to simulate the cell microenvironment associated with sepsis. The Cu‐SAzyme treatment resulted in a fourfold reduction of O_2_^•−^, comparable to the effects of SOD in the LPS‐induced inflammation model. Additionally, the levels of proinflammatory cytokines, TNF‐α and IL‐6, secreted by the cells in the inflammatory model were significantly reduced in the presence of Cu‐SAzyme. Cao et al.^[^ ^461^ ^]^ demonstrated that Fe SAzyme‐modified Bifidobacterium longum probiotics can restore a healthy immune system when used to manage inflammatory bowel disease. Boronic acid–poly(ethylene glycol) was used as a linker to functionalize Fe SAs, enabling the SAs to bind to probiotics via a click reaction. Consequently, the modified SAzyme effectively reduced ROS and inflammation, restoring the integrity of the damaged intestinal barrier.

Yan et al.^[^ ^462^ ^]^ prepared Pt‐SA‐based nanozyme bandages for preventing neuroinflammation and noninvasively treating brain trauma. Pt SA/CeO_2_ scavenged O_2_^•–^ and ^•^NO, yielding multi‐antioxidant activities. Furthermore, Pt SA/CeO_2_ effectively mitigated LPS‐triggered injuries and H_2_O_2_‐induced cellular damage, while exerting an anti‐inflammatory effect by decreasing the levels of TNF‐α, IL‐1β, and IL‐6. Cao et al.^[^ ^460^ ^]^ synthesized N‐doped carbon‐supported atomically dispersed Co‐porphyrin centers (Co/PMCS) as a multi‐antioxidant SAzyme for sepsis management. The Co/PMCS can sequentially eliminate H_2_O_2_ and O_2_^•–^ by mimicking the actions of multiple enzymes, including SOD, catalase, and glutathione peroxidase. In addition, this material can efficiently reduce ^•^OH through an oxidation‐reduction cycle, exhibiting unparalleled activities that surpass conventional nanozymes. This represents the first instance of such a multi‐antioxidant SAzyme design. When applied for managing LPS‐induced sepsis and bacteremia, Co/PMCS effectively mitigated the elevated levels of reactive O and N species, consequently suppressing the production of proinflammatory cytokines. Therefore, infected mice exhibited a significant survival advantage.

DMAs further enhanced the activities of nanozymes owing to the presence of electron‐rich (nucleophiles) and electron‐poor (electrophiles) species, which can regulate the adsorption and desorption properties of reaction intermediates. In this regard, Ma et al.^[^ ^457^ ^]^ reported a simple method for synthesizing DAzymes containing Zn and Mo. The Zn/Mo DSAC‐SMA exhibited elevated *k*_cat_/*K*_m_ coefficients relative to those observed in nanozymes relying on either Zn or Mo constituents. This outcome further substantiates the collaborative influence of Zn and Mo in augmenting catalytic efficacy. This novel approach enables the detection of a diverse array of substances, encompassing intracellular H_2_O_2_, glucose in serum, cholesterol, and ascorbic acid in commercial beverages.

**Figure 32:**
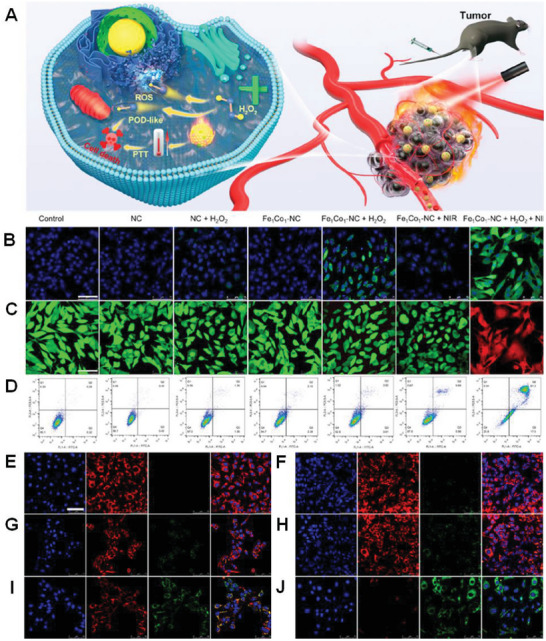
A) Schematic diagram presenting the fabrication method and photo‐activated tumor catalytic therapy of nanozymes. B) DCF fluorescence was measured in mouse melanoma cells treated with various samples. C) Live/dead assays. D) Flow cytometry results measured in the control, NC, NC + H_2_O_2_, Fe_1_Co_1_‐NC, Fe_1_Co_1_‐NC + H_2_O_2_, Fe_1_Co_1_‐NC + NIR, and Fe_1_Co_1_‐NC + H_2_O_2_ + NIR. E–J) JC‐1 fluorescence intensity of mitochondrial membrane potential measured in the control, NC, NC + H_2_O_2_, Fe_1_Co_1_‐NC, Fe_1_Co_1_‐NC + H_2_O_2_, and Fe_1_Co_1_‐NC + H_2_O_2_ + NIR. The scale bar in (B–J) is 75 µm. Reproduced with permission.^[^ ^463^ ^]^ Copyright 2023 American Chemical Society.

Song et al.^[^ ^463^ ^]^ reported a series of dual‐atom (M_1_/M_2_‐NC; 13 types) metal‐N‐C nanozymes (M = Fe, Co, Ni, Mn, Ru, and Cu) that exhibited efficient peroxidase (POD)‐like activities. Among the various types of DMAs, Fe_1_Co_1_‐NC DAzymes with the Fe_1_‐N_4_/Co_1_‐N_4_ structure exhibited the maximum POD‐like activity. Therefore, they used DAzymes to remove tumor growth both in vitro and in vivo (**Figure** **32**), suggesting that DAzymes can be used in tumor treatments. As shown in Figure 32B, minimal green fluorescence was detected in the control, NC, Fe_1_Co_1_‐NC, and NC + H_2_O_2_ cohorts. In contrast, a moderate level of green fluorescence was observed in the Fe_1_Co_1_‐NC + H_2_O_2_ group, suggesting that Fe_1_Co_1_‐NC can facilitate the generation of ^•^OH from intracellular H_2_O_2_, exhibiting satisfactory peroxidase‐like (POD‐like) behavior. Intracellular reactive oxygen species (ROS) experiments suggested that the POD‐like performance of Fe_1_Co_1_‐NC could be augmented synergistically by the photothermal effect, elevating the intracellular ^•^OH concentration. The in vitro experimental findings confirmed that the Fe_1_Co_1_‐NCs can execute POD‐like catalytic therapy for inducing tumor cell apoptosis and amplify this effect through near‐infrared (NIR) photothermal mechanisms, ultimately achieving a synergistic and efficacious ablation of cancer cells. They also mentioned that the d‐band center position of the Fe atom site is modified by the Co atom site, resulting in excellent POD‐like activity.

Ai et al.^[^ ^20^ ^]^ engineered a PtPdRuRhIr ultra‐small high‐entropy alloy nanoparticle (US‐HEANPs) that serves as versatile nanoplatforms, exhibiting remarkable efficacy in tumor treatment. The synthesized US‐HEANPs highlight the exceptional peroxidase‐like (POD‐like) functionality, enabling them to facilitate the conversion of endogenous hydrogen peroxide into highly potent hydroxyl radicals. Moreover, these US‐HEANPs exhibit a pronounced ability to convert 808 nm near‐infrared light efficiently into thermal energy. In vivo and in vitro investigations showed that the US‐HEANPs effectively eradicated breast cancer cells through a synergistic interplay of their POD‐like activity and photothermal capability, offering a promising approach for tumor therapy.

Fan et al.^[^ ^464^ ^]^ synthesized Au@CuBCats, featuring a dual‐enzyme mimetic system comprised of GOx and POD functionalities. The primary objective was to manage glucose levels and bacterial populations within diabetic ulcers concurrently. Specifically, gold nanoparticles (AuNPs) within the catalyst exhibit GOx‐like properties, initiating glucose oxidation and generating H_2_O_2_. Subsequently, the generated H_2_O_2_ undergoes catalytic conversion into ^•^OH via the single copper atoms that mimic the POD activity. In vitro antibacterial assays showed that the bionanocatalysts promoted a substantial decrease in the bacterial populations, particularly targeting the multi‐drug‐resistant strains MRSA and ESLP *E. coli*. In vivo studies using a rabbit ear model with bacterial diabetic ulcers exhibited a fast and complete recovery of the ulcer, achieving a 100% healing rate without any residual signs of inflammation.

Xing et al.^[^ ^465^ ^]^ developed N‐doped mesoporous carbon nanomotors incorporating single‐atom Cu (Cu‐JMCNs). This innovative structure represents the fusion of single‐atom nanocatalytic therapeutic agents with the inherent propulsion capacity of nanomotors, all tailored for cancer treatment applications. The Cu atom serves as a catalyst, enabling the conversion of H_2_O_2_ into cytotoxic ^•^OH to facilitate the chemodynamic therapy (CDT). The orchestrated interplay between Cu‐JMCNs and near‐infrared (NIR) light prompts a self‐thermophoretic movement of the nanomotors. This motion was attributed to the distinctive asymmetric jellyfish‐like configuration and the photothermal attributes of the carbon material. Importantly, this propulsion mechanism significantly enhanced the internalization of the nanomotors into cells and their penetration through complex three‐dimensional tumor structures. In 2D cancer cells, the propulsion minimally affects CDT because of the excess catalytic activity compared to the limited intracellular H_2_O_2_ concentration. On the other hand, in 3D tumor models and in vivo, NIR propulsion improves nanomotor penetration, significantly boosting single‐atom CDT. The combined single‐atom CDT and NIR propulsion achieves more than 85% tumor inhibition, obviating the need for additional photothermal therapy.

Xing et al.^[^ ^466^ ^]^ successfully developed single‐atom gadolinium (Gd) supported on hollow N‐doped carbon nanospheres, enhancing the precision of MR imaging for tumor detection. In addition, the remarkable stability of the isolated Gd atoms, combined with the surface coating of polyethylene glycol (PEG), imparted excellent biocompatibility upon the Gd‐SA agent. Comparatively, in T_1_‐weighted MR imaging for tumor visualization within living subjects, the Gd‐SA agent exhibited superior spatial resolution when contrasted with Gd‐DTPA. In particular, even at a relatively low Gd concentration (120 µg kg^−1^ Gd), Gd‐SA distinctly delineated the boundary between tumor and normal tissue, showing a discernible demarcation in vivo.

Liu et al.^[^ ^467^ ^]^ reported heightened immunogenicity in tumor cells by instigating cascade immunogenic tumor ferroptosis. Lipoxygenase (LOX) and phospholipase A_2_ (PLA_2_) are co‐loaded onto a FeCo/Fe–Co dual‐metal atom nanozyme scaffold. This nanoplatform serves a dual purpose: it starts the initial stages of immunogenic tumor ferroptosis via its inherent multi‐enzyme mimetic functionalities and elevates arachidonic acid (AA) expression. This augmentation of AA expression cooperates with interferon‐gamma (IFN‐γ) derived from CD8^+^ T cells, inducing ACSL_4_‐mediated immunogenic tumor ferroptosis.

Mu et al.^[^ ^468^ ^]^ synthesized PtPdMo tri‐metallic nanozyme with a distinct preference for a neutral environment and assessed its efficacy in both cellular and animal modeling levels of brain injury. The tri‐metallic nanozymes exhibited notable catalytic proficiency attributed to structural lattice perturbations, facilitating the exposure of highly active sites. These nanozymes displayed multi‐faceted antioxidative properties, effectively scavenging reactive oxygen and nitrogen species (RONS) through multi‐enzyme‐mimetic reactions. In vitro investigations revealed the capacity of the tri‐metallic nanozyme to enhance the viability of neural cells under injury conditions. In the model of brain injury induced by lipopolysaccharide (LPS), applying the tri‐metallic nanozyme results in substantial restoration of SOD activity and mitigating lipid peroxidation. Furthermore, treatment with the tri‐metallic nanozyme significantly enhanced the survival rate, ameliorating neuroinflammation, and restoring the reference memory in the context of injured mice.

Despite the great progress and development of SAzymes and DAzymes, a critical contemporary challenge lies in succeeding enzyme‐like activity at a level that can genuinely substitute natural enzymes. SAzymes and DAzymes with low densities of single/double metal active sites consistently exhibited lower catalytic activity than natural enzymes. Furthermore, complexity still exists, which is a formidable obstacle to identifying the active sites and exploring the origins of enzyme‐like activity. Therefore, the high‐density active sites of SAzymes and DAzymes were synthesized, and TAzymes/MAzymes were constructed with densely exposed and dispersed triple/multiple atoms coordinated with C, N, S, P, B, and O species as a catalytic active site to enhance the enzyme activity. After addressing the above issue, efficient active centers based on SAzymes, Dazymes, Tazymes, and Mazymes can be developed for enzyme‐like activity.

## 7 Conclusion and Perspectives

**Figure 33:**
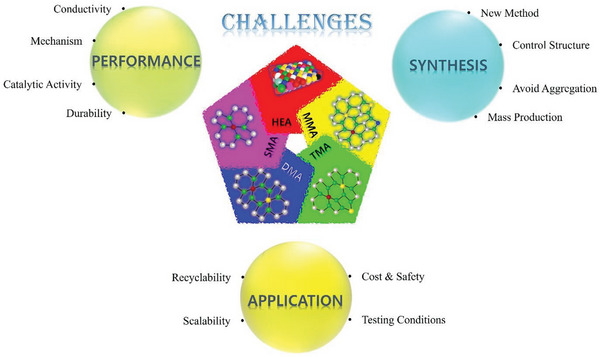
Challenges of SMAs/DMAs/TMAs/MMAs based materials for energy, sensors, and biomedical applications.

This paper systematically reviewed the progress of the last few years in advanced fabrication and numerous applications of SMA/DMA/TMA/MMA‐based materials. The synthetic approaches and benefits of DMA/TMA/MMA catalysts for developing high‐performance catalysts for energy, sensors, and medical applications are enlightened. Owing to the large surface area, highly modulable Fermi level, specific sites for selective small molecule sensing, nanosheets with tunable‐porosity, and rich active sites, SMA/DMA/TMA/MMA materials have promising applications in electrolysis of water, fuel cells, ZABs, sensors, and biomedical processes. Although enormous successes have been achieved in developing SMAs/DMAs/TMAs/MMAs over the last couple of years, more effort is needed to address many existing challenges associated with fabrication, activity, and mass production. This section discusses existing issues and challenges in developing SMAs/DMAs/TMAs/MMAs materials from the perspectives of synthesis, performance, and industrial applications (**Figure** **33**).

### 7.1 Controlled Synthesis

**Figure 34:**
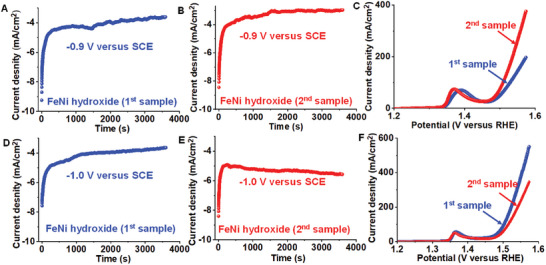
A,B) Deposition of FeNi hydroxide at −0.9 V vs. SCE and C) their corresponding OER activity in an oxygen‐saturated 1 m KOH electrolyte. C,D) Deposition of FeNi hydroxide at −1.0 V vs. SCE and C) their corresponding OER activity in an oxygen‐saturated 1 m KOH electrolyte. The catalytic activities (C,F) suggested that the performance of OERs is not reproducible, although the material was synthesized with similar potential.

**Figure 35:**
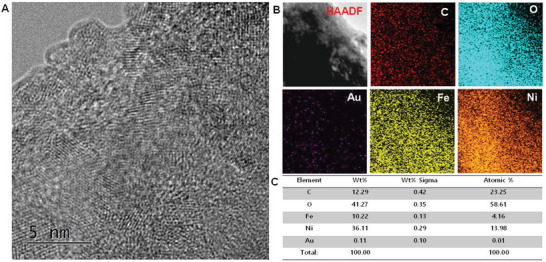
A) HRTEM image of Au doped FeNi oxyhydroxide. B) Elemental mapping images of C, O, Au, Fe, and Ni for the composition of Au‐doped FeNi oxyhydroxide material. C) Elemental composition for Au‐doped FeNi oxyhydroxide, indicating the presence of Au SAs.

**Figure 36:**
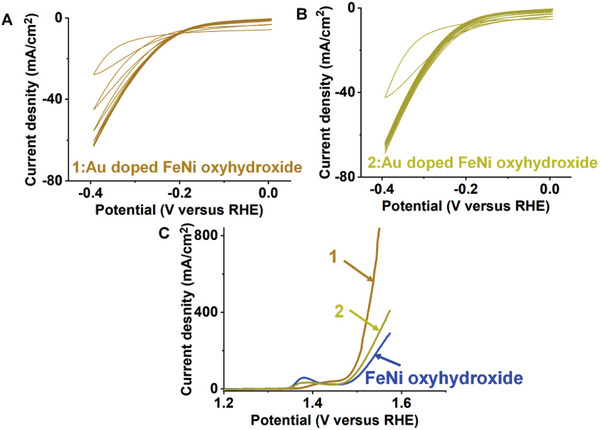
A,B) Cyclic voltammograms (CVs) of electrochemical deposition of Au single atoms (AuSAs) on FeNi oxyhydroxide, which is recorded by scanning the potential from −1.06 to −1.46 V vs SCE in KOH (1 m)‐Au precursor at and 50 mV S^−1^. Ten consecutive CV cycles were used for AuSAs deposition. C) OER activity of different electrodes (1: Au doped FeNi oxyhydroxide; 2: Au doped FeNi oxyhydroxide; 3: FeNi oxyhydroxide) in an oxygen‐saturated 1 m KOH solution.

As mentioned in the earlier sections, the performance of catalysts depends mainly on the kinetics of catalytic reactions. The kinetics is related directly to materials design and synthesis. Therefore, new catalysts are needed to reduce the reaction energy barriers and improve the kinetics. In this regard, SMAs/DMAs/TMAs/MMAs have various advantages from their unusual structures. These catalysts are synthesized mainly using defect/vacancy‐mediated, ligand‐mediated, and pyrolysis approaches. In the case of the defect‐rich method, a profuse of topological vacancies (defects) first generated in the 1D/2D materials (carbon or non‐carbon supports). These lattice/carbon defects are filled with SMAs/DMAs/TMAs/MMAs. Therefore, controlling the multi‐heteroatoms substituted 1D/2D materials in the first (defect generation) step synthesis is difficult because the above method involves two steps. In addition, the different types of metal ions compete with each other (owing to their surface energy differences) while trapping SMAs/DMAs/TMAs/MMAs in/on defective 1D/2D materials, resulting in metal ion aggregation to form nanoparticles (NPs). In the case of the ligand‐mediated approach, the coordination of the metal cations by ligands reduces metal agglomeration at the lowest temperature synthesis, leading to the formation of SMA/DMA/TMA/MMA materials. Although the ligand‐assisted self‐assembly approach allows the synthesis of SMAs/DMAs/TMAs/MMAs, this strategy only works at low temperatures (below 600 °C), making the development of highly active sites for chemical reactions difficult. The most common approach toward SMAs/DMAs/TMAs/MMAs involves the pyrolysis of synthesized hybrids/frameworks/composites. The main problem with this process is the aggregation of metal ions to form the NPs during the pyrolysis at a higher temperature (above 800 °C). Although the above methods have been reported for the fabrication of SMA/DMA/TMA/MMA materials with controlled configuration and large‐scale production, they are suffering from an incomplete understanding of the dynamic arrangements of metal atoms and ligands during fabrication. Therefore, more effort is needed to unveil this dynamically reacting black box. In addition, a new method is needed to predict/control the coordination numbers during the synthesis process. The electrochemical deposition process is also a simple, fast, and powerful tool for depositing SMAs in the defect regions of 2D materials. Single Au atoms embedded on FeNi oxyhydroxide sheets were prepared to verify this technique. Au‐doped FeNi oxyhydroxides were fabricated by constructing the FeNi hydroxides on Ni foam by electrochemical deposition using Fe/Ni ions precursors. Second, the FeNi hydroxides were converted to FeNi oxyhydroxides by an OER process in 1 m KOH. Many problems were encountered during the fabrication of controlled and active FeNi oxyhydroxides. First, FeNi hydroxides formed, and their catalytic OER activity was not reproducible under similar conditions. For the fabrication of FeNi hydroxides, the current density curves were also different at a similar applied potential (**Figure** **34**A–F). Second, the electrodeposition method was followed to implant Au atoms in defective sites of FeNi oxyhydroxides and perform their ORR activities (**Figure** **35**A–C). In addition, the catalytic ORR activities of Au‐doped FeNi (oxy)hydroxides are not reproducible (**Figure** **36**A–C). Based on the above results, the electrochemical approach can synthesize SMAs, and the catalytic activities of SMAs with similar active sites are not achieved. Therefore, researchers should report the electrochemical data (applied current or voltage curves) for deposition (three times) and their catalytic performance at least three times. Lastly, many large current densities (1 A cm^−2^) for the OER have been reported but reproducibility is a major problem under alkaline solution. In addition, stability is achieved owing to 3D Ni/Fe/Cu foams. Therefore, all activity data displayed based on the metal foams are the pseudo current density. Furthermore, stability is directly affected by the metal foams. Therefore, the electrocatalytic activity in terms of overpotential can be considered the “pseudo‐performance” because such supported catalysts have high surface areas and abundant metal (Ni/Fe/Cu) active sites.

### 7.2 Activity Enhancement

The second critical parameter is the catalytic activity of the synthesized materials, which is critical to the device performance. Therefore, developing stable catalysts with high activity for various chemical reactions is essential for energy and non‐energy‐related applications. The catalytic efficiencies are altered by rationally modifying the coordination environments of single‐ to multi‐metal atoms. The high‐density SMAs exhibited enhanced activities compared to the metal NP‐based catalysts. On the other hand, high‐density SMAs are aggregated during the preparation and reaction processes. Low‐density SMAs are needed to prevent aggregation. Nevertheless, the tiny metal loading has an unsatisfactory metal site density, resulting in unpleasant overall catalytic performance. Therefore, developing low‐density SMAs with high catalytic activity is the key challenge, a long way from real‐life applications. Multifunctional catalysts that co‐anchor two, three, or more metal atoms on a suitable surface could be useful to solve these problems because of further tuning of the geometrical configurations and electronic properties (modulate *d*‐band center and the charge distribution) of the materials. In addition, DMAs/TMAs/MMAs offer more prospects to optimize the binding strength of intermediates and enhance the catalytic activity and selectivity. Many researchers have started to work on the design and synthesis of DMA/TMAs/MMAs coordinated with C and N, S, P, B, and O species. Thus far, one of the biggest challenges is fabricating effective multifunctional catalysts that co‐anchor two, three, or more metal atoms on a suitable surface.

### 7.3 Computational Studies

DFT,^[^ ^469^ ^]^ molecular dynamics (MD),^[^ ^470^ ^]^ and machine learning (ML)^[^ ^471^ ^]^ calculations of SMAs/DMAs/TMAs/MMAs have provided vital information on the active catalytic sites of metal atomic catalysts that help explain the superior catalytic conversions and chemical yields. Combining these simulations also revealed new mechanisms and reaction pathways, which have shed light on the structure‐property relationship for catalytic reactions at the atomic/electronic level. Based on a consistent set of calculations, activity descriptors were developed for different chemical reactions, further guiding the rational design and the rapid screening of SMAs/DMAs/TMAs/MMAs for specific applications. The reported descriptors are based on adsorption‐free energies, which are difficult to measure experimentally and generally require large amounts of computation space and time. Therefore, efficient, low‐cost, and universal descriptors for SMAs/DMAs/TMAs/MMAs (simply looking at the handbook/database or performing low‐cost computing) are needed because they can reliably predict the catalytic performance. Moreover, such a descriptor must have some physical properties that help better understand the property‐reactivity relationships of catalysts.

Furthermore, the theoretical model must be well matched with experimental characterization to simulate the catalytic reaction pathways effectively and develop new mechanisms. Over the years, theoretical studies have made significant progress in investigating experimental phenomena and designing SMAs/DMAs/TMAs/MMAs. On the other hand, most simulated models were not matched with experimental atomic structures of materials, and the simulations were performed under conditions close to ultra‐high vacuum. Under experimental conditions, the situation is more complicated because of different metal atoms, various heteroatom species, pH, and solvation. Therefore, the theoretical simulation must be carried out under the effect of solvation, while calculating the energies must include the pH conditions.

### 7.4 Interpretation of In Situ (Operando) Catalytic Processes

Ex situ spectroscopy and microscopy are needed to identify the local environment information and electronic details of materials. Furthermore, coordination numbers (metal‐to‐metal or metal‐to‐heteroatom bindings) should also be recognized using advanced spectroscopic tools. The core‐first‐order shell distance used to identify the atomic and electronic structures of the single‐, di‐, tri, and multi‐atom sites (SMAs/DMAs/TMAs/MMAs) provides an essential reference for interpreting theoretical models that can show the actual reaction conditions. Furthermore, combining DFT, ML algorithms, molecular dynamics, reaction force field, neural network, and microkinetics is a promising direction for complex multiscale modeling and simulations to gain a deeper understating of the structure‐activity properties of SMAs/DMAs/TMAs/MMAs.

In situ (or operando) materials analysis in different electrolytes can also identify changes in the configuration of SMAs/DMAs/TMAs/MMAs under ongoing catalytic reactions. These studies have provided fundamental insights and opened opportunities to elucidate the charge transport mechanism under real‐time catalytic performance. The most useful in situ characterization techniques to reflect the mechanistic details of SMAs/DMAs/TMAs/MMAs are X‐ray absorption spectroscopy (XAS), high‐resolution XPS, mass spectrometry (MS), FTIR, and Raman spectroscopy. Each of these techniques provided different physical and chemical information during the catalytic reactions. For example, XAS/XPS provides element‐, state‐, and coordination‐sensitive information. Raman/FTIR spectroscopy provides information on the adsorbed intermediates and surrounding ligands. MS identifies the reaction intermediates/products. Among them, in situ XAS is more powerful for understanding the fundamental physicochemical interactions at the SMAs/DMAs/TMAs/MMAs surfaces. The major challenges in this field will be the combined analysis of XAS/XPS/FTIR/Raman/MS. The secondary challenge is fabricating and designing a cell set‐up that can overcome the problems of mass‐/heat‐transfer and diffusions under harsh conditions. The third future challenge is atomic‐level analysis by in situ STEM/TEM, where the experiment is performed under very sophisticated conditions.

### 7.5 Practical Applications

Electrochemical water splitting technologies include AWE, PEMWE, AEMWE, and SOEC, which consist of two coupled half‐reactions: the hydrogen evolution reaction (HER; produce hydrogen) and the oxygen evolution reaction (OER; produce oxygen).^[^ ^472^, ^473^, ^474^ ^]^ Because green hydrogen is generated from these technologies, it can reduce environmental pollution and has high‐energy conversion efficiency. HER/OER materials are essential to the industrial application of electrochemical water splitting. Furthermore, it is highly desirable to synthesize transition metal‐based and robust electrocatalysts, especially at large current densities.^[^ ^475^, ^476^, ^477^ ^]^ These problems can be alleviated using SMA/DMA/TMA/MMA‐based materials to enhance intrinsic activity and improve atomic utilization. Thus, SMA/DMA/TMA/MMA‐based materials exhibited excellent activity and durability toward the HER/OER in water splitting under a wide pH range. The high HER/OER activities were attributed to modifying the electronic configuration at active centers by introducing metal atoms. Despite the considerable efforts to develop SMA/DMA/TMA/MMA‐based materials for water splitting, several issues still need to be overcome. First, high mass and areal activities must be achieved via increases in the active species loading densities of efficient exposed metal‐atom in SMAs/DMAs/TMAs/MMAs. Second, the biggest challenge lies in the unsatisfactory stability of SMA/DMA/TMA/MMA‐based materials during water splitting, particularly, at ultra‐high‐density metal atoms with loadings above 0.5 wt%. Once these issues are solved, the SMA/DMA/TMA/MMA‐based materials will be a strong candidate for industrial water splitting.

Fuel cells maintaining high performance at large current densities depend heavily on the cathode materials, where the sluggish ORR kinetics reduce the energy efficiency (cell voltage). At the same time, low oxygen mass transfer and poor proton conduction (the ionomer or the water) restrict the power density. Therefore, more efforts are needed to boost the intrinsic catalytic activity via novel SMA/DMA/TMA/MMA‐based materials. SMA/DMA/TMA/MMA‐based materials tend to aggregate during the ORR reaction processes, which remains a challenge. Nevertheless, the ORR activity is unsatisfactory if low‐loading metal atoms are adopted. On the other hand, the loading of high‐metal content atoms causes problems with mass transport. Thus, the insufficient metal active site is still a major obstacle to overcome before practical applications can be achieved. A strong interaction occurs between the metal atoms and supports, enhancing the durability, but a very strong bond affects the electronic structure (4d/5d metal‐modulated electronic configuration and optimized adsorption ability) of metal atoms. Nevertheless, MEA stability is also a formidable challenge for numerous ORR materials because they work at high temperatures (60–80 °C), which are much higher than the rotating disk electrode (RDE) measurements. Intensive efforts are needed to examine the membrane electrode assembly (MEA)‐level activities of SMA/DMA/TMA/MMA‐based ORR materials and their power efficiency in fuel cell stacks.

Like fuel cells, the power efficiency of ZABs is governed mainly by the cathodic ORR materials,^[^ ^478^ ^]^ while the ZAB setup is more straightforward than fuel cells. The ORR activities are improved by SMA/DMA/TMA/MMA‐based materials because of the modification of the metal center (metal 3*d* electron delocalization with *d* band center upshift). On the other hand, the energy density of the ZABs has not reached the theoretical energy density. These materials also suffer from poor electrical conductivity, sluggish Li transport, low thermal durability, high volume expansion, and mechanical brittleness. Therefore, research should be done on SMA/DMA/TMA/MMA‐based materials to overcome the limitations of cost, energy density, power density, cycle life, and safety. New materials based on metal atoms can break the above boundaries and have a greater impact on human life.

Considering the current challenges for practical applications of electrochemical H_2_O_2_ production, a new type of SMA/DMA/TMA/MMA‐based material delivers the following novel and innovative solutions. i) SMA/DMA/TMA/MMA‐based materials can convert oxygen into H_2_O_2_ because of the regulated electronic structure through active sites of metal atoms. ii) Low‐cost materials can also be developed using SMA/DMA/TMA/MMA‐based materials. iii) H_2_O_2_ production with high yield and selectivity can be improved further by modulating the compositions of SMA/DMA/TMA/MMA‐based materials and custom‐designed systems. This conclusion is based on laboratory‐scale results, which have only short‐time stability and small‐scale materials production, resulting in far‐from‐practical applications. Therefore, researchers need to collaborate with industrial laboratories to improve the material dispersibility, large‐scale material production, new electrolyte design, new reactor fabrication, increase volumetric area, improve long‐term stability, and verify the industrial tests.

Regarding CO_2_RR, the activities of CO_2_RR have been improved significantly through SMA/DMA/TMA/MMA‐based materials. The geometric (symmetric or asymmetric structures) and electronic configurations of SMA/DMA/TMA/MMA‐based materials are generally affected by parameters, such as the type of the metal atoms, nature of bonding atoms (C, N, S, P, B, and O), coordination numbers, and secondary coordination spheres, leading to immense tasks in the recognition structure−activity relationships and the main reaction mechanisms. Thus far, most metal atoms can accelerate formate production via CO_2_RR, but high overpotentials are needed to activate CO_2_ on the SMA/DMA/TMA/MMA‐based materials. This leads to errors in the faradic efficiency calculations because the yield of converting CO_2_ to fuels depends on the CO_2_ gas flow rates.^[^ ^479^ ^]^ Therefore, further SMA/DMA/TMA/MMA‐based material designs to adjust CO_2_‐to‐formate conversion and enhance energy efficiency at large current densities are appropriate for possible practical applications.

In the case of NRR, significant and encouraging progress has been made in converting the gaseous N_2_ molecule to high‐value‐added NH_3_. On the other hand, deploying them in practical applications is still a major challenge. Recently, research has focused mainly on exploring SMA/DMA/TMA/MMA‐based materials for the NRR. These materials can improve the reaction rate, efficiency, and selectivity toward NH_3_ production. Despite this, most materials exhibited efficiency for short periods (as rapid loss in activity and selectivity for most SMA/DMA/TMA/MMA‐based materials reported). They were not examined on an industrial timescale. Therefore, laboratory discoveries are very difficult to transfer to industrial use. Thus, it is essential to make NRR the core technology in a sustainable environment.

Research on highly reliable and cost‐effective SMA/DMA/TMA/MMA‐based sensors has rapidly increased worldwide because it adjusted the electronic and chemical properties. Nevertheless, sensors based on SMAs/DMAs have reported more literature, while sensors based on TMAs/MMAs have not been reported. The catalytic activity of SMAs/DMAs showed up to date is inferior to NPs, suggesting that rigorous research and innovation in structure would be an encouraging way to enhance the sensing performance of SMAs/DMAs. More work on TMAs/MMAs will be needed to enable their use in sensors. Thus, the practical use of the sensors is far from reality, and studies are still at the initial stage with narrow applications.

Although remarkable progress has been achieved for SAzymes, DAzymes, TAzymes, and MAzymes (SMA/DMA/TMA/MMA)‐based materials, several challenges remain to be solved for the development of pharmaceutical industries. For example, the prolonged biosafety and cell viability of the SAzymes, DAzymes, TAzymes, and MAzymes is one of the major problems. The second issue is that translating laboratory findings to human‐life implantation is vital for producing SAzymes, DAzymes, TAzymes, and MAzymes‐based materials in clinical applications. Biosafety and cell viability can be improved by abundant functional groups, which have been applied in various biomedical uses. In the future, SAzymes, DAzymes, TAzymes, and MAzymes will play an important role in disease early sensing, deep tissue bioimaging, and multi‐functional controlled therapy.

## Conflict of Interest

The authors declare no conflict of interest.
